# ﻿Systematic revision of the ant subfamily Leptanillinae (Hymenoptera, Formicidae)

**DOI:** 10.3897/zookeys.1189.107506

**Published:** 2024-01-16

**Authors:** Zachary Griebenow

**Affiliations:** 1 Department of Entomology & Nematology, University of California, Davis, CA USA University of California Davis United States of America; 2 Department of Agricultural Biology, Colorado State University, Fort Collins, CO USA Colorado State University Fort Collins United States of America

**Keywords:** Morphology, phylogenetics, subterranean biology, taxonomy

## Abstract

The genus-level taxonomy of the ant subfamily Leptanillinae (Hymenoptera: Formicidae) is here revised, with the aim of delimiting genus-level taxa that are reciprocally monophyletic and readily diagnosable based upon all adult forms. This new classification reflects molecular phylogenetics and is informed by joint consideration of both male and worker morphology. Three valid genera are recognized in the Leptanillinae: *Opamyrma*, *Leptanilla* (= *Scyphodon***syn. nov.**, *Phaulomyrma*, *Leptomesites*, *Noonilla***syn. nov.**, *Yavnella***syn. nov.**), and *Protanilla* (= *Anomalomyrma***syn. nov.**, *Furcotanilla*). *Leptanilla* and *Protanilla* are further divided into informal, monophyletic species groups. Synoptic diagnoses are provided for all genera and informal supraspecific groupings. In addition, worker-based keys to all described species within the Leptanillinae for which the worker caste is known are provided; and male-based keys to all species for which males are known, plus undescribed male morphospecies for which molecular data are published. The following species are described as new: *Protanillawallacei***sp. nov.**, *Leptanillaacherontia***sp. nov.**, *Leptanillabelantan***sp. nov.**, *Leptanillabethyloides***sp. nov.**, and *Leptanillanajaphalla***sp. nov.**

## ﻿Introduction

The subfamily Leptanillinae (Hymenoptera: Formicidae), sometimes called legionary vampire ants (Ward and Boudinot 2021), consists of cryptic, hypogaeic ants largely restricted to tropical and warm temperate regions of the Old World, although *Protanillabeijingensis* Man, Ran, Chen & Xu, 2017 and *Leptanillataiwanensis* Ogata, Terayama & Masuko, 1995 have been collected in a cold temperate climate. Most of their diversity is concentrated in the Indo-Malayan region. While the affinities of the Leptanillinae to other ants have historically been controversial, phylogenetic inference from molecular data that corrects for compositional heterogeneity in nucleotides supports the monotypic Neotropical genus *Martialis* Rabeling & Verhaagh as the sister group of the Leptanillinae, with this clade collectively being sister to all other extant Formicidae (Borowiec et al. 2019; Romiguier et al. 2022).

The internal taxonomy of the Leptanillinae has been afflicted with probable parallelism, since males are collected more often than workers or gynes: both genus- and species group names were established based solely upon male specimens. The sexes are only directly associated in *L.japonica* Baroni Urbani, 1977 (Ogata et al. 1995) and *Opamyrmahungvuong* Yamane, Bui & Eguchi, 2008 (Yamada et al. 2020), while Griebenow (2020) associated the sexes of *Protanillalini* Terayama, 2009 with phylogenomic inference. The genera *Scyphodon* Brues, *Noonilla* Petersen, and *Yavnella* Kugler were all described solely from male material, with the worker of *Yavnella* being identified ex post facto by phylogenomic inference (Griebenow et al. 2022). Total-evidence Bayesian inference recovered the male-based genus *Phaulomyrma* Wheeler & Wheeler within *Leptanilla* s. str. (Griebenow 2021), resulting in its synonymy under *Leptanilla*, with Griebenow (2020, 2021) delimiting *Leptanilla* s. l. to also include *Noonilla* and *Scyphodon*, with two major clades of *Leptanilla* s. l. known only from undescribed male morphospecies. The boundaries of *Leptanilla* relative to the three male-based genera must therefore be formally revised. Generic boundaries in the former Anomalomyrmini require revision as well, with phylogenetic inference consistently recovering *Protanilla* as paraphyletic relative to *Anomalomyrma* irrespective of dataset or statistical framework (e.g., Borowiec et al. 2019; pers. obs.).

Colonies of *Protanillajongi* Hsu, Hsu, Hsiao & Lin, 2017 and *Leptanillabelantan* sp. nov. were collected in decaying wood (Hsu et al. 2017; this study), and foraging workers of *Protanillalini* Terayama, 2009 in Sea, Land and Air Malaise (SLAM) traps (Griebenow 2020), but leptanilline workers are otherwise exclusively subterranean. Based on limited observations of live colonies, it appears that leptanilline ants are specialized predators of geophilomorph centipedes or forcepstails (Diplura: Japygidae) (Masuko 1990; Hsu et al. 2017; Ito et al. 2022), with *P.lini* feeding on other prey (e.g., lithobiomorph centipedes, cockroaches) in captivity (Katayama and Tsuji 2011; Yamamuro 2018). *Leptanilla* display aspects of the “army ant syndrome” commonly associated with *Dorylus*, *Eciton*, and related lineages in the subfamily Dorylinae: *Leptanillajaponica* Baroni Urbani, 1977 and *Leptanillaclypeata* Yamane & Ito, 2001 engage in synchronized brood production (Masuko 1990; Ito and Yamane 2020) and regular colony migration, with the physogastry reported in *Leptanillacharonea* Barandica, López, Martínez & Ortuño, 1994 and *Leptanillazaballosi* Barandica, López, Martínez & Ortuño, 1994, indicating synchronized brood production in at least those species as well (López et al. 1994). Gynes of *Leptanilla* are always wingless and blind. It is unclear whether *Protanilla* (the only other leptanilline genus for which any bionomic data are available) display legionary behavior, but the alate condition of *Protanilla* gynes (except for *Protanillawallacei* sp. nov.; see Billen et al. 2013; Ito et al. 2022) contradict this assumption. Intracolonial uniformity of larval instar in *Protanillagengma* Xu, 2012 (pers. obs.) indicates synchronized brood production in at least that species. Gynes of *L.japonica* and *L.clypeata*, and the worker of *L.clypeata*, engage in larval hemolymph feeding (LHF) via a specialized “larval hemolymph tap” (Masuko 1989) that acts as an exudatorium (Wheeler 1918), facilitating non-traumatic LHF (Masuko 1989; Ito and Yamane 2020); such an exudatorium is otherwise known in ants only in *Proceratiumitoi* (Forel, 1918) (Proceratiinae) (Masuko 2019). Larvae of *Leptanilla* bear a prothoracic process (Wheeler 1918; Kugler 1987; Wheeler and Wheeler 1988; Barandica et al. 1994) that is used as a grip by workers during colony migration (Masuko 1990). The larvae of *P.jongi* examined in this study lack this process.

With the internal phylogeny of the tribe Leptanillini confidently resolved by a combination of total-evidence and phylogenomic approaches (pers. obs.), including the identification of workers of *Yavnella* and *Scyphodon* s. l., worker and male morphology can be contextualized on this robust phylogeny. Therefore, the time is ripe for revision of the Leptanillinae at the genus level. What follows is a systematic revision of the subfamily to establish reciprocally monophyletic and consistently diagnosable genera and species groups. *Protanillawallacei* sp. nov., *Leptanillaacherontia* sp. nov., and *Leptanillabelantan* sp. nov. are described based upon worker specimens. To provide a formal name for the Bornean morphospecies group of *Leptanilla* s. l. (Griebenow 2020, 2021), known only from bizarre males, *Leptanillanajaphalla* sp. nov. is described based solely upon male specimens. Likewise, to establish a formal name for the Indochinese morphospecies group (Griebenow et al. in press), *Leptanillabethyloides* sp. nov. is described based on male specimens. The first global worker-based keys to all species of the Leptanillinae are also provided, with male-based species-level keys.

## ﻿Materials and methods

Specimens were imaged using the same equipment as reported in Griebenow (2020, 2021) and Griebenow et al. (2022), with the addition of a VHX-970F digital microscope (Keyence, Osaka, Japan). Accession numbers and a subset of collection data for all specimens consulted in this study not previously included in Griebenow (2020, 2021) or Griebenow et al. (2022) are provided in Suppl. material 1:

**BPBM**Bernice P. Bishop Museum, Honolulu, USA;

**CAS**California Academy of Sciences, San Francisco, USA;

**CSCA**California State Collection of Arthropods, Sacramento, USA;

**HKUBM** Biodiversity Museum, University of Hong Kong, China;

**JAZM**Jalal Afshar Zoological Museum, Department of Plant Protection, College of Agriculture and Natural Resources, University of Tehran, Karaj, Iran;

**LACM**Los Angeles County Museum of Natural History, Los Angeles, USA;

**MCZC**Museum of Comparative Zoology, Cambridge, USA;

**MHNG**Muséum d’Histoire Naturelle, Geneva, Switzerland;

**MZLS**Museé Zoologique, Lausanne, Switzerland;

**MZLU**Lund University, Lund, Sweden;

**NCUE** National Changhua University of Education, Changhua, Taiwan;

**OIST** Okinawa Institute of Science and Technology, Onna-son, Japan;

**ROME**Royal Ontario Museum, Toronto, Canada;

**UCDC**R. M. Bohart Museum of Entomology, University of California, Davis, USA;

**ZMHB**Museum für Naturkunde der Humboldt-Universität, Berlin, Germany;

**TAU**Tel Aviv University, Tel Aviv, Israel;

**ZMUI** Zoological Museum, University of Isfahan, Isfahan, Iran.

I also consulted the personal collections of José María Gómez-Durán, John T. Longino, and Philip Ward. Discrepancy in provisional morphospecies identifiers with those used in previous studies is resolved by Table 1.

**Table 1.** Concordance of morphospecies identifiers used in this study that conflict with Griebenow (2020, 2021), Griebenow et al. (2022), and Griebenow et al. (in press).

Current identifier	Previous identifier
*Leptanilla* MM01	*Yavnella* MM01
*Leptanilla* TH02	*Yavnella* TH02
*Leptanilla* TH03	*Yavnella* TH03
*Leptanilla* TH04	*Yavnella* TH04
*Leptanilla* TH06	*Yavnella* TH06
*Leptanilla* TH07	*Leptanilla* TH07
*Leptanilla* TH08	*Yavnella* TH08
*Leptanilla* zhg-bt03	*Yavnella* zhg-bt01
*Leptanilla* zhg-mm14	*Yavnella* indet.
*Leptanillanajaphalla*	*Leptanilla* zhg-my02
*Leptanilla* zhg-my10	*Noonilla* zhg-my01
*Leptanilla* zhg-my11	*Noonilla* zhg-my02
*Leptanilla* zhg-my14	*Noonilla* zhg-my06
*Leptanilla* zhg-my16	*Yavnella* zhg-my02
*Leptanilla* zhg-th02	*Yavnella* zhg-th01
*Leptanilla* zhg-th04	*Yavnella* zhg-th03
*Leptanilla* zhg-th05	*Yavnella* zhg-th04
*Protanillagengma*	*Protanilla* VN01
*Protanilla* id01	*Anomalomyrma* indet.

### ﻿Measurements

Definitions pertain to all adult forms unless otherwise noted.

**HW** Head Width, maximum width of cranium in full-face view, including compound eyes if present;

**HL** Head Length, maximum length of head in full-face view from anterior margin of head capsule to cranial vertex;

**EW** Eye Width, maximum breadth of compound eye measured perpendicular to anteroposterior axis of head (male);

**EL** Eye Length, maximum length of compound eye measured parallel to anteroposterior axis of head (male);

**SL** Scape Length, maximum length of scape in medial view, excluding bulbus;

**LF2** Third Antennomere Length, length of the basal flagellomere;

**ML** Mandible Length, maximum length of mandible from view orthogonal to lateral mandibular margin, measured from ventral mandibular articulation to mandibular apex;

**MaL** Mandalar Length, maximum length of mandalus, measured along proximodistal axis of mandible;

**WL** Weber’s Length, maximum diagonal distance measured from most anterior extent of pronotum excluding (female) or including (male) cervical shield to most posteroventral extremity of the mesosoma, including propodeal lobes if present;

**PrW** Pronotal width, maximum width of pronotum, measured in dorsal view;

**MW** Mesonotal width, maximum width of mesonotum in dorsal view, measured immediately anterior to mesocoxal foramina;

**MSW** Mesoscutal width, maximum width of mesoscutum in dorsal view (male);

**MSL** Mesoscutal length, maximum length of mesoscutum in dorsal view (male);

**PTL** Petiolar length, maximum length of petiole in dorsal view, not including presclerites;

**PTH** Petiolar height, maximum height of petiole in profile view, including sternal process and dorsal node, if distinct;

**PTW** Petiolar width, maximum width of petiole in dorsal view orthogonal anteroposterior axis;

**PPL** Postpetiolar length, maximum length of postpetiole in dorsal view, not including presclerites;

**PPW** Postpetiolar width, maximum width of postpetiole in dorsal view;

**PPH** Postpetiolar height, maximum height of postpetiole in profile view, including sternal process and dorsal node, if distinct;

**TW4** Width of abdominal tergite IV, maximum width of abdominal tergite IV measured in dorsal view.

### ﻿Indices

**CI** (HW / HL) × 100;

**SI** (SL / HW) × 100;

**MI** (ML / HW) × 100;

**OI** (EW / EL) × 100;

**MSI** (MSW / MSL) × 100;

**PI** (PTW / PTL) × 100;

**PPI** (PPW / PPL) × 100;

**TI1** (PPW / TW4) × 100.

### ﻿Nomenclature

**Figure 1.**
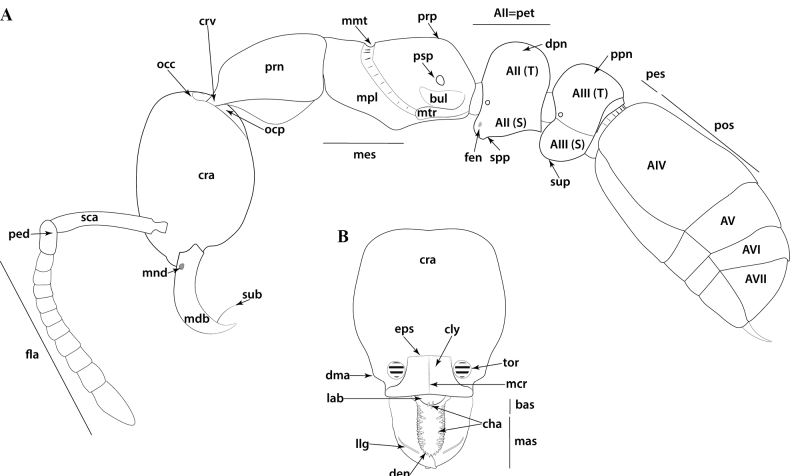
Glossary of morphological terms used to describe the worker soma in the Leptanillinae, with *Protanillabeijingensis* as template **A** profile habitus **B** full-face view. Abbreviations: A = abdominal segment; bas = basal mandibular margin; bul = bulla; cha = chaetae; cly = clypeus; cra = cranium; crv = cervical shield; den = denticle; dma = dorsal mandibular articulation; dpn = petiolar node; eps = epistomal sulcus; fen = fenestra; fla = flagellum; lab = labrum; llg = laterodorsal longitudinal groove; mas = masticatory mandibular margin; mcr = median clypeal ridge; mdb = mandible; mes = mesothorax; mmt = meso-metapleural suture; mnd = mandalus; mpl = mesopleuron; mtr = metapleural trench; occ = occipital carina; ocp = occiput; ped = pedicel; pes = presternite; pos = poststernite; ppn = postpetiolar node; prn = pronotum; prp = propodeum; psp = propodeal spiracle; S = sternite; sca = scape; spp = subpetiolar process; sub = subapical mandibular seta; sup = sub-post-petiolar process; T = tergite; tor = torulus.

**Figure 2.**
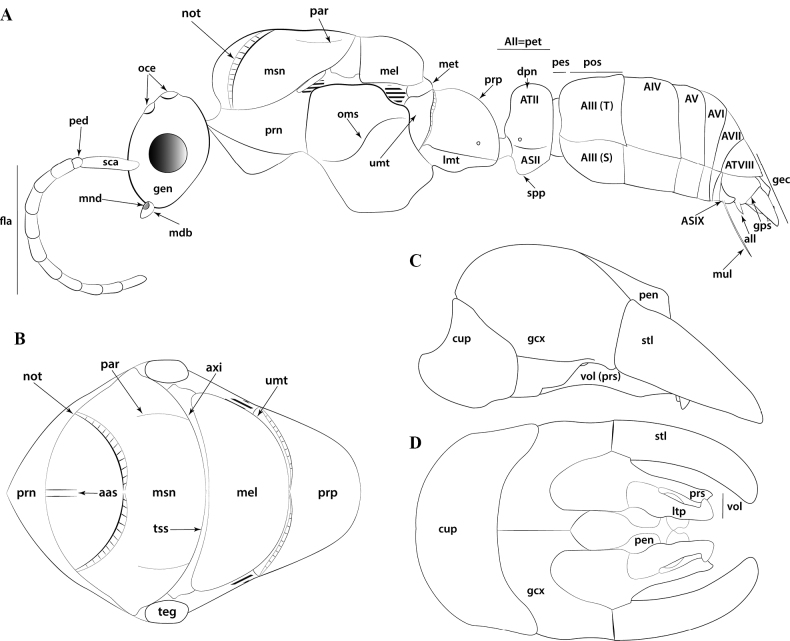
Glossary of morphological terms used to describe male morphology in the Leptanillinae. Figure A, B is chimeric, but *Protanilla* zhg-vn01 is the template for Fig. 2C, D**A** profile habitus **B** mesosomal dorsum **C** genitalia, profile view **D** genitalia, ventral view. Abbreviations: A = abdominal segment; aas = antero-admedian signum; all = apicolateral gonocoxital lamina; axi = axilla; cup = cupula; dpn = petiolar node; fla = flagellum; gcx = gonocoxites; gec = genital capsule; gen = gena; gps = gonopodital suture; ltp = lateropenite (=digitus); mdb = mandible; mel = mesoscutellum; met = metascutellum; mnd = mandalus; msn = mesonotum; mul = mulceators; not = notauli; oce = ocelli; oms = oblique mesopleural sulcus; par = parapsidal signa; ped = pedicel; pen = penial sclerites; pes = presternite; pet = petiole; prn = pronotum; prp = propodeum; prs = parossiculus (= cuspis *partim*); S = sternite; sca = scape; spp = subpetiolar process; stl = gonostylus; T = tergite; teg = tegula; tss = transscutal line; umt = upper metapleuron; vol = volsella.

**Figure 3.**
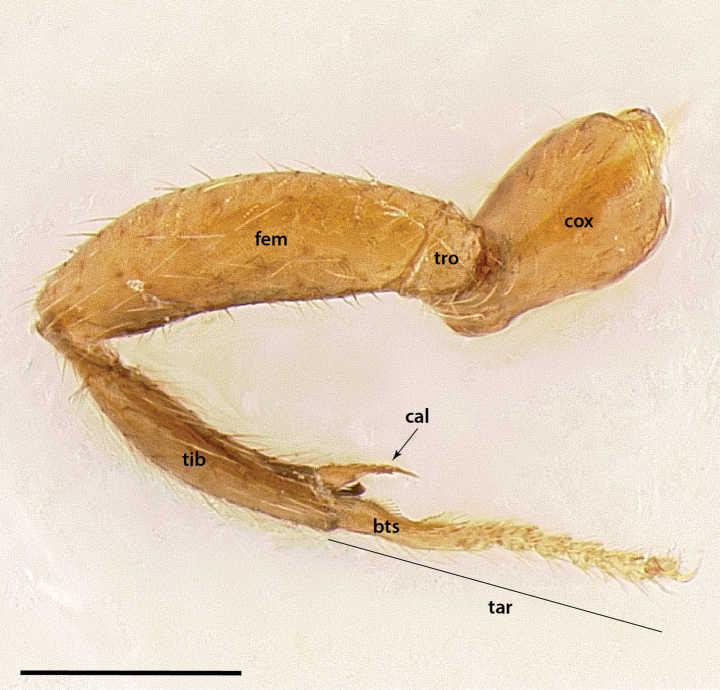
Glossary of leg nomenclature used for the Formicidae, with the male foreleg of *Leptanilla* zhg-my11 (CASENT0842593) as template. Abbreviations: bts = basitarsus; cal = calcar; cox = coxa; fem = femur; tar = tarsus; tib = tibia; tro = trochanter. Scale bar: 0.2 mm.

Nomenclature for sculpture and setation combines Harris (1979), Wilson (1955), and Boudinot et al. (2020). Notational conventions for palp and tibial spur formulae follow Bolton (2003). Cephalic nomenclature follows Richter et al. (2021) and Boudinot et al. (2021). Mesosomal nomenclature follows Liu et al. (2019); metasomal, Lieberman et al. (2022). Male genital nomenclature follows Boudinot (2018). Descriptive terms for larval morphology follow Wheeler and Wheeler (1986, 1976). Wing venation is described using Brown and Nutting (1949) and Ogata (1991), with interpretation of homologies in male wing venation following Boudinot (2015) in some ambiguous cases observed in *Leptanilla*. Any morphological terms unaddressed in these publications follow the Hymenoptera Anatomy Ontology (Yoder et al. 2010). Glossaries of external morphological terms for worker and male Leptanillinae are summarized in Figs 1–3. In instances where the homology of the terminal abdominal sternite is ambiguous, this sternite is termed a hypopygium.

### ﻿Species concept

I here follow Barraclough (2019) in treating a species as an evolutionarily independent population of organisms that is genetically and phenotypically distinct from other such populations (Simpson 1961). In sexually reproducing organisms, such as the Leptanillinae (so far as is known), reproductive isolation sufficient to maintain interspecific distinctiveness—in other words, the absence of genotypic and phenotypic intermediates—is an expected property of species. Mechanically incompatible genitalia are an expected corollary of reproductive isolation, and thus would indicate interspecific differentiation, but may only be asserted to be so for sibling populations that occur in sympatry and exhibit consistent phenotypic differentiation. The degree of differentiation between such species serves as a “yardstick” by which to assess whether allopatric populations diverge sufficiently in phenotype to be considered heterospecific (Tobias et al. 2010; Ward and Branstetter 2022). Scenarios that allow this calibration of phenotypic difference are fulfilled thrice among the leptanilline morphospecies for which UCEs have been successfully enriched: one instance being *Leptanillanajaphalla* sp. nov. and *Leptanilla* zhg-my05 (Sabah, Malaysia); another, *Leptanillacharonea* and Leptanillacf.zaballosi (Madrid, Spain); and the last, *Leptanilla* zhg-bt01 and -02 (Bhutan). In all cases the two putative sympatric species are recovered as closely related terminals by phylogenomic inference (Griebenow 2020, 2021; Griebenow et al. 2022), and males of each species pair exhibit a phenotype uniformly distinguishable across all available specimens by the proportions of the genitalia. Variation among the syntopic specimen series assigned to these morphotypes is bimodal, with the exceptions to this bimodality not constituting intermediates. Thus, there is no indication that any differentiation in genital shape among these sympatric species can be considered intraspecific.

## ﻿Results

### ﻿ Protanilla wallacei sp. nov.

Taxon classificationAnimaliaHymenopteraFormicidae

13D818D1-E9B5-55E6-AB3C-39D6C80169DC

https://zoobank.org/6AC428A6-E31D-412A-93E4-9E0BCF7B716E

Fig. 4A–C

#### Type material.

***Holotype*.** Malaysia – Sarawak • 1 worker; Gunung Mulu National Park, 4^th^ division; 4.09°N, 114.89°E (estimated from Google Earth to nearest minute); May–Aug. 1978, P. M. Hammond and J. E. Marshall leg.; CASENT0902782; BM1978–49, BMNH(E) 1015826. BMNH. ***Paratype*.** Malaysia – Sabah • 1 worker; Gunung Silam, Lahad Datu; 4.96°N, 118.17°E (estimated from Google Earth to nearest minute); 630m a.s.l.; 1983; R. Leakey leg; CASENT0842699; UCDC.

#### Other material examined.

Malaysia – Sabah • 1 worker; 8km S Sapulut, 4.62844°N, 116.47175°E; 325m a.s.l.; 31.vii.2014; P. S. Ward leg.; sifted litter (leaf mold, rotten wood), rainforest; CASENT0842640; PSW17199–01. UCDC.

#### Measurements (mm) and indices.

***Holotype***: N/A ***Paratype***: HL = 0.42; HW = 0.33; SL = 0.22; PW = 0.27; WL = 0.68; PTL = 0.2; PTW = 0.19; PPTL = 0.19; PPTW = 0.2; CI = 79; SI = 106; PI = 98; PPI = 113. **Other material examined** (*n* = 2): HL = 0.43–0.46; HW = 0.35–0.36; SL = 0.33–0.39; ML = 0.21–0.24; PW = 0.26–0.29; WL = 0.64–0.72; PTL = 0.19–0.21; PTW = 0.2; PPTL = 0.19–0.21; PPTW = 0.2–0.23; CI = 78–80; SI = 97–102; PI = 93–101; PPI = 105–108

#### Description.

Lateral cranial margins converging anteriorly; cranium not bulging towards vertex. Genal angle laterad antennal toruli obtuse. Outline of clypeus campaniform in full-face view, laterally elevated above cranium, posteriorly not elevated above frons; clypeal surface planar; anterior clypeal margin slightly emarginate, posteromedian clypeal margin emarginate; median clypeal ridge present on mesal surface of clypeus, externally visible. Labrum visible in full-face view; anterodorsal apex of labrum armed with three or four dentiform, peg-like chaetae; venter with vestiture of suberect lanose setae. Mandibles elongate relative to head (CI = 79–80), linear, apex curved downward distally; vertical dorsal lamella absent; laterodorsal longitudinal groove present; dorsomedial margin of mandible with single row of ~ 12 dentiform, peg-like chaetae; lateral mandibular face glabrous. Labial palp 1-merous. Anterior tentorial pits faint, situated anterad the toruli, not visible in full-face view. Postgenal ridge complete. Scape long (SL 0.34–0.39 mm), reaching slightly beyond occipital margin when antennae retracted. Flagellum submoniliform; apical flagellomere 3× longer than broad. Pronotum broader than mesonotum in dorsal view, with lateral margins convex. Mesonotum narrow, with lateral margins parallel in dorsal view. Meso-metapleural suture narrow laterally, broader along dorsal surface; scrobiculate, with transverse ridges larger and more widely spaced along dorsal surface of meso-metapleural suture; posteriorly distinct from metapleural trench. Maximum breadth of metapectal-propodeal complex greater than that of mesonotum in dorsal view, slightly narrowed anteriorly, posterior outline convex in profile view. Bulla large, extending anterior to propodeal spiracle. Propodeum rounded in profile view. Tarsomeres longer than broad. Meso- and metatibial spur formula 0,1p. Petiole sessile. Abdominal segments II and III without tergotergal and sternosternal fusion. Abdominal segment II slightly longer than wide in dorsal view (PI 94–99), with distinct dorsal node, in profile view anterior and posterior faces subequal in height; anterior face of petiolar node linear in profile view. Subpetiolar process present, abdominal sternite II with concavity posterior to subpetiolar process so that margin of abdominal sternite II is sinuate in profile view; fenestra present, elliptical, anteroposteriorly compressed. Lengths of abdominal segments II–III subequal. Abdominal sternite II projecting no further than abdominal sternite III towards venter. Abdominal segment III slightly broader than long in dorsal view (PPI = 105–113), with distinct dorsal node; in profile view, anterior face of dorsal node abruptly vertical and bulging, posterior face gently sloping. Post-petiole with distinct tergosternal suture. Abdominal segments III–IV separated by pronounced constriction, with presclerites of abdominal segment IV distinct; pretergite IV planar in profile view, shorter than presternite IV; presternite IV slightly convex in profile view; cinctus of abdominal segment IV scrobiculate. Anterior margin of abdominal post-tergite IV shallowly emarginate in dorsal view. Outline of postpetiolar node trapezoidal in dorsal view, corners rounded, slightly narrowed anteriorly. Soma concolorous, color castaneous. Vestiture of suberect to erect setae present; length of setae variable.

**Figure 4.**
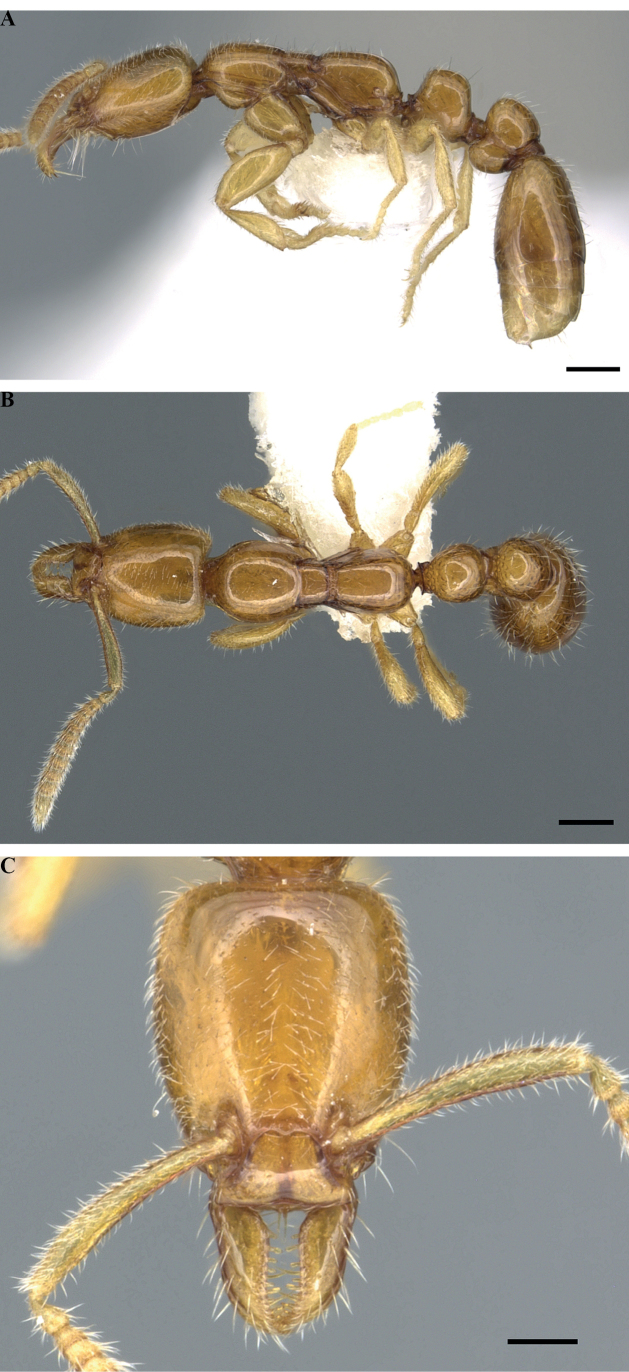
*Protanillawallacei*, holotype (CASENT0902782; Ziv Lieberman), worker **A** profile view **B** dorsal view **C** full-face view. Scale bars: 0.2 mm (**A, B**); 0.1 mm (**C**).

#### Etymology.

Named for Alfred Russel Wallace, commonly thought to be the progenitor of the discipline of biogeography and still well-regarded for his study of the biota of the Malay Archipelago, where this ant is native. The specific epithet is masculine, in genitive case.

#### Remarks.

**Figure 5.**
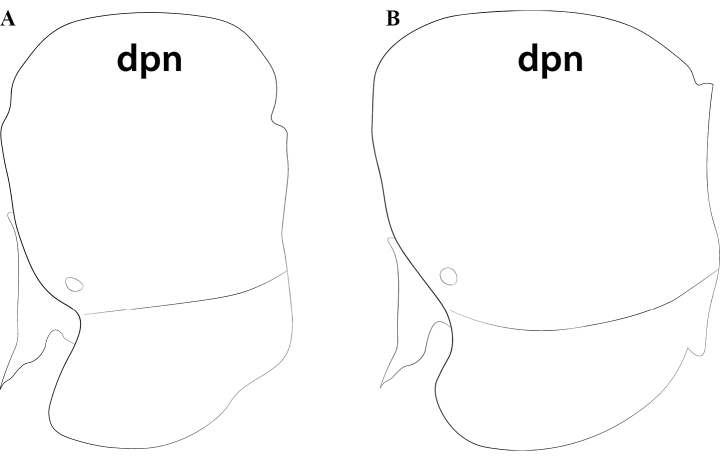
Worker petiole of *Protanillalini* (**a**) and *Protanillawallacei* sp. nov. (**b**), profile view. Abbreviation: dpn = petiolar node.

The worker caste of *P.wallacei* is extremely close to that of *P.lini* but differs in overall smaller size and the shallowness of the postpetiolar node, with the posterior declivity of the postpetiolar node being gradual (Fig. 5B) rather than abrupt (Fig. 5A). PPI tends to be greater in *P.wallacei* (x_ = 109) than in *P.lini* (x_ = 100) but cannot be consistently used to discriminate the two. Interestingly, all known gynes of *P.wallacei* are ergatoid (Billen et al. 2013; Ito et al. 2022), whereas those of *P.lini* are alate (Hsu et al. 2017).

*Protanillawallacei* appeared as a nomen nudum in Hölldobler and Wilson (1990), with the name purportedly being under description by Robert W. Taylor based upon material from Sabah. Such a description has not appeared. CASENT0842699 was identified as *P.wallacei* by Barry Bolton with reference to “type” material under description by Taylor, which, based on a paratype label assigned by Taylor, included CASENT0902782. Billen et al. (2013) described the glandular complement of specimens from peninsular Malaysia that was attributed to this nomen nudum by Taylor, while Ito et al. (2022) reported on the behavioral observations of specimens from that same series, referring to this species as *Protanilla* sp. *Protanillawallacei* is here made an available name, described based upon worker specimens from Sabah. Judging from Billen et al. (2013: fig. 5E), the series referred to in that study and in Ito et al. (2022) conforms to the diagnosis of *P.wallacei* here given. The unidentified *Protanilla* that was the sole representative of the Leptanillinae in the phylogenomic analyses of Branstetter et al. (2017) (CASENT0634862) is here identified as *P.wallacei*. *Protanillawallacei* shows intraspecific variation in labral chaeta count, which is also observed in putatively conspecific allopatric specimens of *P.gengma* (Aswaj et al. 2020; pers. obs.) and *P.beijingensis* (this study).

*Protanillawallacei* and *P.lini* are recovered as sister taxa in phylogenomic inference sampling from across the geographical range of the latter species (pers. obs.). *Protanillalini* ranges across Taiwan and the Ryukyu Islands, while the *P.wallacei* specimens examined in this study originate in the Sundan region. This allows for the possibility that these putative species are populations from extreme ends of a contiguous swath of metapopulations extending throughout southeast Asia. Further sampling in mainland southeast Asia may reciprocally efface the morphometric distinction between these species, and with the other members of the *Protanillalini* species complex.

### ﻿ Leptanilla belantan sp. nov.

Taxon classificationAnimaliaHymenopteraFormicidae

E42B0C80-D4E0-5A57-80C1-992580F02955

https://zoobank.org/3EB67585-11A5-418D-B30D-38A9440C92B3

**Figure 6.**
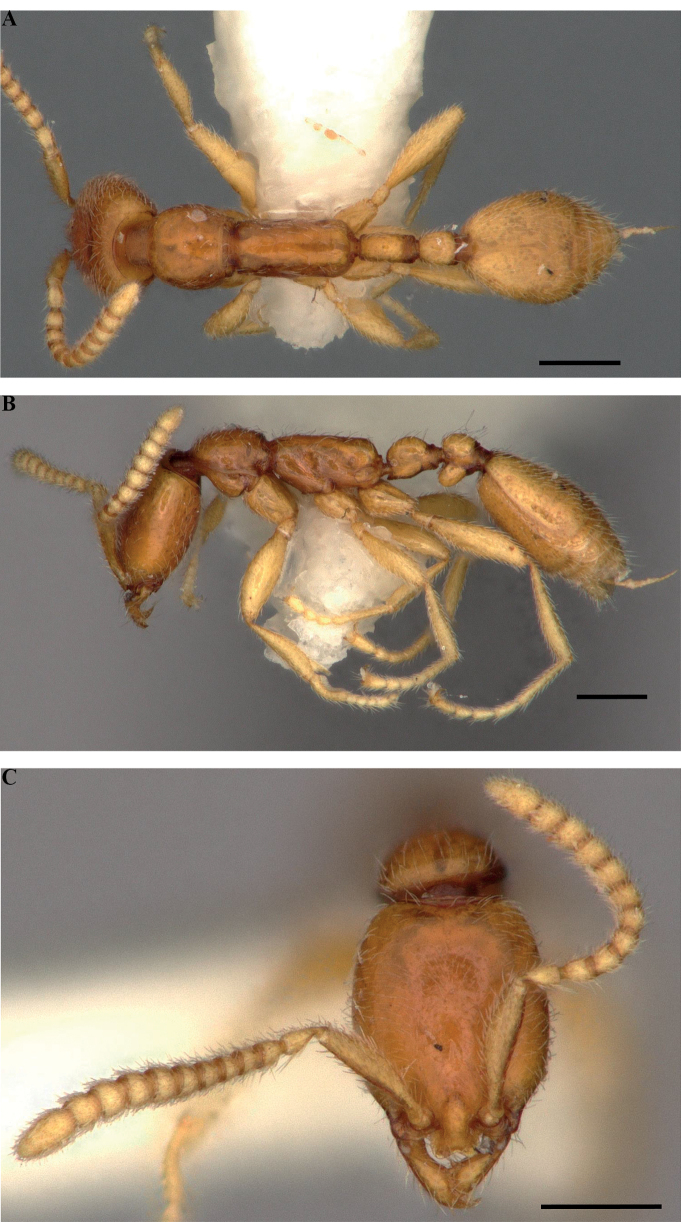
*Leptanillabelantan*, holotype (MCZ:Ent:00728278), worker **A** profile view **B** dorsal view **C** full-face view. Scale bars: 0.2 mm.

**Figure 7.**
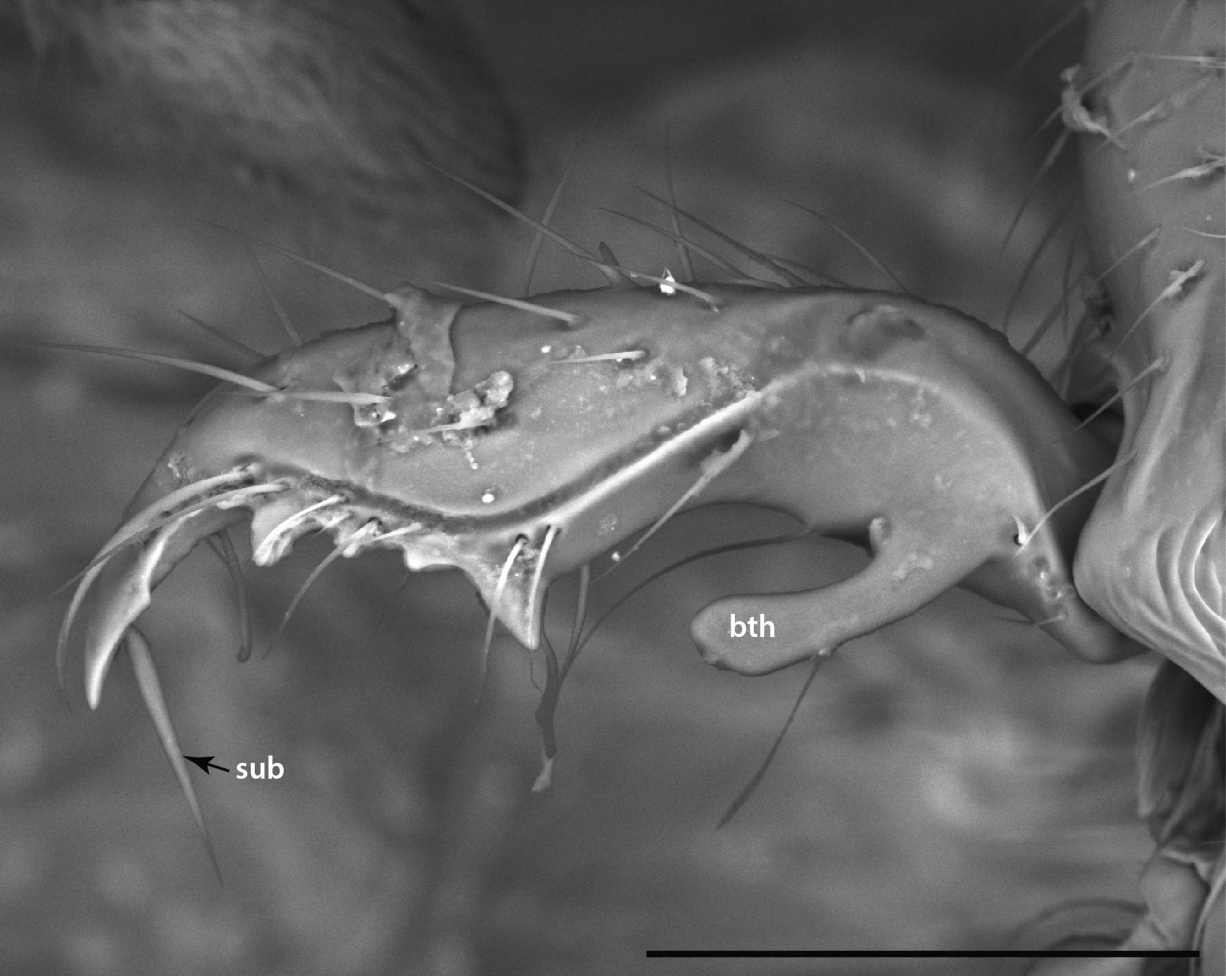
Mandible of *Leptanillabelantan* (MCZ:Ent:00728277), dorsal view, worker. Abbreviations: sub = subapical mandibular seta; bth = most proximal tooth. Scale bar: 0.1 mm.

**Figure 8.**
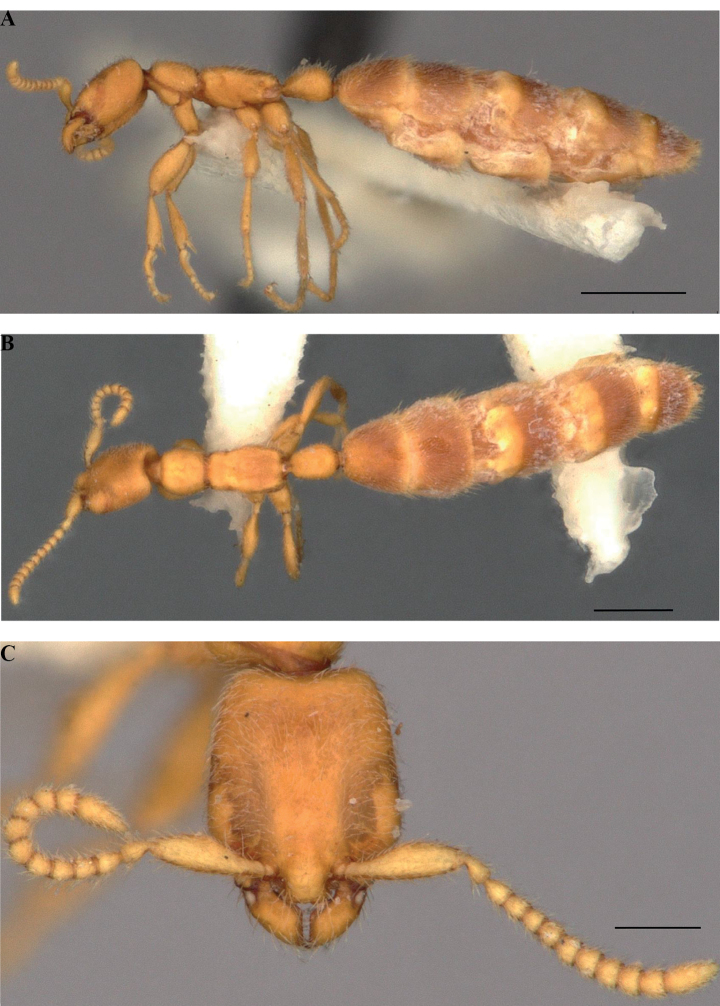
Gyne of *Leptanillabelantan* (MCZ:Ent:00728275) **A** profile view **B** dorsal view **C** full-face view. Scale bars: 0.5 mm (**A, B**); 0.2 mm (**C**).

Figs 6A–C , 7 , 8A–C

#### Type material.

***Holotype*.** Malaysia – Selangor • 1 worker; Genting Highlands, below Sri Layan; 1.iv.1981; W. L. Brown leg.; hill forest, red-rotten wood; MCZ:Ent:00728278. MCZC***Paratypes*.** Malaysia – Selangor • 1 gyne; same data as for holotype; MCZ:Ent:00728275; MCZC • 3 worker, same data as for holotype; MCZ:Ent:00728276, MCZ:Ent:00728277, MCZ:Ent:00793731; MCZC • 2 worker, same data as for holotype; MCZ:Ent:00793729, MCZ:Ent:00793730; UCDC.

#### Measurements (mm) and indices, worker.

***Holotype***: HW = 0.34; HL = 0.44; SL = 0.28; LF2 = 0.05; ML = 0.2; WL = 0.56; PrW = 0.22; MW = 0.148; PTL = 0.14; PTH = 0.13; PTW = 0.08; PPL = 0.11; PPW = 0.10; PPH = 0.16; TW4 = 0.29; CI = 77; SI = 82.38; MI = 58; PI = 59; PPI = 91; TI1 = 33. ***Paratypes*** (*n* = 5): HW = 0.33–0.35; HL = 0.42–0.45; SL = 0.24–0.28; ML = 0.18–0.21; WL = 0.54–0.57; PrW = 0.224 –0.23; MW = 0.15–0.16; PTL = 0.14–0.16; PTH = 0.11–0.13; PTW = 0.08–0.09; PPL = 0.10–0.11; PPW = 0.09–0.10; PPH = 0.15–0.16; TW4 = 0.29–0.31; CI = 75–77; SI = 74–82; MI = 52–60; PI = 55–59; PPI = 89–98; TI1 = 32–35

#### Measurements (mm) and indices, gyne.

HW = 0.47; HL = 0.56; SL = 0.29; LF2 = 0.06; ML = 0.20; PrW = 0.30; MW = 0.31; PTL = 0.30; PTH = 0.21; PTW = 0.22; CI = 84; SI = 61; MI = 43; PI = 72

#### Worker.

Lateral margins of cranium slightly convex. Occipital carina distinct. Frontoclypeal process present, delimited from cranium by lateral carinae, with posteromedian delimitation from cranium, projecting well anterior of labrum in full-face view; apex robust, broad in outline, emarginate, bordered by laminae. Mandible short relative to head. Four teeth present on mandible; two teeth proximad apical tooth acute, subequal in size, with two denticles interposed; most proximal tooth large, distally recurved, blunt, enlarged apically (Fig. 7). Large, tapering basal seta absent from mandible; subapical tapering seta present (Fig. 7). Maxillary palp 2-merous. Scape short, not reaching cranial vertex at rest, somewhat expanded towards apex. Pedicel length subequal to that of basal flagellomere. Flagellum submoniliform; antennomere 3 subequal in length to distal antennomeres; apical flagellomere 2× longer than subapical flagellomere. In dorsal view, pronotal margins strongly convex, pronotal width distinctly greater than mesonotal width. Pronotal dorsum moderately convex, slightly elevated above dorsal mesonotal vertex. Lateral margins of mesonotum and metapectal-propodeal complex subparallel in dorsal view; mesonotum not constricted anteriorly. Meso-metapleural suture entirely absent; fusion of mesonotum with propodeum marked by shallow excavation. Propodeum angular in profile view; propodeal declivity slanted; posterolateral corners rounded. Tarsomeres longer than broad. Meso- and metatibial spur formula 2b,2(1s,1p). Anterior margin of petiole linear in dorsal view. Abdominal segment II longer than wide, with distinct dorsal node; margins parallel in dorsal view; margin of abdominal sternite II linear in profile view, angled ventrally anteriorly; subpetiolar process present, not lamellate, anterior face concave in profile view. Length of abdominal segment II distinctly greater than that of III. Abdominal segment III longer than wide in dorsal view. Breadth of abdominal segment III less than half the breadth of abdominal segment IV in dorsal view (TI1 = 30–33). Anteroposterior length of abdominal tergite IV greater than that of V–VIII combined. Respective anteroposterior lengths of abdominal segments V–VII subequal. Coloration brown.

#### Gyne.

As for genus. Mandible with distinct basal and masticatory margins, edentate, not demarcated by a distinct subapical incisor; masticatory margin longer than basal margin. In dorsal view, breadth of mesonotum less than that of pronotum or metanotal-propodeal complex. Petiole longer than broad in dorsal view (PI = 0.719), constricted anteriorly along both transverse and dorsoventral axes; subpetiolar process absent. Dorsal node situated towards posterior of petiole. Abdominal segment III axial relative to posterad abdominal segments. Postsclerites of abdominal segments III–VII subequal in length. Vestiture consisting of short subdecumbent to suberect setae, longer and more abundant on gaster than on remainder of soma.

#### Etymology.

“Belantan” is Malay for a club-like weapon, in reference to the shape of the proximal tooth of the worker mandible, the apical expansion of which is unique in mandibular teeth observed in *Leptanilla*. The specific epithet is a noun in apposition and therefore invariant.

#### Remarks.

The worker of *Leptanillabelantan* is closest to that of *Leptanillajudaica* Kugler, 1987 and *Leptanillaujjalai* Saroj, Mandi & Dubey, 2022 in appearance. Like *L.ujjalai*, *L.belantan* possesses an enlarged, truncate proximal tooth on the mandible, which in the latter species is bent distally; *L.belantan* differs from *L.ujjalai* in not having a serrated subpetiolar process and in the apex of the frontoclypeal process being emarginate, rather than entire. Castaneous coloration and lack of a meso-metapleural furrow set *L.belantan* apart from *L.judaica*. The gyne habitus of *L.belantan* is nearest to *Leptanillaescheri* (Kutter, 1948), differing in the elongation of the masticatory margin and the complete absence of ommatidia.

It is quite possible that the specimens identified as *L.escheri* and mentioned by Hölldobler et al. (1989) in fact belong to this species, since these also originated in peninsular Malaysia, although this speculation is unprovable because the repository of those specimens was not reported. It is also possible but unconfirmable that the undescribed *Leptanilla* species portrayed in Bolton (1990b: figs 8–11) corresponds to *L.belantan*. As with *L.escheri*, the placement of *L.belantan* in the *Leptanillathai* species group must be regarded with some caution until this hypothesis can be tested with phylogenomic inference. It is conceivable that *L.belantan* instead belongs to the *Leptanillahavilandi* species group, since the worker caste of the two clades are at times distinguishable only by phenetic minutiae such as sculpturation. Unlike its putative close relatives within the *Leptanillathai* species group, *L.belantan* exists in parapatry with the *Leptanillahavilandi* species group, allowing for the possibility that this species belongs to the latter clade.

The mandible of the gyne of *L.belantan* differs from the falcate facies observed in all other *Leptanilla* gynes, with the masticatory margin being longer than the basal margin. The gyne mandible in *L.belantan* therefore converges with the synapomorphic condition of the Poneroformicines (Richter et al. 2022).

### ﻿ Leptanilla acherontia sp. nov.

Taxon classificationAnimaliaHymenopteraFormicidae

50843A90-54E6-5BBE-92D7-25CC227571DF

https://zoobank.org/497DDEFF-A7AA-4AFE-9C29-E7F29D2F43F2

Figs 9A–C , 10

#### Type material.

***Holotype*.** Kenya – Kakamega • 1 worker; Kakamega Forest, Isecheno; 00.24°N, 34.85°E; 6 Nov. 2002; 1550m a.s.l.; W. Okeka leg.; equatorial rainforest, sifted litter in soil under *Morusmesozygia*; CASENT0842720; UCDC***Paratype*.** Kenya – Kakamega • 1 worker; same data as for holotype; CASENT0178284; LACM.

#### Other material examined.

Kenya – Kakamega • 1 worker; same data as for holotype; CASENT0842721; UCDC.

#### Measurements (mm) and indices.

***Holotype***: HW = 0.22; HL = 0.29; ML = 0.11; SL = 0.13; WL = N/A; PrW = 0.139; MW = 0.12; PTL = 0.11; PTH = N/A; PTW = 0.10; PPW = 0.11; TW4 = 0.21; CI = 75; SI = 62; MI = 52; PPI = 128.09; TI1 = 54.81. ***Other material examined***: HW = 0.21; HL = 0.28; ML = 0.11; SL = 0.12; WL = 0.37; PrW = 0.13; MW = 0.11; PTL = 0.10; PTW = 0.09; PPL = 0.09; PPW = 0.10; TW4 = 0.20; CI = 75; SI = 58; MI = 55; PPI = 113; TI1 = 47.

#### Description.

Lateral margins of cranium subparallel. Occipital carina indistinct. Frontoclypeal process absent; frontoclypeal margin with median portion slightly raised, entire. Mandibles short relative to head. Three teeth present on mandible; apical and subapical teeth entire, intermediate tooth shallowly bifid (Fig. 10); irregular denticles interposed between all three teeth. Large, tapering basal seta absent from mandible; subapical tapering seta present. Scape short, not reaching cranial vertex at rest, somewhat expanded towards apex. Pedicel length distinctly greater than that of basal flagellomere. Flagellum submoniliform; length of basal flagellomere distinctly less than that of distal antennomeres; apical flagellomere 2× longer than subapical flagellomere. In dorsal view, pronotal margins moderately convex, pronotal width only slightly greater than mesonotal width. Pronotal dorsum planar, not elevated above dorsal mesonotal vertex. Lateral margins of mesonotum and metapectal-propodeal complex subparallel in dorsal view; mesonotum not constricted anteriorly. Meso-metapleural suture absent dorsally; pleural portion visible as sinuate signum in oblique anterior view. Propodeum convex in profile view; propodeal declivity vertical and linear; posterolateral corners of propodeum rounded. Tarsomeres broader than long. Meso- and metatibial spur formula 1b,2(1b,1p). Anterior margin of petiole linear in dorsal view. Length and breadth of abdominal segment II subequal, distinct dorsal node present; margins parallel in dorsal view; subpetiolar process absent. Lengths of abdominal segments II–III subequal. Abdominal segment III slightly broader than long in dorsal view. Breadth of abdominal segment III approximately half that of abdominal segment IV in dorsal view (TI1 = 47–54). Abdominal tergites IV–VII visible in posterodorsal view. Anteroposterior length of abdominal tergite IV twice anteroposterior length of abdominal tergite V in dorsal view. Anteroposterior lengths of abdominal tergites V–VI subequal; anteroposterior length of abdominal tergite VII much less than that of abdominal tergite VI. Sculpture largely absent. Vestiture consisting of short subdecumbent setae, longer and more abundant on gaster than on remainder of soma. Coloration yellowish.

**Figure 9.**
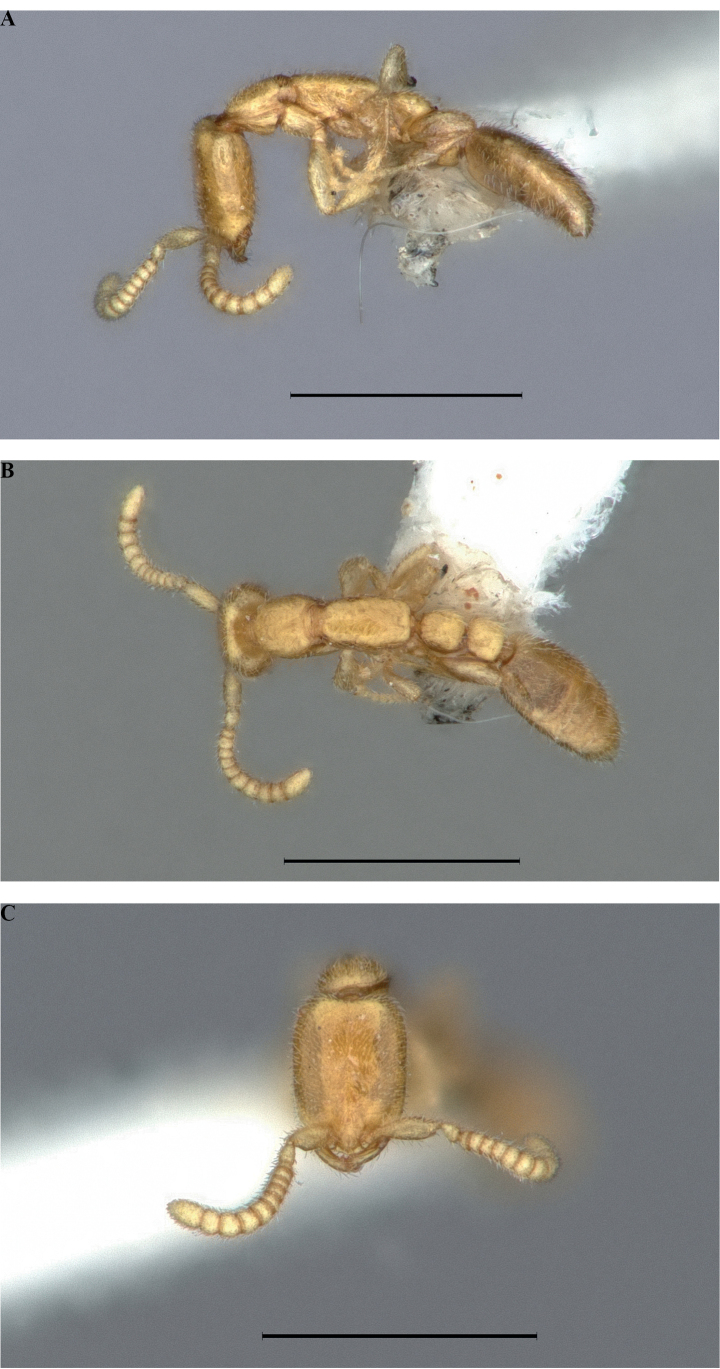
*Leptanillaacherontia*, holotype (CASENT0842720), worker **A** profile view **B** dorsal view **C** full-face view. Scale bars: 0.5 mm.

**Figure 10.**
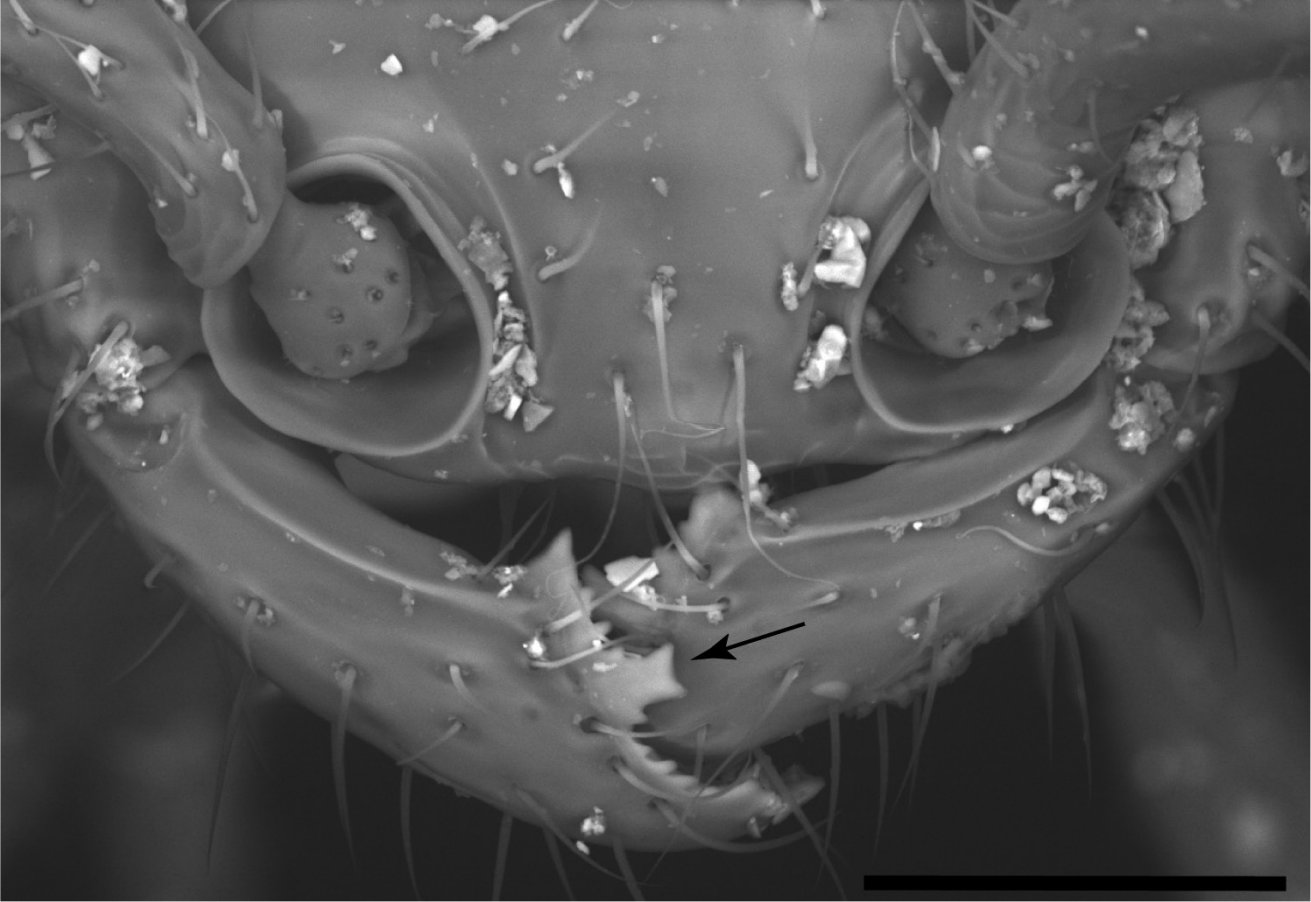
Mandibles of *Leptanillaacherontia* (CASENT0842721), dorsal view, worker. Bifid tooth marked with arrow. Scale bar: 0.05 mm.

#### Etymology.

The specific epithet refers to Acheron, a subterranean river in Greek mythology, continuing a theme established by the specific epithets of the related Iberian species *Leptanillacharonea* and *Leptanillaplutonia* López, Martínez & Barandica, 1994. The gender is feminine.

#### Remarks.

*Leptanillaacherontia* sp. nov. most closely resembles *Leptanillarevelierii* Emery, 1870, *Leptanillakubotai* Baroni Urbani, 1977, and *Leptanillaokinawensis* Terayama, 2013, with three mandibular teeth and a linear clypeal margin. Abdominal tergite V is proportionally longer in dorsal view in *L.acherontia* than *L.revelierii*, while *L.acherontia* differs from *L.kubotai* and *L.okinawensis* in pedicel shape and larger body size, respectively. Based on consultation of AntWeb images (https://www.antweb.org), *Leptanilla* UG01, known only from equatorial rainforest in Kibale National Park, Uganda, is almost certainly conspecific with *L.acherontia*.

With *Leptanillaboltoni* Baroni Urbani, *L.acherontia* is one of only two described Afrotropical *Leptanilla* species for which the worker caste is known. Phylogenomic inference indicates that *Leptanilla* zhg-ke02 may represent the male of *L.acherontia* (pers. obs.), but further sampling of sympatric *Leptanilla* would be required for this association to be decisive. The type locality of *L.acherontia* is situated in perhumid equatorial rainforest, contrasting with the semi-arid provenance of *Leptanilla* zhg-ke01 and other Afrotropical and Western Palaearctic *Leptanilla*. It is unclear to what degree climatic conditions dictate the distributions of *Leptanilla* species.

### ﻿ Leptanilla bethyloides sp. nov.

Taxon classificationAnimaliaHymenopteraFormicidae

12BA0053-DED9-5412-AFD9-D04006C3278D

https://zoobank.org/5955A34E-6467-442B-8A30-4FD9F24FCB8D

Figs 11A–C , 12

#### Type material.

***Holotype*.** China – Hong Kong • 1 male; Tai Po Kau; 22.44°N, 114.18°E (estimated from Google Earth to nearest minute), 15 Jun. 1964; W. J. Voss and W. M. Hui leg.; CASENT0842864. BPBM. ***Paratype*.** China – Hong Kong • 1 male; same locality as for preceding; 2–6 Jul. 1964; L. K. and H. W. Ming leg.; light trap; CASENT0842865. BPBM.

#### Measurements (mm) and indices, male.

***Holotype***: HW = 0.27; HL = 0.32; SL = 0.10; LF2 = 0.04; EL = 0.11; EW = 0.12; WL = 0.59; MSL = 0.35; MSW = 0.23; PTW = 0.25; PTL = 0.10; PTH = 0.13; REL = 34; SI = 36; CI = 244; OI = 113; MSI = 152.38; PI = 247.52. ***Paratype***: HW = 0.25; HL = 0.30; SL = 0.08; LF2 = 0.04; EL = 0.11; EW = 0.12; WL = 0.53; MSL = 0.31; MSW = 0.22; PTH = 0.12; REL = 35; SI = 32; CI = 219; OI = 110; MSI = 139

#### Description.

Cranial outline quadrate. Occiput emarginate in full-face view. Frons not produced into anterior shelf. Mandible articulated to gena; broader than long. Mandalus large, covering entire anterodorsal mandibular surface. Maxillary palp 1-merous. Clypeus anteroposteriorly reduced, not discernible in full-face view. Anterior tentorial pits not discernible. Compound eyes wider than long in profile view (OI = 110–112), posterior margin slightly emarginate, all other margins convex. Anteromedian ocellus and compound eyes not intersecting line drawn perpendicular to anteroposterior axis of cranium. Scape anteroposteriorly compressed, longer than wide (SL = 0.081–0.095 mm), shorter than anteroposterior length of compound eye; pedicel short, subcylindrical, lateral margins parallel, length 0.5× that of scape; antennomere 3 short (LF2 = 0.037–0.039 mm), subcylindrical, length subequal to that of pedicel; flagellum submoniliform, not extending posterior to mesoscutum if folded flat over mesosoma. Pronotum and mesoscutum posteriorly prolonged. In profile view anterodorsal pronotal face diagonal to craniocaudal axis at ~45° angle, but profile of pronotum otherwise obscured by vestiture. Mesoscutal dorsum slightly convex; mesoscutum longer than broad (MSI = 139–152). Antero-admedian signum absent. Notauli absent. Parapsidal signa present, impressed. Mesoscutellum longer than tall, dorsum not lower than that of mesoscutum, posterodorsal mesoscutellar face convex, posteriorly produced, not recurved. Oblique mesopleural sulcus present, not intersecting metapectal-propodeal complex. Metapleuron distinct, transected by transverse sulcus. Metapleural gland absent. Propodeum convex in profile view, without distinct dorsal and posterior faces. Pro- and metacoxa subequal in length, metacoxa somewhat more massive; mesocoxa shorter than pro- and metacoxa. Protrochanters sphenoid in outline, distally truncate. Profemur not markedly constricted at base, anteroposteriorly compressed, incrassate; acute distal flange on posterior surface absent; arcuate medial carina absent. Protibial and profemoral length subequal; protibia not dorsoventrally compressed, without ventromedian carina; protibial comb absent; probasitarsal seta not hypertrophied. Meso- and metatibial spur formula 2b,2(1b,1p). C and Sc+R+Rs fused, tubular; *2s-rs*+R+4-6 and M+Cu tubular; all other venation absent. Costal infuscation absent. Abdominal segment II anteroposteriorly compressed, broader than long in dorsal view excluding presclerites; dorsal node present, well-developed; with median dorsal excavation. Abdominal sternite II without process, planar in profile view. Presclerites of abdominal segments IV–VIII inconspicuous. Abdominal segments III–VII without tergosternal fusion. Tergosternal fusion of abdominal segment VIII–IX unknown. Abdominal tergites III–VIII not anteroposteriorly compressed, lateral margins subparallel; breadth of abdominal tergite VIII subequal to that of abdominal tergite VII in posterodorsal view. Abdominal sternite VIII anteroposteriorly compressed, visible without dissection, posterior margin entire. Abdominal sternite IX not visible without dissection. Mulceators absent. Gonopodites articulate. Gonocoxites without complete dorsomedian and ventromedian fusion; ventromedial margin of gonocoxite with lamina; apicoventral laminae absent. Gonostylus present, outline lanceolate, apex entire. Volsellae absent. Penial sclerites dorsoventrally compressed, not basally recurved, ventromedian carina extending along most of length, without lateral laminate margins. Phallotreme dorsal, concealed by gonostyli in available specimens. Somal sclerites with thick vestiture of decumbent to suberect setae, sparsest on meso- and metapleuron; setae appressed to decumbent on antennae and legs; gonostyli with similar vestiture to abdominal postsclerites, genitalia otherwise glabrous. Base of forewing costa bearing row of exceptionally long, suberect setae. Cuticle bearing piligerous punctae; sculpture otherwise absent.

**Figure 11.**
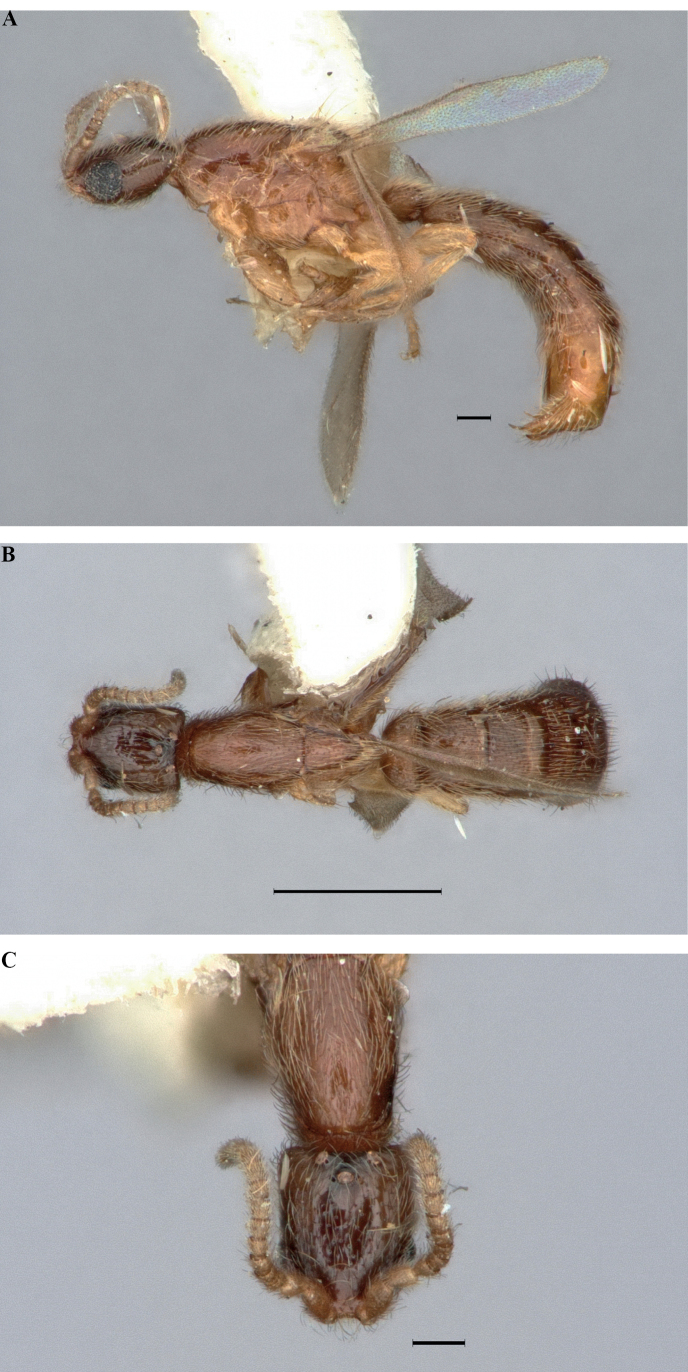
*Leptanillabethyloides*, holotype (CASENT0842864), male **A** profile view **B** dorsal view **C** full-face view. Scale bars: 0.1 mm (**A, C**); 0.5 mm (**B**).

#### Etymology.

The specific epithet refers to the gestalt of this ant, which resembles that of the flat wasps (Chrysidoidea: Bethylidae). While superficial, this resemblance was pronounced enough that the holotype and paratype of *L.bethyloides* were initially mis-sorted to Bethylidae incertae sedis at the Bishop Museum. The specific epithet is neuter.

**Figure 12.**
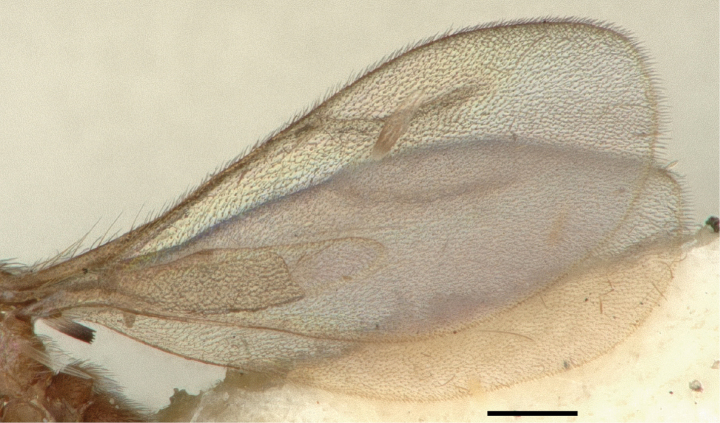
Wings of *Leptanillabethyloides* (CASENT0842865), male. Scale bar: 0.2 mm.

#### Remarks.

Among the *Leptanillabethyloides* species group, of which this is the only described species, *L.bethyloides* most closely resembles multiple undescribed morphospecies from southern Burma, differing in larger size (WL = 0.532–0.594 mm) and the proportions of the metasomal segments. Describing a new species of *Leptanilla* based solely upon male specimens, as here done for *L.bethyloides*, was eloquently argued against by Bolton (1990b), since it exacerbates the probable redundancy that plagues the taxonomy of *Leptanilla*. This description of *L.bethyloides* is justified only to give a formal species group name (i.e., the *Leptanillabethyloides* species group) to a major clade of *Leptanilla* known only from male specimens.

The volsellae are known to be wholly lacking in *Leptanilla* zhg-mm03 (Griebenow et al. in press), which shows very close morphological affinity to *L.bethyloides*; therefore, I infer the absence of the volsellae in this species. The condition of the volsellae cannot be assessed in any other representatives of the *Leptanillabethyloides* species group besides *Leptanilla* zhg-mm03. Given the relative lack of phylogenetic signal in the worker phenotype of *Leptanilla* and the scarcity of species in which the worker caste and phylogenetic position are both known, it is difficult to predict the morphology of the unknown worker of *L.bethyloides* or other members of the *Leptanillabethyloides* species group, beyond a probable 1,1 palpal formula. It is conceivable that *Leptanillamacauensis* Leong, Yamane & Guénard, 2018 represents this worker, although unlikely, given the conformity of *L.macauensis* to the worker diagnosis for the *Leptanillarevelierii* species group, where it is placed in this study.

### ﻿ Leptanilla najaphalla sp. nov.

Taxon classificationAnimaliaHymenopteraFormicidae

7E2F2F45-CD99-5AF9-92E2-879B7D26E448

https://zoobank.org/C6B1D1A1-5138-4E52-9A50-FD7054D31187

**Figure 13.**
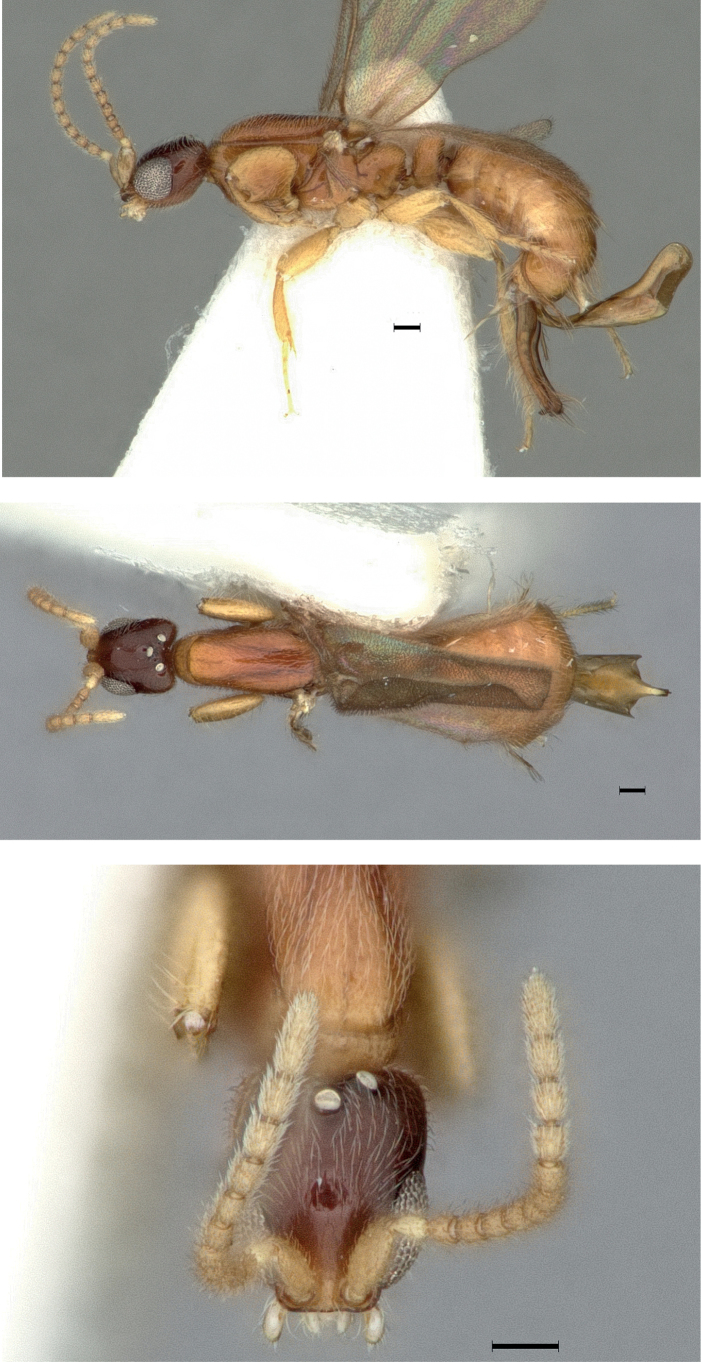
*Leptanillanajaphalla*, holotype (CASENT0106427), male **A** profile view **B** dorsal view **C** full-face view. Scale bars: 0.5 mm (**A, B**); 0.2 mm (**C**).

**Figure 14.**
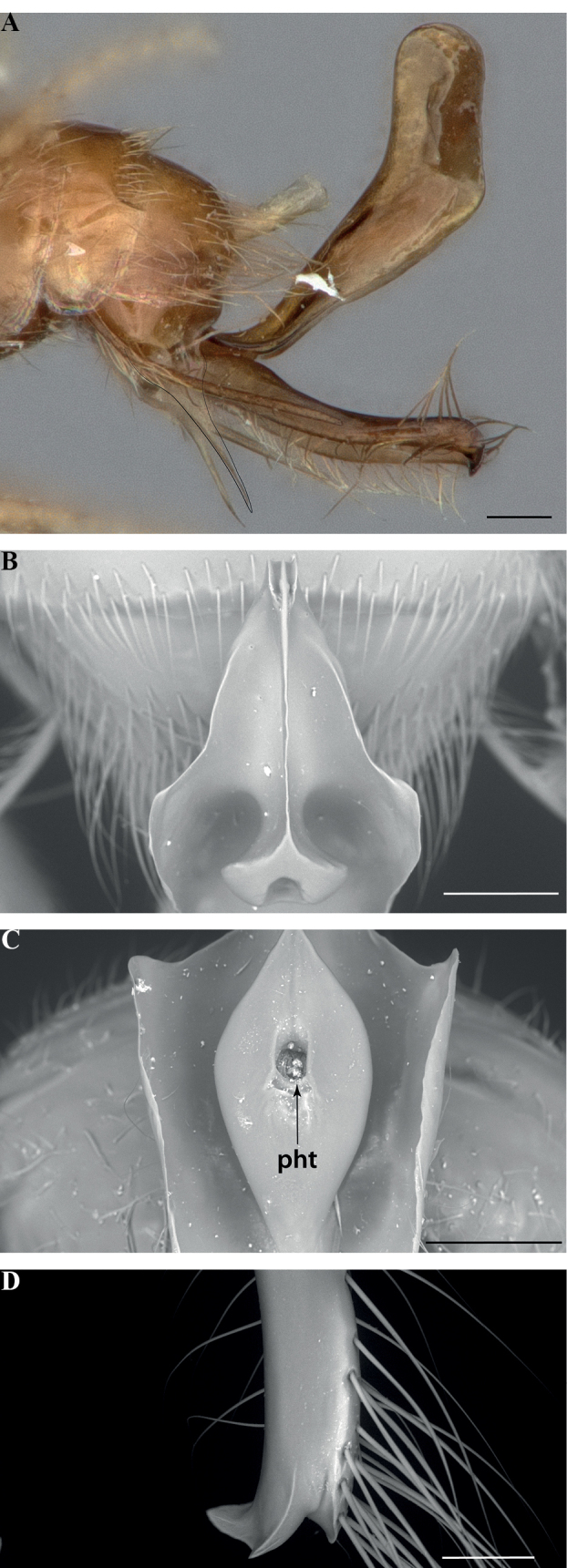
Male genitalia of *Leptanillanajaphalla***A** profile view, apicolateral gonocoxital lamina outlined (CASENT0106424) **B** penial apex, posteroventral view (CASENT0106421) **C** penial sclerites and phallotreme, ventral view (CASENT0106433) **D** volsellar apex, dorsal view (CASENT0106421). Abbreviation: pht = phallotreme. Scale bars: 0.1 mm (**A, C, D**); 0.2 mm (**B**).

**Figure 15.**
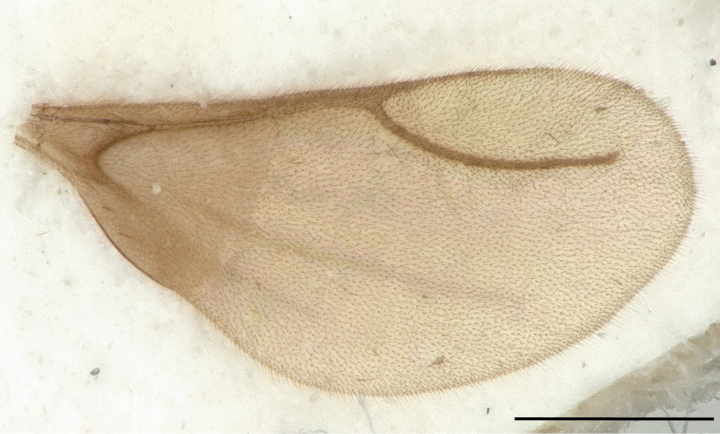
Forewing of *Leptanillanajaphalla* (CASENT0106419), male. Scale bar: 0.5 mm.

**Figure 16.**
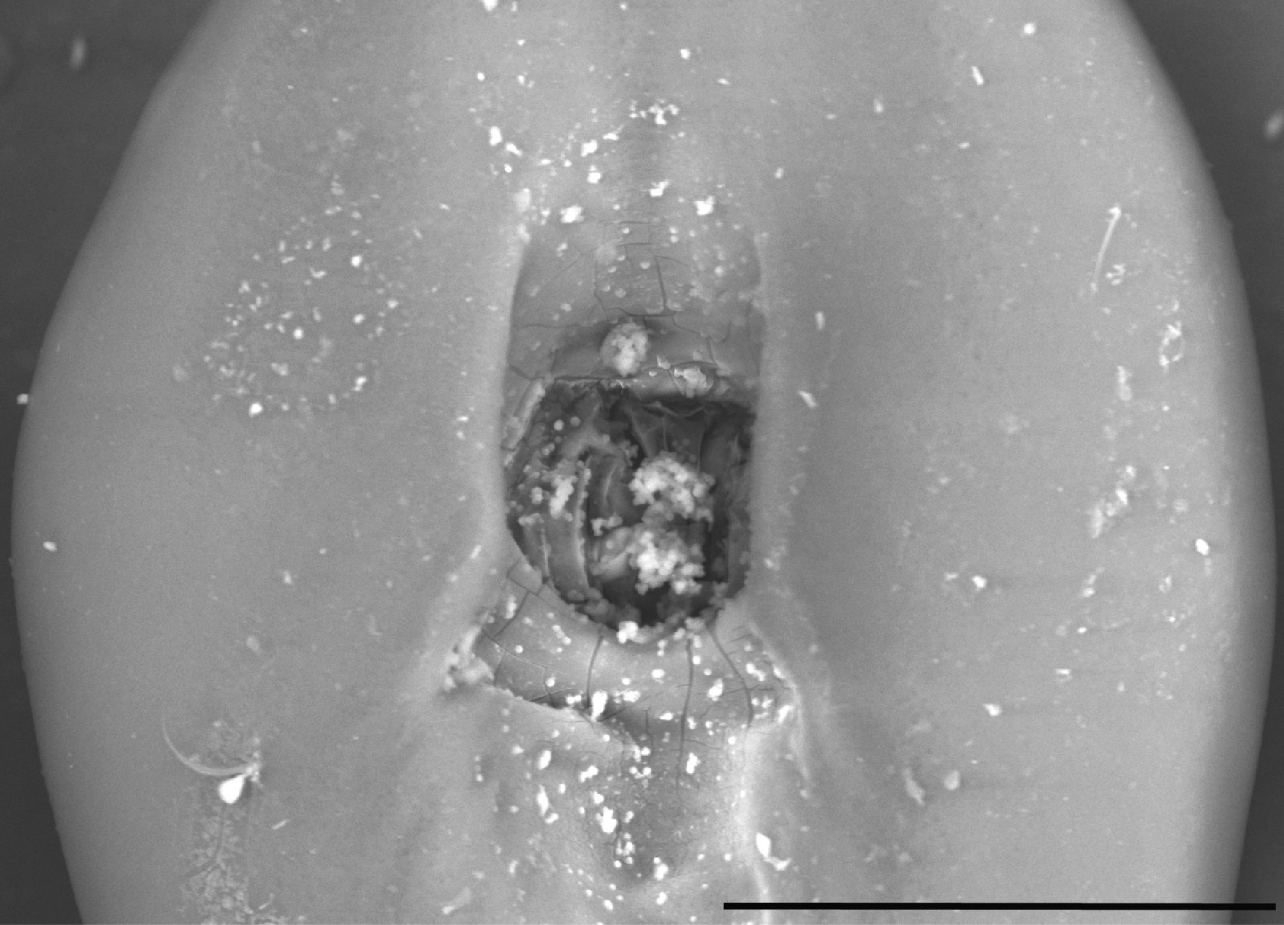
Phallotreme of *Leptanillanajaphalla* (CASENT0106433). Scale bar: 0.5 mm.

Figs 13A–C , 14A–D , 15 , 16

#### Type material.

***Holotype*.** Malaysia – Sabah • 1 male; Sipitang Dist., Mendolong; 4.917°N, 115.767°E (estimated from Google Earth to nearest minute); 27 Apr. 1988; S. Adebratt leg.; A1L; CASENT0106427 (MZLU00174197); MZLU. ***Paratypes*.** 5 male; same locality as for preceding; 16 Apr. 1988; S. Adebratt leg.; A1L; CASENT0106416 (MZLU00174186), CASENT0106417 (MZLU00174187), CASENT0106438 (MZLU00174208), CASENT0106444 (MZLU00174214), CASENT0106457 (MZLU00174227); MZLU • 5 male; same locality as for preceding; 19 Apr. 1988; S. Adebratt leg.; W5L; CASENT0106421, CASENT0106432, CASENT0106433, CASENT0106449, CASENT0106450; UCDC • 2 male; same locality as for preceding; 7 Apr. 1988; S. Adebratt leg.; A1L; CASENT0106435 (MZLU00174205), CASENT0106437 (MZLU00174207); MZLU • 1 male; same locality as for preceding; 4 May 1988; S. Adebratt leg.; T4/R; CASENT0106412; MCZC • 2 male; same locality as for preceding; 5 May 1988; S. Adebratt leg.; A1L; CASENT0106418, CASENT0106453; MCZC • 3 male; MALAYSIA, Sabah: same locality as for preceding; 13 May 1988; T4/R; CASENT0106414, CASENT0106415, CASENT0106429; CAS.

#### Measurements (mm) and indices, male.

***Holotype***: HW = 0.29; HL = 0.35; SL = 0.14; LF2 = 0.05; LF2 = 0.05; EL = 0.16; EW = 0.16; WL = 0.80; MSW = 0.26; MSL = 0.48; PTW = N/A; PTL = N/A; PTH = 0.24; REL = 46; SI = 48; CI = 82; OI = 98; MSI = 54. ***Paratypes*** (*n* = 18): HW = 0.27–0.31; HL = 0.27–0.40; SL = 0.12–0.16; LF2 = 0.05–0.06; EL = 0.14–0.17; EW = 0.14–0.16; WL = 0.69–0.83; MSW = 0.22–0.27; MSL = 0.42–0.53; PTW = 0.15–0.18; PTL = 0.12–0.15; PTH = 0.23–0.28; REL = 40–57; SI = 45–55; CI = 74–103; OI = 82–103; MSI = 48–54; PI = 105–140.

#### Description.

Cranial outline quadrate. Occiput emarginate in full-face view. Frons produced into anterior shelf. Mandible articulated to gena; distinctly longer than broad. Mandalus large, covering most of anterodorsal mandibular surface. Maxillary palp 1-merous. Clypeus anteroposteriorly reduced, concealed by frontal shelf in full-face view. Anterior tentorial pits not discernible. Compound eyes somewhat longer than wide in profile view, or EW and EL subequal (OI = 82–102), posterior margin slightly emarginate, all other margins convex. Anteromedian ocellus and compound eyes not intersecting line drawn perpendicular to anteroposterior axis of cranium. Scape anteroposteriorly compressed, longer than wide (SL = 0.124–0.154), shorter than anteroposterior length of compound eye; pedicel short, subcylindrical, lateral margins parallel, length 0.5 that of scape; antennomere 3 short, subcylindrical, length less than that of pedicel or scape; flagellum submoniliform, not extending posterior to mesoscutellum if folded flat over mesosoma. Pronotum and mesoscutum posteriorly prolonged. In profile view anterodorsal pronotal face slightly convex, diagonal to craniocaudal axis at ~ 45° angle. Mesoscutal dorsum planar; mesoscutum longer than broad (MSI = 48–53). Antero-admedian signum absent. Notauli absent. Parapsidal signa present, not impressed. Mesoscutellum longer than tall, dorsum not lower than that of mesoscutum, posterodorsal mesoscutellar face convex, not posteriorly produced. Oblique mesopleural sulcus present, not intersecting metapectal-propodeal complex. Metapleuron indistinct. Metapleural gland absent. Propodeum convex in profile view, with distinct dorsal and posterior faces; areas of these faces subequal. Procoxa longer than meso- and metacoxa; procoxa without distal transverse carina. Protrochanters sphenoid in outline, distally truncate. Profemur markedly constricted at base, anteroposteriorly compressed, incrassate; acute distal flange on posterior surface present; arcuate medial carina absent. Protibia > 0.5× length of profemur, not dorsoventrally compressed, without ventromedian carina; protibial comb present, length of processes decreasing distally; probasitarsal seta not hypertrophied. Meso- and metatibial spur formula 2b,2b. C, Sc+R+Rs, *2s-rs*+R+4-6, Rf, Mf1, *cu-a*, and Cuf+1A tubular; M+Cu and 1A nebulous; all other venation absent. Cuf+1A spectral apically, not reaching anal margin. Costal infuscation present proximal to *2s-rs*+R+4-6; C extending well beyond infuscation. Abdominal segment II anteroposteriorly compressed, slightly broader than long in dorsal view (PI = 105–133); dorsal node present, well-developed, without median excavation. Abdominal sternite II with process along posterior half of length, outline cuneiform in profile view, apex rounded. Presclerites of abdominal segments IV–VIII inconspicuous. Abdominal segments III–IX without tergosternal fusion (Griebenow et al. in press). Abdominal tergites IV–VII each broader than preceding tergite in dorsal view, lateral margins diverging posteriorly; breadth of abdominal tergite VIII less than that of abdominal tergite VII in posterodorsal view. Abdominal sternite VIII anteroposteriorly compressed, not visible without dissection, posterior margin entire (Griebenow et al. in press). Abdominal sternite IX with posteromedian fusion to gonocoxites (Griebenow et al. in press); anteroposteriorly compressed along median axis, laterally expanded and lobate. Mulceators present, subcircular in cross-section, longer than anteroposterior length of gonocoxites. Gonocoxites bulbous, with complete dorsomedian and ventromedian fusion; apicoventral laminae present, subulate in outline. Gonostyli absent. Volsellae present, with complete proximomedian fusion, subcircular in cross-section; sclerotized medial carina present at volsellar apex, produced into pair of denticles, dorsal denticle shorter than ventral one. Penial sclerites not dorsoventrally compressed, basally recurved, proximal ¼ subcircular in cross-section, apical 1/3 with ventromedian carina; rounded platform proximad this median carina with outline elliptical; phallotreme subapical and ventral, recessed, not surrounded by vestiture of setae; lateral laminate flanges present. Most sclerites with vestiture of subdecumbent to appressed setae; elongated on posterior margins of abdominal tergites III–VIII, increasing in length posteriorly; anterior faces of mulceators with elongate suberect setae; ectal faces of volsellae with suberect to erect setae, genitalia otherwise bare. Cuticle bearing piligerous punctae; sculpture fatiscent distad and proximad phallotreme (Fig. 16).

#### Etymology.

The specific epithet derives from *Naja* (Squamata: Elapidae), the cobra, and -*phalla*, meaning penis. This refers to the florid facies of the penial sclerites, which recalls the threat display of these snakes: the dorsal curvature of the penial sclerites resembles the rearing posture, while the lateral laminae resemble the extended “hood” of the cobra. The specific epithet is feminine.

#### Remarks.

The males of *L.najaphalla* uniformly differ from the sympatric undescribed morphospecies *Leptanilla* zhg-my05, to which *L.najaphalla* is sister, in the outline of the apicolateral gonocoxital lamina and the proportions of the penial sclerites and volsellae to the gonocoxites.

The description of *L.najaphalla* only from male specimens is justified for the same reasons as provided for the description of *L.bethyloides*, also only from male specimens (see “Remarks” concerning *L.bethyloides* above): the clade to which this species belongs, heretofore referred to as the “Bornean morphospecies group”, is known only from male specimens. *Leptanillanajaphalla* was included in the phylogenetic analyses of Griebenow (2020, 2021) under the provisional identifier *Leptanilla* zhg-my02, with the genitalia being the subject of detailed morphological study using micro-computed tomography (Griebenow et al. in press) under that same provisional identifier.

##### ﻿Revised diagnosis and generic classification of Leptanillinae

Based upon total-evidence and phylogenomic inference (in preparation by the author) corroborated by previous studies (Griebenow 2020, 2021), I here enact a revised classification of the Leptanillinae, reducing the number of genera to three. Summaries of character states that in combination differentiate major clades of the Leptanillinae from their relatives are provided below. These summary diagnoses are based upon all adult castes and larvae, when available. Apomorphies relative to the parent taxon are italicized; characters of uncertain polarity are marked with an asterisk.

### ﻿ Leptanillinae

Taxon classificationAnimaliaHymenopteraFormicidae

Emery, 1910

FF818E27-A13E-582C-9003-A6BC077EF6BC

#### Type genus.

*Leptanilla* Emery, 1870: 196.

#### Worker diagnosis

**Figure 17.**
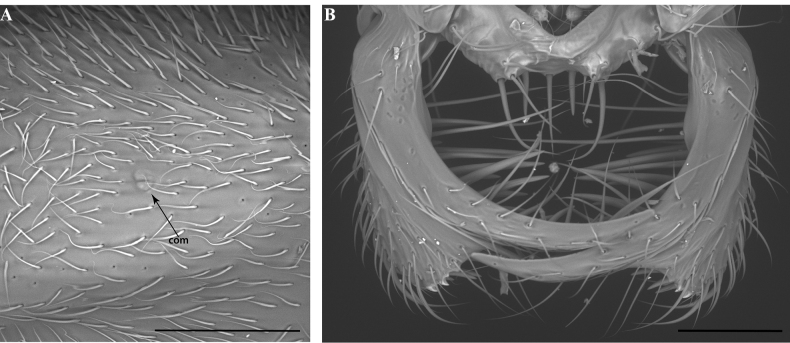
Aspects of *Protanillaizanagi*, worker **A** profile view of posterior half of cranium **B** ventral view of the mandibles. Abbreviation: com = compound eye. Scale bars: 0.1 mm (**A**); 0.2 mm (**B**).

**Figure 18.**
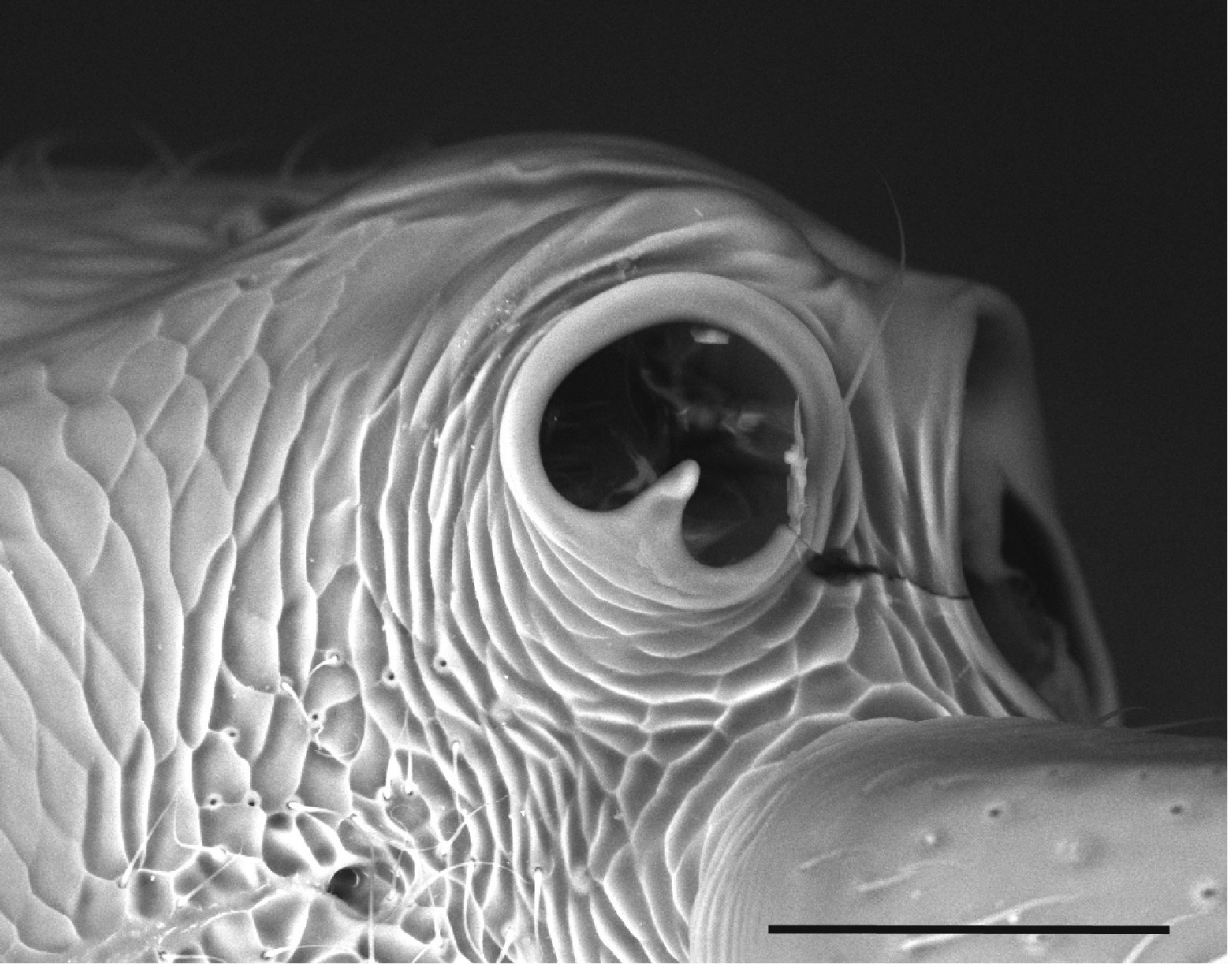
Metacoxal foramen of *Leptanillahavilandi* (CASENT0010809), ventral view, worker. Scale bar: 0.05 mm.

(modified from Bolton 2003):

1. Mandibles without differentiated basal and masticatory margins.
2. At least one preapical tooth or lobe present on mandible.
3. Frontal lobes absent.
4. Antennal sockets dorsal, fully exposed.
5. Compound eyes absent, if present (*Protanillaizanagi* Terayama, 2013) then reduced to two ommatidia (Fig. 17A).
6. Ocelli absent.
7. Antenna 12-merous.
8. Promesonotal suture fully articulated.
9. Propodeal lobes weakly present (Opamyrmini) or absent (Leptanillini).
10. Propodeal spiracle situated low on propodeum.
11. Metacoxal foramen small, fully closed (Fig. 18).
12. Suture absent from annulus surrounding metacoxal foramen.
13. Metapleural gland present.
14. Orifice of metapleural gland covered by dorsal cuticular flange.
15. Helcial sternite reduced and partly covered by corresponding tergite.
16. Spiracle of abdominal segment III large and placed far forward.
17. Spiracles of abdominal segments IV–VII concealed by posterior margins of preceding tergites.
18. Petiole sessile, rarely subsessile (*Protanillataylori* species group).
19. Abdominal postsclerites II with (Leptanillini) or without (Opamyrmini) complete tergosternal fusion.
20. Abdominal postsclerites III with (Leptanillini) or without (Opamyrmini) tergosternal fusion.
21. Abdominal segment III petiolate (Leptanillini) or not (Opamyrmini).
22. Abdominal segment IV without tergosternal fusion.
23. Stridulitrum absent from abdominal segment IV.
24. Abdominal tergite VII large, with simple posterior margin.
25. Sting present.
26. Pretarsal claws edentate.

#### Gyne diagnosis.

As above, but alate or dichthadiiform (rarely ergatoid). If alate then with ocelli and pterostigma; hindwing with R + Rs and 1A tubular, not intersecting distal wing margin. If dichthadiiform then compound eyes reduced to one or two ommatidia, or absent; ocelli absent; mandibles sometimes edentate.

#### Male diagnosis

**Figure 19.**
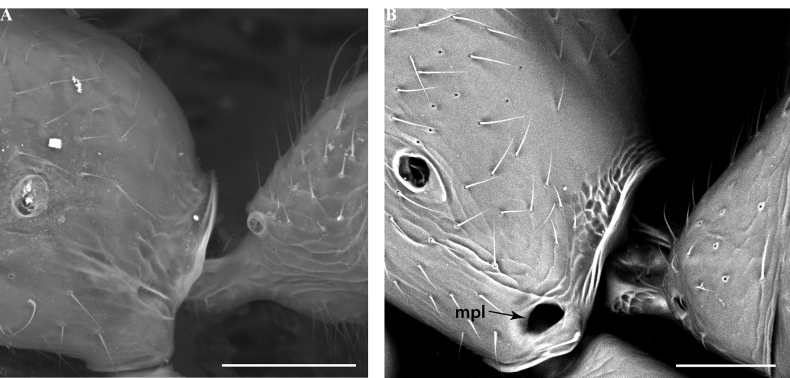
Metapleuron in male Leptanillinae**A**Leptanillanr.indica (CASENT0106381) **B***Leptanilla* zhg-th02 (CASENT0842615). Abbreviation: mpl = metapleural gland orifice. Scale bars: 0.05 mm.

(modified from Boudinot 2015):

1. Mandible edentate, nub-like or spatulate (*Leptanillaanomala* (Brues, 1925), comb. nov.).
2. Frontal carinae absent.
3. Cuticular pegs absent from anterior clypeal margin.
4. Antenna 13-merous.
5. Funiculus filiform to submoniliform.
6. Oblique mesopleural sulcus present or absent.
7. Metapleural spiracular plate absent.
8. Propodeal lobes inconspicuous or absent.
9. Metacoxal foramen small, fully closed.
10. Mesotibia with one or two spurs or none.
11. Metatibia with one or two spurs.
12. Metatarsus lacking posterolateral line of dense differentiated setae.
13. Pretarsal claws edentate.
14. Pterostigma present or absent.
15. Rs+M absent (Leptanillini) or present, nebulous (Opamyrmini).
16. *1m-cu* absent (Leptanillini) or present, nebulous (Opamyrmini).
17. Jugal lobe absent.
18. Hindwing venation reduced, at most R+Rs and 1A tubular.
19. Metapleural gland absent (Fig. 19A) or rarely present (Fig. 19B) (e.g., *Leptanilla* zhg-th02).
20. Petiole present or reduced to absent (*Leptanillathai* species group, *Leptanillahavilandi* species group).
21. Helcium axial or infra-axial.
22. Abdominal segment III not petiolate, or rarely petiolate (*Protanillabicolor* species group).
23. Abdominal segment IV not vaulted, as long as, or distinctly longer than posterad abdominal segments.
24. Abdominal spiracles IV–VIII obscured by preceding tergites.
25. Posterior margin of abdominal sternite IX with posteromedian process, or entire, or emarginate, or with mulceators.
26. Cerci absent.

#### Larval diagnosis.

Stenocephalous, with post-cranial soma moderately (i.e., habitus pogonomyrmecoid) to extremely (i.e., habitus leptanilloid) elongate. Mandibles typhlomyrmecoid or leptanilloid.

### ﻿ Opamyrmini

Taxon classificationAnimaliaHymenopteraFormicidae

Boudinot & Griebenow, tribe nov.

FF1EA791-AB27-538E-B2BA-657B5826A895

https://zoobank.org/B3CFA4FF-FECD-42E8-B7CB-814A16C23659

**Figure 20.**
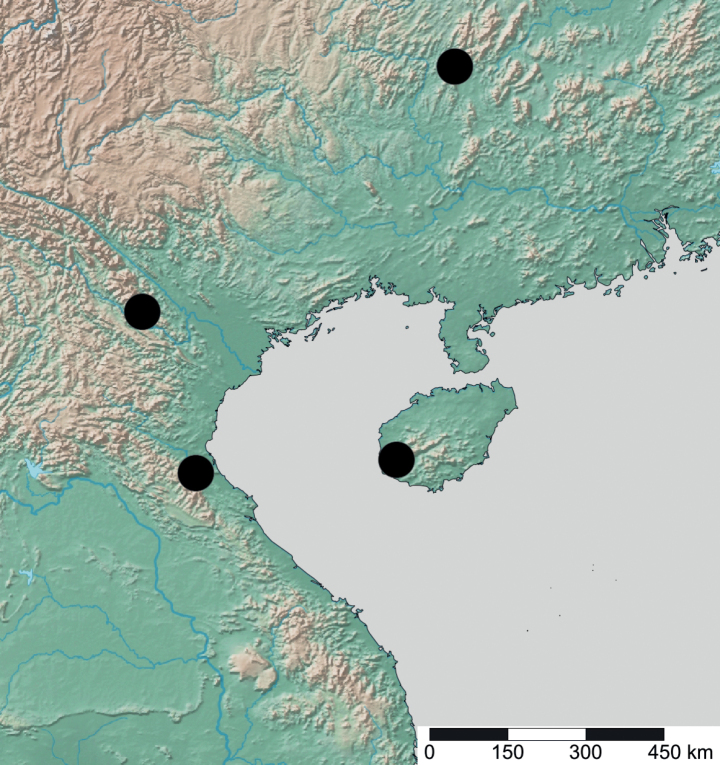
Geographical range of *Opamyrma*. Locality information derived from AntWeb and available literature, visualized with SimpleMappr.

Opamyrma Yamane, Bui & Eguchi, 2008 (Fig. 20).

#### Worker diagnosis.

1. Medial mandibular surface with single peg-like chaeta.
2. Mandible with one tooth and several preapical lobes.
3. Labrum with multiple ranks of peg-like chaetae (Yamada et al. 2020: fig. 2F).
4. Maxillary palp 4-merous.
5. Labial palp 2-merous.
6. Clypeus extending posteriorly between antennal toruli.
7. Posteromedian epistomal sulcus not clearly discernible.
8. Occiput visible in full-face view.
9. Meso-metapleural suture absent.
10. Propodeal lobe weakly present.
11. Subpetiolar process absent.
12. Abdominal postsclerites II without tergosternal fusion.
13. Abdominal segment III not petiolate or narrower than posterad abdominal segments.
14. Abdominal postsclerites IV subequal in length to abdominal postsclerites V and VI.
15. Abdominal tergite VII hypertrophied, dome-like.

#### Gyne diagnosis.

As above, but alate, with compound eyes and three ocelli; occipital carina with short medioventral interruption. M + Cu complete, tubular; *cu-a* present; Rs + M, Cuf2 and -3, and *1m-cu* present and spectral; *2r-rs* + Rsf4 adjoined by Rsf3.

#### Male diagnosis.

As for the Leptanillinae, but Rs+M and *1m-cu* present, and abdominal segment II without tergosternal fusion. Cupula non-annular. Lateropenite present, fully articulated to parossiculus, and malleate.

#### Larval diagnosis.

Habitus pogonomyrmecoid. Cranium subelliptical in full-face view. Mandibles typhlomyrmecoid, without teeth, lateral surfaces smooth. Setae short, suberect. Ventral prothoracic process and hemolymph tap on abdominal segment IV absent.

### ﻿ Opamyrma

Taxon classificationAnimaliaHymenopteraFormicidae

Yamane, Bui & Eguchi, 2008

831D83F1-193F-507E-9097-08E66E9C0380

Opamyrma Yamane, Bui & Eguchi, 2008: 56. Type species: Opamyrmahungvuong Yamane et al., by monotypy.
Opamyrma hungvuong Yamane, Bui & Eguchi, 2008.

#### Diagnosis.

As for tribe.

#### Remarks.

*Opamyrma* was described in the Amblyoponinae, based solely upon worker morphology (Yamane et al. 2008), and was subsequently found by Ward and Fisher (2016) to belong to the Leptanillinae based upon phylogenetic inference from 11 nuclear loci. All subsequent phylogenetic inference consistently recovers *Opamyrma* as sister to the remaining Leptanillinae (Borowiec et al. 2019; Griebenow 2020, pers. obs.). All adult forms lack complete tergosternal fusion in abdominal segment II, a plesiomorphy unique among the Leptanillinae. The presence of weak propodeal lobes (Yamada et al. 2020: 34) is plesiomorphic relative to the Leptanillini, in which the propodeal lobes are absent in the worker caste. The lack of petiolation of abdominal segment III in the worker caste of *Opamyrma* is also unique among the Leptanillinae but this character state may not be plesiomorphic for the subfamily. The polarity of the proportions of abdominal postsclerites IV relative to V–VI within the Leptanillinae is also unclear.

### ﻿ Leptanillini

Taxon classificationAnimaliaHymenopteraFormicidae

Emery, 1910

23DF9092-0123-5976-9ECD-110567BE18D0

Leptanilla Emery, 1870.
Protanilla Taylor in Bolton, 1990b.

#### Worker diagnosis.

**Figure 21.**
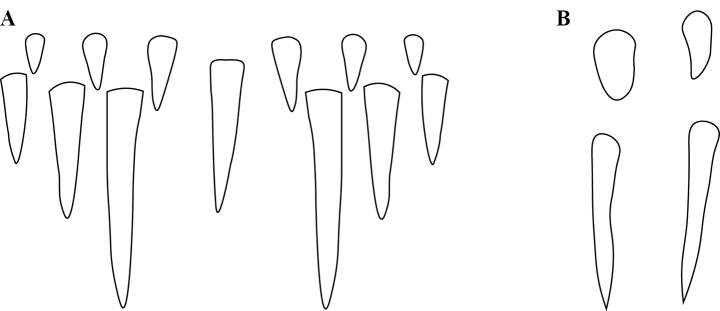
Labral chaetae in *Protanilla*, diagrammatic anterior view **A***Protanilla* id01, gyne **B***Protanillawallacei* (CASENT0842699), worker.

**Figure 22.**
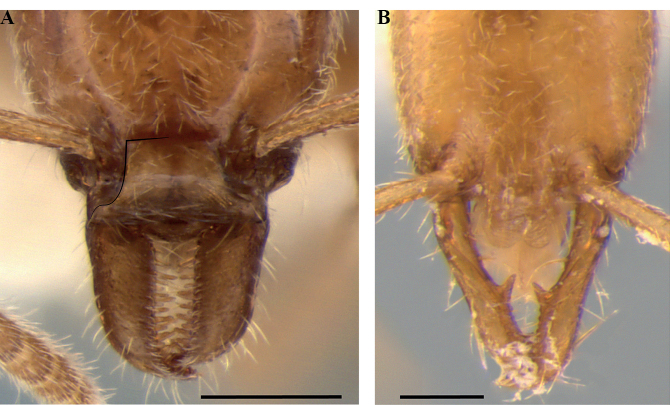
Condition of the worker frontoclypeal margin in *Protanilla* (**A**) and *Leptanilla* (**B**) **A***Protanillabeijingensis* (CASENT0842639) **B***Leptanillalaventa* (CASENT0842746). Scale bars: 0.5 mm (**A**); 0.1 mm (**B**).

1. Medial mandibular surface with or without peg-like chaetae.
2. Mandible with 0–4 teeth along medial margin.
3. Labrum with (Fig. 21A, B) or without multiple ranks of peg- or pencil-like chaetae.
4. Maxillary palp 4-, 2-, or 1-merous.
5. Labial palp 2- or 1-merous.
6. Clypeus extending posteriorly between antennal toruli (Fig. 22A) or not (Fig. 22B).
7. Posteromedian epistomal sulcus clearly discernible (Fig. 22A) or not (Fig. 22B).
8. Occiput not visible in full-face view.
9. Meso-metapleural suture present or absent.
10. Propodeal lobes absent.
11. Subpetiolar process present or absent.
12. Abdominal postsclerites II–III with tergosternal fusion.
13. Abdominal segment III petiolate, narrower than posterad abdominal segments.
14. Abdominal postsclerites IV subequal in length to, or greater in length than, abdominal postsclerites V–VI.
15. Abdominal tergite VII enlarged, not dome-like.

#### Gyne diagnosis.

See respective gyne-based diagnoses for *Protanilla* and *Leptanilla* below.

#### Male diagnosis.

As for the Leptanillinae, but Rs+M and *1m-cu* absent. Abdominal segment II with complete tergosternal fusion. Lateropenite present or absent; if present, then not articulated to parossiculus and never malleate.

#### Larval diagnosis.

See respective larval diagnoses for *Protanilla* and *Leptanilla* below.

### ﻿ Protanilla

Taxon classificationAnimaliaHymenopteraFormicidae

Taylor in Bolton, 1990b

393EA02E-F63D-5D71-9C5D-643026380A48

Fig. 23

Protanilla Taylor in Bolton, 1990b: 279. Type species: Protanillarafflesi Taylor in Bolton, 1990b, by monotypy.
Anomalomyrma Taylor in Bolton, 1990b: 278. Type species: Protanillataylori (Taylor in Bolton, 1990b), comb. nov., by monotypy. Syn. nov.
Furcotanilla Xu, 2012: 481. Type species: Protanillafurcomandibula Xu & Zhang, 2002, by original designation. Synonymy by Hsu et al. (2017). Holotype of P.furcomandibula not examined.

### ﻿*Protanillarafflesi* species group

*Protanillabeijingensis* Man, Ran, Chen & Xu, 2017.

*Protanillaconcolor* Xu, 2002.

*Protanillaeguchii* Satria, Putri & Ahda, 2023.

*Protanillaflamma* Baidya & Bagchi, 2020.

*Protanillafurcomandibula* Xu & Zhang, 2002.

*Protanillajongi*Hsu et al., 2017.

*Protanillalini* Terayama, 2009.

*Protanillarafflesi* Taylor in Bolton, 1990b.

*Protanillaschoedli* Baroni Urbani & de Andrade, 2006.

*Protanillatibeta* Xu, 2012.

*Protanillawardi* Bharti & Akbar, 2015.

### ﻿*Protanillabicolor* species group

*Protanillabicolor* Xu, 2002.

*Protanillagengma* Xu, 2012.

### *Protanillataylori* species group

*Protanillaboltoni* (Borowiec, Schultz, Alpert & Baňař, 2011), comb. nov.

*Protanillahelenae* (Borowiec, Schultz, Alpert & Baňař, 2011), comb. nov.

*Protanillataylori* (Taylor in Bolton, 1990b), comb. nov.

### ﻿Incertae sedis

﻿*Protanillaizanagi* Terayama, 2013.

**Worker diagnosis.**

1. Medial mandibular surface with or without (*Protanillataylori* species group) multiple rows of peg-like chaetae.
2. Medial mandibular margin with regularly spaced denticles.
3. Medial mandibular margin without teeth.
4. Ventromedial mandibular margin with or without subapical teeth.
5. Labrum with peg- or pencil-like chaetae (Fig. 21A, B).
6. Maxillary palp 4-merous.
7. Labial palp 2- or 1-merous.
8. Clypeus distinct, with epistomal sulcus present (Fig. 22A).
9. Dorsal mandibular articulation apparent in full-face view (Fig. 24B) or rarely not so (Fig. 24A) (*Protanillaconcolor*).
10. Medial chaetae on second protarsomere (Fig. 25A).
11. Meso-metapleural suture present, strongly impressed, scrobiculate.
12. Subpetiolar process present.
13. Abdominal segment III narrowly or broadly conjoined to abdominal segment IV.
14. Length of abdominal postsclerites IV greater than that of abdominal postsclerites V–VI.
15. Somal sculpture largely absent, if present then irregularly reticulate to rugose (*Protanillaboltoni* (Borowiec et al., 2011), comb. nov.).

**Gyne diagnosis.**

As in worker, but alate or rarely ergatoid; with compound eyes and 3 ocelli. If alate then venation Ogata Type IVb. M + Cu and Rsf3 absent; Rs + M, Cuf2-3, and *1m-cu* spectral or absent.

**Male diagnosis.**

1. Maxillary palp 4-merous.
2. Labial palp 2- to 1-merous.
3. Clypeus distinct.
4. Ocelli present, not set on tubercle.
5. Pronotum not anteroposteriorly prolonged.
6. Mesoscutum not anteroposteriorly prolonged.
7. Notauli present or absent.
8. Pterostigma present.
9. 1A in hindwing present or absent.
10. Upper metapleuron distinct from metapectal-propodeal complex.
11. Lower metapleuron indistinct from metapectal-propodeal complex.
12. Abdominal segment II petiolate.
13. Abdominal segment III petiolate or not.
14. Cupula present.
15. Volsellae present, parossiculus and lateropenite distinct.
16. Penial sclerites medially articulated.

**Larval diagnosis.**

Habitus pogonomyrmecoid. Cranium subelliptical in full-face view. Mandibles typhlomyrmecoid, without teeth, lateral surfaces smooth. Setae short, suberect. Ventral prothoracic process absent; larval hemolymph tap apparently absent.

**Figure 23.**
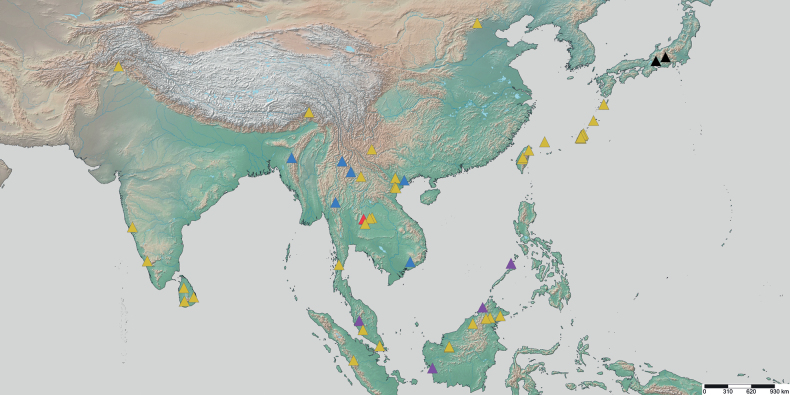
Geographical range of *Protanilla*. Locality information derived from AntWeb and available literature, visualized with SimpleMappr. Yellow = *Protanillarafflesi* species group; blue = *Protanillabicolor* species group; purple = *Protanillataylori* species group; red = *Protanilla* zhg-th02; black = *Protanillaizanagi*.

**Figure 24.**
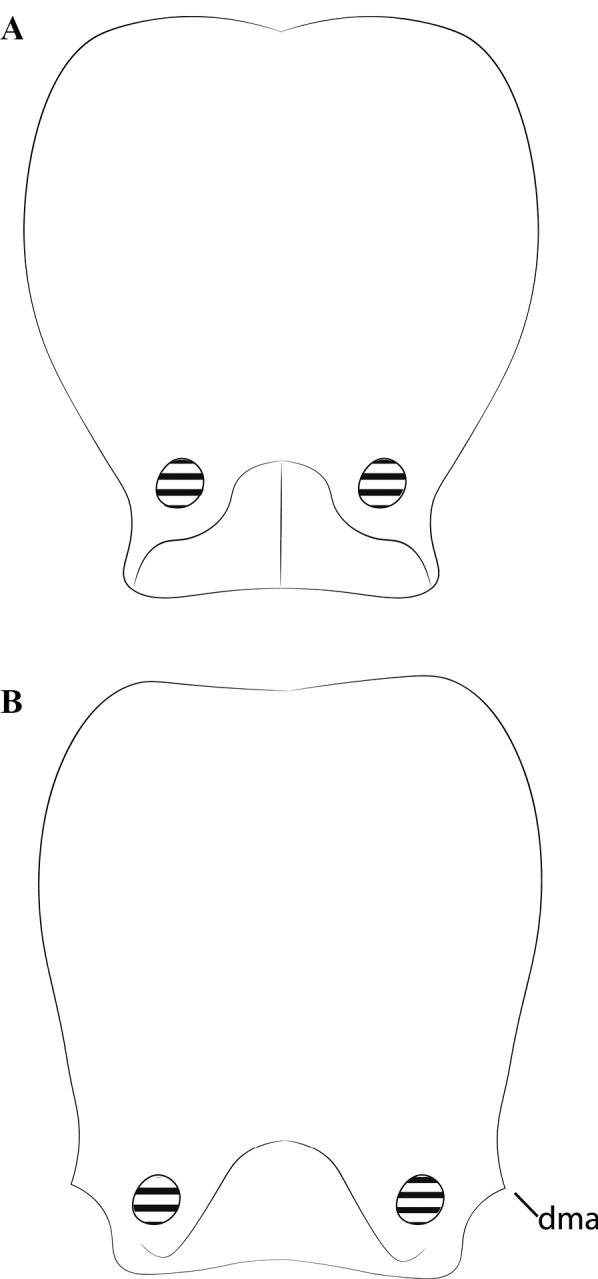
Worker cranium of *Protanillaconcolor* (**A**) and *Protanillabicolor* (**B**), diagrammatic full-face view, redrawn from Xu (2002: figs 18, 21). Abbreviation: dma = dorsal mandibular articulation.

**Figure 25.**
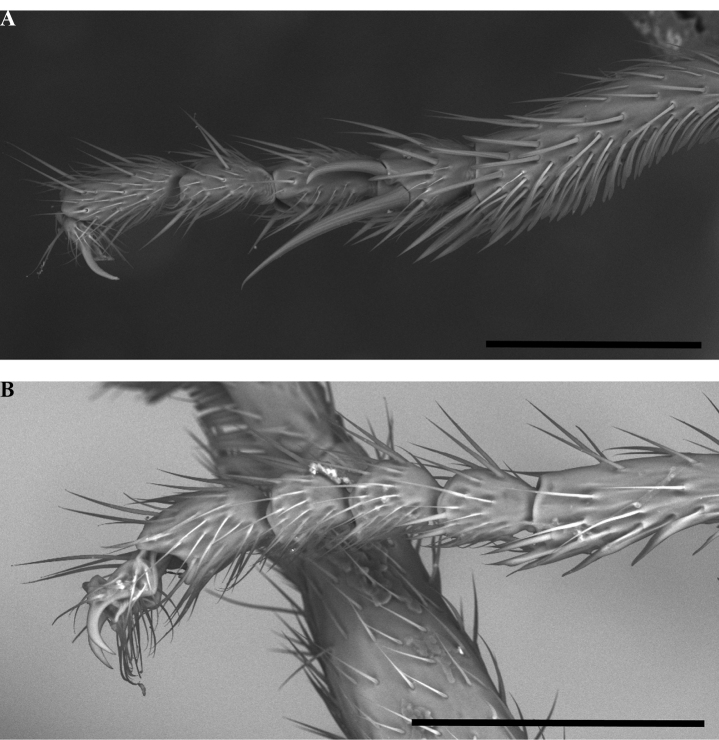
Condition of worker protarsus in *Protanilla* (**A**) and *Leptanilla* (**B**), profile view **A***Protanillalini* (CASENT0842702) **B***Leptanillabelantan* sp. nov. (MCZENT00793731). Scale bars: 0.1 mm.

**Remarks.**

The tribe Anomalomyrmini was erected by Taylor in Bolton (1990b) to include *Anomalomyrma* and *Protanilla*, which were both monotypic when established. Boudinot et al. (2022) merged the tribe into Leptanillini, although the Anomalomyrmini and Leptanillini sensu Bolton (1990b) are indubitably reciprocally monophyletic. All molecular phylogenetic inference (e.g., Borowiec et al. 2019; Griebenow 2020, pers. obs.) indicates the paraphyly of *Protanilla* relative to *Anomalomyrma*, with statistical support of varying strength. *Anomalomyrma* is therefore here synonymized with *Protanilla* (see “*Protanillataylori* species group” for explanation of nomenclatural priority). The phylogeny of *Protanilla* remains debatable (pers. obs.), with morphological diagnoses formulated below for the major lineages revealed by these analyses, here treated as informal monophyletic species groups. These lineages are recovered on deeply separated internal nodes (pers. obs.). *Protanillaizanagi* Terayama is left unplaced to species group due to an absence of molecular data for this species and bizarrely modified mandibles which exclude it from the species groups as diagnosed here. The position of *Protanilla* zhg-th02, known only from a single male specimen, is unstable across different phylogenomic analyses (pers. obs.), but is always situated on a long branch. This morphospecies does not conform to the male-based diagnoses of any of the species groups here delimited for which male morphology is known and does not represent the as-yet unknown male of the *Protanillataylori* species group. Based on this evidence, *Protanilla* zhg-th02 represents a major subclade of *Protanilla* for which workers remain to be discovered.

The *Protanillarafflesi* species group is further divided into three species complexes, with two distinctive species left unplaced to species complex. Species boundaries in *Protanilla* require further inquiry, with it being possible that the clade is over-split; each species complex may respectively represent a widespread, geographically variable species. Both sexes are notably conservative in terms of morphology. Robust species delimitation, reciprocally illuminated by morphometric and molecular data, is impossible with material as scanty as is available for *Protanilla*, so no revisions to species-level taxonomy within this clade are made here.

### ﻿*Protanillarafflesi* species group

**Worker diagnosis.**

**Figure 26.**
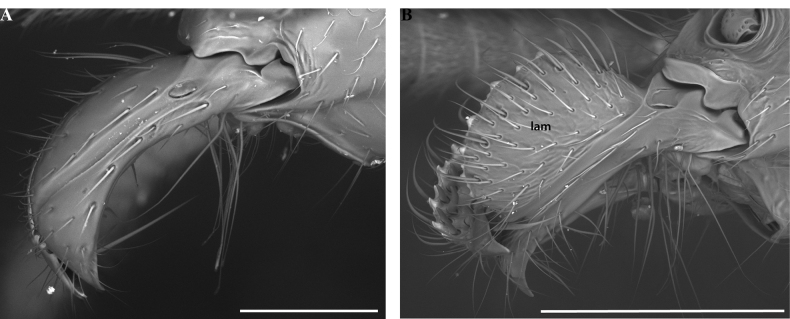
Worker mandibles in *Protanilla*, profile view **A***Protanillawallacei* (CASENT0842699) **B***Protanillaizanagi* (CASENT0842850). Abbreviation: lam = vertical dorsal lamella. Scale bars: 0.1 mm (**A**); 0.2 mm (**B**).

1. Medial mandibular surface armed with peg-like chaetae.
2. Mandible straight, not bowed along anteroposterior axis of cranium.
3. Vertical dorsal lamella absent from mandible (Fig. 26A).
4. Laterodorsal longitudinal groove present.
5. Clypeal surface flattened.
6. Median clypeal ridge externally visible.
7. Outline of clypeus in full-face view campaniform to oblate-trapezoidal.
8. Pronotal breadth subequal to propodeal breadth in dorsal view.
9. Mesotibia without spurs.
10. Petiole sessile.
11. Subpetiolar process with fenestra.
12. Abdominal sternite III convex, linear, or concave in profile view.
13. Abdominal segments II–III without tergotergal or sternosternal fusion.
14. Abdominal segments III–IV narrowly or broadly conjoined.
15. Anterior margin of abdominal post-tergite IV linear to strongly emarginate in dorsal view.
16. Soma concolorous.

**Gyne diagnosis.** As for genus, alate or ergatoid; if ergatoid than alar sclerites present.

**Male diagnosis.**

1. Distal 3 maxillary palpomeres of unequal lengths (Griebenow 2020: fig. 10A).
2. Labial palp 2- or 1-merous.
3. Antennomere 3 shorter than scape.
4. Antero-admedian signum present or absent; if present, then unsculptured.
5. Notauli present or absent; if present, then unsculptured.
6. Parapsidal lines present or absent.
7. 1A present in hindwing.
8. Abdominal segment III not petiolate.
9. Length of abdominal segment IV subequal to, or less than, respective lengths of abdominal segments V–VII.
10. Cupula non-annular.

**Larval diagnosis.** As for genus.

**Remarks.** This clade shows striking morphological conservatism in the worker caste and males, with their possibly being many cryptic species. *Protanillajongi* deviates from most of the clade in having broadly conjoined abdominal segments III–IV, and a ventral subapical mandibular tooth but is robustly confirmed to be nested well within the *P.rafflesi* species group by phylogenomic inference (pers. obs.). I therefore also place *P.furcomandibula* Xu & Zhang, 2002 in the *P.rafflesi* species group, as this species appears to be a close relative of *P.jongi* (Hsu et al. 2017), with the ventral subapical mandibular tooth being hypertrophied, and abdominal sternite II concave in profile view rather than linear to convex. The concavity of abdominal sternite II in profile view is homoplasious with the *Protanillataylori* species group, as is the broad connection of abdominal segments III–IV.

A 4,2 palpal formula was confirmed for the worker of *Protanillalini* by examination with micro-CT (Richter et al. 2021), while the palpal formula of the conspecific male was tentatively interpreted as 4,1 by Griebenow (2020). The palpal formula of the worker in the *Protanillarafflesi* species group, and indeed *Protanilla* as a whole, has largely gone unreported, with this study being the first to confirm the palpal formula of any representative of the *Protanillataylori* species group. Palpal formula across the Formicidae shows sexual monomorphism, with few exceptions (Bolton 2003; see sections on the *Protanillabicolor* species group and *Leptanillathai* species group below), meaning that the interpretation by Griebenow (2020) of the male labial palp in *P.lini* as 1-merous was in error.

Three species complexes are hereby recognized in the *Protanillarafflesi* species group: the *rafflesi* complex (*Protanillarafflesi* Taylor in Bolton, 1990b, *P.schoedli*, and *Protanillawardi* Bharti & Akbar, 2015); the *concolor* (*Protanillaconcolor* Xu, 2002; *Protanillatibeta* Xu, 2012; and *Protanillaeguchii* Satria, Putri & Ahda, 2023); and the *lini* complex (*P.lini*, *P.beijingensis*, *P.flamma*, and *P.wallacei*). Each of these complexes consist of species that are extremely similar, but for which material is too scarce to query interspecific boundaries. *Protanillafurcomandibula* and *P.jongi* are presumably close relatives, but are readily distinguishable based on known specimens, and so are not consigned to a species complex. Without phylogenomic inference, it is unclear if these species complexes are reciprocally monophyletic. *Protanillawallacei* sp. nov. based upon worker specimens is recovered as sister to *P.lini* (pers. obs.), as would be predicted based on observed worker phenotype.

A single specimen (CASENT0842639) of *Protanillabeijingensis* is herein reported from Khyber Pakhtunkhwa, Pakistan, in a remarkable range extension for a species heretofore known only from Beijing, China (Man et al. 2017). CASENT0842639 qualitatively differs from the type series in possessing a pair of peg-like chaetae on the labrum rather than a single median chaeta, but it is unknown whether this constitutes intra- or interspecific variation in *Protanilla*. This specimen is part of a series figured by Bolton (1990b: figs 1–6), for which coordinates are unavailable. Despite this, it appears that the collection was made at an elevation of 2400–2700 meters, in a cold temperate climate resembling that of the type locality.

Dias et al. (2019: 164) described the worker of *Protanillaschoedli* from ten specimens collected across Sri Lanka, based on “overall similarity in … general appearance” to the holotype gyne (CASENT0911228) and the implicit assumption that multiple *Protanilla* spp. cannot occur in sympatry. However, the putative worker *P.schoedli* display no more affinity to CASENT0911228 than to other members of the *Protanillarafflesi* species group, with the anterior margin of the petiolar node being straight (Dias et al. 2019: 164) rather than concave in profile view, as in CASENT0911228 (Baroni Urbani and de Andrade 2006: 46). The morphology of the petiolar node is not dimorphic between worker and gyne in *Protanilla*. This lack of concavity excludes these worker specimens from the *Protanillarafflesi* species complex to which *P.schoedli* belongs. The putative workers of *P.schoedli* (Dias et al. 2019) more closely resemble *Protanillaflamma* Baidya & Bagchi, 2020, but the difference in reported ranges of CI, SI, and PI between these two series supports their heterospecificity, if these morphometric differences reflect species boundaries. In this study, the putative *P.schoedli* (Dias et al., 2019) are regarded as an undescribed species belonging to the *Protanillalini* species complex. While neither *P.schoedli* nor *P.flamma* have been sequenced, other members of their respective species complexes have (*P.wardi* vs. *P.lini* and *P.wallacei*), with phylogenomic inference therefrom supporting their heterospecificity (pers. obs.).

The *Protanillarafflesi* species group contains some of the only *Protanilla* spp. for which bionomic data are available, with micro-computed tomographic studies of cephalic skeletomusculature in *P.lini* demonstrating the existence of “trap-jaw” capabilities in that species (Richter et al. 2021). The existence of putative trigger hairs across *Protanilla* (Griebenow et al. 2022: table 4) suggests that trap-jaw biology is a synapomorphy of the genus and paralleled in the Leptanillinae only by *Leptanillalaventa* (Griebenow, Moradmand, & Isaia in Griebenow, Isaia, & Moradmand, 2022), comb. nov.

### ﻿*Protanillabicolor* species group

**Worker diagnosis.**

1. Medial mandibular margin armed with peg-like chaetae.
2. Mandible straight, not bowed along anteroposterior axis of cranium.
3. Vertical dorsal lamella absent from mandible.
4. Laterodorsal longitudinal groove absent.
5. Clypeal surface concave.
6. Median clypeal ridge not externally visible.
7. Outline of clypeus in full-face view campaniform.
8. Breadth of pronotum subequal to propodeum in dorsal view.
9. Mesotibia with 1 spur.
10. Petiole sessile.
11. Subpetiolar process with fenestra.
12. Abdominal sternite III convex in profile view.
13. Abdominal segments II–III without tergotergal and sternosternal fusion.
14. Abdominal segments III and IV narrowly joined.
15. Anterior margin of abdominal post-tergite IV linear to slightly emarginate in dorsal view.
16. Soma bicolored, rarely concolorous.

**Gyne diagnosis.** As for genus, ergatoid, without alar sclerites (pers. obs.).

**Male diagnosis.**

1. Distal 3 maxillary palpomeres subequal in length (Griebenow 2020: fig. 10B).
2. Labial palp 2-merous.
3. Antennomere 3 longer than scape.
4. Antero-admedian signum absent.
5. Notauli present, scrobiculate.
6. Parapsidal lines absent.
7. 1A absent from hindwing.
8. Abdominal segment III petiolate.
9. Length of abdominal segment IV subequal to, or exceeding, combined length of abdominal segments V–VIII.
10. Cupula annular (Griebenow et al. in press).

**Larval diagnosis.** Larva unknown.

**Remarks.** Phenotypic differentiation between the *Protanillabicolor* and *Protanillarafflesi* species groups in the worker caste is comparatively slight, but the two clades are discretely distinguishable by tibial spur formula. The strong concavity of the anterior clypeal margin referred to in previous descriptive literature more correctly refers to the face of the clypeus: the anterior margin itself is in fact no more emarginate in this clade than in the *Protanillarafflesi* species group. The morphology of *Protanilla* TH03, a male singleton attributable to this clade by molecular data (e.g., Borowiec et al. 2019), and that of male *P.gengma* (Griebenow et al. in press) differs from all other known males of *Protanilla* in multiple respects, most conspicuously in petiolation of abdominal segment III: this condition is unique among male Leptanillinae.

Workers of the *Protanillabicolor* species group are unique among examined *Protanilla* workers in exhibiting a mesotibial spur, an apparent symplesiomorphy of this clade. Palpal formula could not be assessed in the worker caste due to a lack of fresh specimens, but given sexual monomorphism of palpal formula across the Formicidae save for the Ponerini, *Typhlomyrmex* (Bolton 2003), and probably the *Leptanillathai* species group as well (this study), it is sound to predict a 4,2 formula.

Species boundaries in the *Protanillabicolor* species group remain unclear. Specimens identified as *P.gengma* are known to vary in labral chaeta count according to geographical origin (Aswaj et al. 2020), but the relevance of this trait to species delimitation is unknown. *Protanilla* VN03 appears transitional in morphometric terms between *Protanillabicolor* Xu, 2002 and *P.gengma*, but PTL in *Protanilla* VN03 falls outside the range observed in either of these species.

### ﻿*Protanillataylori* species group

**Worker diagnosis.**

1. Medial mandibular surface without peg-like chaetae.
2. Mandible straight, not bowed along anteroposterior axis of cranium.
3. Vertical dorsal lamella absent or present (*Protanillataylori* (Taylor in Bolton, 1990b)) on mandible.
4. Laterodorsal longitudinal groove present.
5. Clypeal surface concave.
6. Median clypeal ridge not externally visible.
7. Outline of clypeus in full-face view an oblate trapezoid.
8. Pronotal breadth greater than propodeal breadth in dorsal view.
9. Mesotibia without spurs.
10. Petiole subsessile.
11. Subpetiolar process with fenestra present or absent.
12. Abdominal sternite II convex in profile view.
13. Abdominal segments II-III without tergotergal and sternosternal fusion.
14. Abdominal segment III broadly joined to abdominal segment IV.
15. Anterior margin of abdominal tergite IV entire in dorsal view.
16. Soma concolorous.

**Gyne diagnosis.** As for worker, but, alate. Pencil-like chaetae present on mandible; two or three rows of cuticular denticles along masticatory margin.

**Male diagnosis.** Male unknown.

**Larval diagnosis.** Larva unknown.

**Remarks.***Anomalomyrma* was established for *Protanillataylori* comb. nov. by Taylor in Bolton (1990b) on account of derived mandibular morphology and the tergotergal and sternosternal fusion of abdominal segments II–III, a character state unique among the Formicidae (Bolton 1990b, Borowiec et al. 2011). While *P.taylori* is known only from the gyne, Borowiec et al. (2011) described *Protanillaboltoni* (Borowiec, Schultz, Alpert & Baňař, 2011), comb. nov. and *Protanillahelenae* (Borowiec, Schultz, Alpert & Baňař, 2011), comb. nov. based on worker material, and refined the diagnosis of *Anomalomyrma*, demonstrating that the presence of a vertical mandibular lamella was of no diagnostic utility in the Anomalomyrmini at the genus level, and predicting that the resemblance between the mandibles of *Anomalomyrma* and the then-undescribed *Protanillaizanagi* (see below) was homoplasious. This hypothesis has not yet been tested with phylogenomic inference.

Given the paraphyly of *Protanilla* relative to *Anomalomyrma* under phylogenomic inference from several differently curated datasets (pers. obs.), the latter genus is synonymized under *Protanilla*. These names were established in the same publication (Bolton 1990b), and the latter is here given precedence as permitted in Article 24.2 of the International Code of Zoological Nomenclature. The *Protanillataylori* species group is equivalent to the former genus *Anomalomyrma*.

The vertical dorsal lamella in *Protanillataylori* and *P.izanagi* has few parallels within the Formicoidea, being comparable to the morphology observed in both female and male beast ants (Camelomeciidae: *Camelosphecia*), which are known only from Cretaceous burmite (Boudinot et al. 2020). Among extant formicoids, the mandible of these two *Protanilla* spp. is most reminiscent of that observed in armadillo ants (Agroecomyrmecinae: Agroecomyrmecini: *Tatuidristatusia* Brown & Kempf, 1968), which is likewise bowed, but with the masticatory margin armed with a brush of robust feathery setae (Brown and Kempf 1968: fig. 3) rather than peg-like chaetae, cuticular denticles, or both.

The feeding ecology of *P.taylori* and *P.izanagi* may therefore resemble that of the armadillo ants. Brown and Kempf (1968: 189) hypothesized that armadillo ants feed on “slippery or active arthropod prey”, with William Brown speculating that these ants were specialist predators of oligochaetes (P. S. Ward, pers. comm. 2021). Given that known ant specialists on oligochaete prey, such as *Psalidomyrmexprocerus* Emery (Formicidae: Ponerinae: Ponerini) (Lévieux 1983; Déjean et al. 1999), have mandibles quite unlike those of armadillo ants, this seems improbable. Food court experiments to determine the diet of these ants were unsuccessful, but isotopic analysis of armadillo ant tissue suggests that the unknown prey is itself predatory (Jacquemin et al. 2014: 5).

*Protanillataylori* and *Protanilla* id01 differ notably from the species known only from workers in the presence of two and three ranks, respectively, of produced denticles on the mandible (Bolton 1990b; this study), as opposed to the condition observed in most *Protanilla*; with the presence of pencil-like chaetae on the mandible, which are absent in the worker-based species. The worker and gyne caste remain unassociated in all three described species of the *Protanillataylori* species group, plus *Protanilla* id01. Until the female castes respectively unknown from these species are discovered, we cannot determine whether observed mandibular differences are to be credited to allospecificity, or to caste dimorphism.

### ﻿Incertae sedis

﻿*Protanillaizanagi* Terayama.

**Worker diagnosis.**

1. Medial mandibular surface with peg-like chaetae.
2. Mandible bowed along anteroposterior axis of cranium (Fig. 17B).
3. Vertical dorsal lamella present on mandible (Fig. 26B).
4. Laterodorsal longitudinal groove present on mandible.
5. Clypeal surface flattened.
6. Median clypeal ridge not externally visible.
7. Outline of clypeus in full-face view an oblate trapezoid.
8. Pronotal breadth greater than propodeal breadth in dorsal view.
9. Mesotibia without spurs.
10. Petiole sessile.
11. Subpetiolar process with fenestra present.
12. Abdominal sternite II convex in profile view.
13. Abdominal segments II–III without tergotergal and sternosternal fusion.
14. Abdominal segment III narrowly joined to abdominal segment IV.
15. Anterior margin of abdominal tergite IV entire in dorsal view.
16. Soma concolorous.

**Gyne diagnosis.**

As for genus, alate.

**Male diagnosis.**

Male unknown.

**Larval diagnosis.**

Larva unknown.

**Remarks.**

Prior to formal description, this peculiar species from southern Honshu was cited by Hölldobler and Wilson (1990) and Imai et al. (2003) as *Anomalomyrma* (the former authors referring to it under the *nomen nudum Anomalomyrmakubotai*), due to the presence of an erect mandibular lamella. Borowiec et al. (2011) concluded that this character state alone was insufficient to place the morphospecies in *Anomalomyrma*, with its habitus being otherwise consistent with that of *Protanilla*. Terayama (2013) accordingly described *Protanillaizanagi* in that genus. The presence of distinct posterior faces on the dorsal petiolar and post-petiolar nodes, with abdominal segments III and IV not being broadly conjoined, shows an affinity to the *Protanillarafflesi* and *Protanillabicolor* species groups, but these character states are plesiomorphic for *Protanilla* (pers. obs.). It is likely that the similar mandibular morphology of *P.izanagi* and the *Protanillataylori* species group reflects similar diet (see “Remarks” for the *Protanillataylori* species group above) and is therefore homoplasious (Borowiec et al. 2011). Terayama (2013) describes the compound eye as being absent in the worker, but the specimens that I examined are remarkable in the retention of two ommatidia (Fig. 17A). The presence of any trace of the compound eye in the worker is unique among the Leptanillinae. No molecular data are available for *P.izanagi*, and so in the absence of compelling morphological evidence, this species must be left unplaced to species group within *Protanilla*. I predict, however, that molecular data will demonstrate that *Protanillaizanagi* belongs within the *Protanillarafflesi* species group.

### ﻿ Leptanilla

Taxon classificationAnimaliaHymenopteraFormicidae

Emery, 1870

30B57B9D-F50F-5050-B2D8-349B4CD011EF

Leptanilla Emery, 1870: 196. Type species: Leptanillarevelierii Emery, 1870, by monotypy.
Scyphodon Brues, 1925: 93. Type species: Leptanillaanomala (Brues, 1925), comb. nov., by monotypy. Holotype of L.anomala examined; deposited at MHNG. Syn. nov.
Phaulomyrma Wheeler & Wheeler, 1930: 193. Type species: Leptanillajavana (Wheeler & Wheeler, 1930), by original designation. Holotype of L.javana examined; deposited at MCZC. Synonymy by Griebenow (2021).
Leptomesites Kutter, 1948: 286. Type species: Leptanillaescheri (Kutter, 1948), by monotypy. Holotype of L.escheri examined; deposited at MZLS. Synonymy by Baroni Urbani (1977).
Noonilla Petersen, 1968: 582. Type species: Leptanillacopiosa (Petersen, 1968), by monotypy. Holotype of L.copiosa not examined; deposited at NHMD. Syn. nov.
Yavnella Kugler, 1987 (“1986”): 52. Type species: Leptanillaargamani (Kugler, 1987 (“1986”)), by original designation. Holotype of L.argamani not examined; deposited at TAU. Syn. nov.

### ﻿*Leptanillathai* species group

*Leptanillaargamani* (Kugler, 1987 (“1986”)), comb. nov.

*Leptanillabelantan* sp. nov.

*Leptanillaescheri* (Kutter, 1948).

*Leptanillaindica* (Kugler, 1987 (“1986”)), comb. nov.

*Leptanillajudaica* Kugler, 1987 (“1986”).

*Leptanillakunmingensis* Xu & Zhang, 2002.

*Leptanillalamellata* Bharti & Kumar, 2012.

*Leptanillalaventa* (Griebenow, Moradmand, & Isaia in Griebenow, Isaia, & Moradmand, 2022), comb. nov.

*Leptanillathai* Baroni Urbani, 1977.

*Leptanillaujjalai* Saroj, Mandi & Dubey, 2022.

### ﻿*Leptanillahavilandi* species group

*Leptanillaanomala* (Brues, 1925), comb. nov.

*Leptanillacopiosa* (Petersen, 1968), comb. nov.

*Leptanillahavilandi* Forel, 1901.

### ﻿*Leptanillabethyloides* species group

*Leptanillabethyloides* sp. nov.

### ﻿*Leptanillanajaphalla* species group

*Leptanillanajaphalla* sp. nov.

### ﻿*Leptanillarevelierii* species group

*Leptanillaacherontia* sp. nov.

*Leptanillaafricana* Baroni Urbani, 1977.

*Leptanillaalexandri* Dlussky, 1969.

*Leptanillaastylina* Petersen, 1968.

*Leptanillaaustralis* Baroni Urbani, 1977.

*Leptanillabesucheti* Baroni Urbani, 1977.

*Leptanillabifurcata* Kugler, 1987 (“1986”).

*Leptanillaboltoni* Baroni Urbani, 1977.

*Leptanillabuddhista* Baroni Urbani, 1977.

*Leptanillacharonea* Barandica, López, Martínez & Ortuño, 1994.

*Leptanilladoderoi* Emery, 1915.

*Leptanillaexigua* Santschi, 1908.

*Leptanillahunanensis* Tang, Li & Chen, 1992.

*Leptanillaislamica* Baroni Urbani, 1977.

*Leptanillaisraelis* Kugler, 1987 (“1986”).

*Leptanillajaponica* Baroni Urbani, 1977.

*Leptanillajavana* (Wheeler & Wheeler, 1930).

*Leptanillakubotai* Baroni Urbani, 1977.

*Leptanillamacauensis* Leong, Yamane, & Guénard, 2018.

*Leptanillaminuscula* Santschi, 1907.

*Leptanillamorimotoi* Yasumatsu, 1960.

*Leptanillanana* Santschi, 1915.

*Leptanillaoceanica* Baroni Urbani, 1977.

*Leptanillaokinawensis* Terayama, 2013.

*Leptanillaortunoi* López, Martínez, & Barandica, 1994.

*Leptanillaplutonia* López, Martínez, & Barandica, 1994.

*Leptanillapoggii* Mei, 1995.

*Leptanillarevelierii* Emery, 1870.

*Leptanillaswani* Wheeler, 1932.

*Leptanillataiwanensis* Ogata, Terayama & Masuko, 1995.

*Leptanillatanakai* Baroni Urbani, 1977.

*Leptanillatanit* Santschi, 1907.

*Leptanillatenuis* Santschi, 1907.

*Leptanillatheryi* Forel, 1903.

*Leptanillavaucheri* Emery, 1899.

*Leptanillayunnanensis* Xu, 2002.

*Leptanillazaballosi* Barandica, López, Martínez & Ortuño, 1994.

### ﻿Incertae sedis

*Leptanillabutteli* Forel, 1913.

*Leptanillaclypeata* Yamane & Ito, 2001.

*Leptanillahypodracos* Wong & Guénard, 2016.

*Leptanillakebunraya* Yamane & Ito, 2001.

*Leptanillapalauensis* (Smith, 1953).

### ﻿Unplaced to species group

﻿*Leptanillasantschii* Wheeler & Wheeler, 1930.

**Worker diagnosis.**

1. Medial mandibular margin without peg-like chaetae.
2. Medial mandibular margin with or without denticles, if present then irregularly spaced.
3. Medial mandibular margin with at least one subapical tooth.
4. Ventromedial mandibular margin without subapical teeth.
5. Labrum without peg-like chaetae.
6. Maxillary palp 1- to 2-merous.
7. Labial palp 1-merous.
8. Clypeus indistinct.
9. Dorsal mandibular articulation not visible in full-face view.
10. Medial chaetae absent from second protarsomere (Fig. 25B).
11. Meso-metapleural suture usually vestigial to absent, rarely present; if present then unsculptured.
12. Subpetiolar process present or absent.
13. Abdominal segment III narrowly joined to abdominal segment IV.
14. Length of abdominal postsclerites IV longer than or subequal to that of abdominal postsclerites V–VI.
15. Somal sculpture present and widespread, never punctate.

**Gyne diagnosis.**

Dichthadiiform, and therefore lacking wings and axillary sclerites. Mandibles edentate or with three teeth (*Leptanillakubotai*) (Terayama and Kinomura 2015). Compound eyes repressed or present; if present then consisting of one or two ommatidia. Abdominal segment III never petiolate.

**Male diagnosis.**

1. Maxillary palp 1- to 2-merous.
2. Labial palp 1-merous.
3. Clypeus distinct or indistinct.
4. Ocelli present or absent (*Leptanilla* TH03, *Leptanilla* zhg-bt03); if present then set on tubercle or rarely not (e.g., *Leptanillanajaphalla* sp. nov.).
5. Pronotum anteroposteriorly prolonged.
6. Mesoscutum anteroposteriorly prolonged.
7. Notauli absent.
8. Pterostigma absent.
9. 1A absent from hindwing.
10. Upper metapleuron distinct from metapectal-propodeal complex (*Leptanillathai* species group, *Leptanillabethyloides* sp. nov., *Leptanilla* zhg-th01) or indistinct.
11. Lower metapleuron indistinct or distinct from metapectal-propodeal complex (*Leptanillahavilandi* species group, *Leptanillabethyloides* sp. nov., *Leptanilla* zhg-th01).
12. Abdominal segment II petiolate or not (e.g., *Leptanilla* TH02).
13. Abdominal segment III not petiolate.
14. Cupula present or absent; if present, then annular.
15. Volsellae present or absent (*Leptanillahavilandi* species group, *Leptanillabethyloides* species group), if present then parossiculus and lateropenite indistinct (Griebenow et al. in press).
16. Penial sclerites medially fused or articulated (*Leptanillaastylina* Petersen, 1968), rarely partly articulated (*Leptanilla* TH03).

**Larval diagnosis.**

Habitus leptanilloid. Cranium subpyriform in full-face view. Mandibles leptanilloid, with teeth, lateral surface shagreened with spinules. Setae short and suberect or flexuous, elongated, and subdecumbent to erect. Ventral prothoracic process and hemolymph taps present.

**Remarks.**

The four genera known solely from males at the time of Bolton (1990b) were provisionally retained in the Leptanillini by that author, with the knowledge that at least some would prove to be satellite genera of *Leptanilla*. The phylogeny of the Leptanillini is now robustly resolved with phylogenomic and total-evidence approaches: *Leptanilla* s. l. (Griebenow 2020, 2021) includes *Scyphodon* and *Noonilla* (= *Scyphodon* s. l.; Griebenow et al. in press), with *Leptanilla* s. str., with which *Phaulomyrma* was synonymized (Griebenow 2021); and is sister to a well-supported clade first recovered by Borowiec et al. (2019) and identified as *Yavnella* by Griebenow (2020, 2021).

The question of the formal rank of major subclades in the Leptanillini depends upon practical utility. For generic ranking of subclades to be useful, these clades must be distinguishable based upon the morphology of both the male sex and available female castes. *Yavnella* and *Leptanilla* s. l. are readily diagnosed based upon males, as are the subclades of *Leptanilla* s. l. (pers. obs.). The taxonomic problem then lies in whether these groups can be distinguished based upon worker morphology.

**Figure 27.**
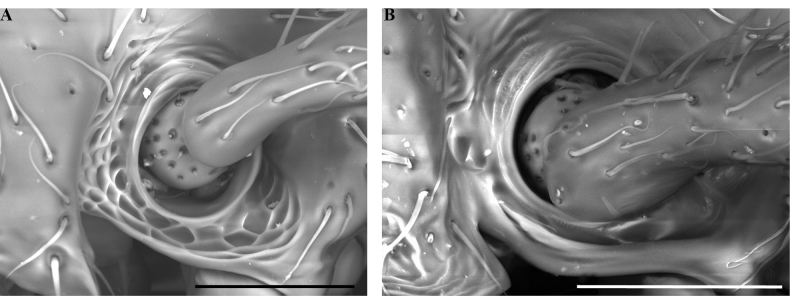
Antennal torulus in *Leptanillathai* (**A**) and *Leptanillahavilandi* (**B**), worker. Scale bars: 0.04 mm (**A**); 0.05 mm (**B**).

Using phylogenomic inference, Griebenow et al. (2022) identified the worker of *Yavnella*, while *Leptanillahavilandi* Forel is sister to *Scyphodon* s. l. (in those analyses represented only by *Noonilla* spp.) and *Leptanillathai* is robustly recovered within *Yavnella* as well (pers. obs.). The morphological similarities between *Leptanillalaventa* (Griebenow et al. 2022), comb. nov. and *L.thai* to the exclusion of *Leptanilla* s. str., such as the emarginate frontoclypeal process, cannot be interpreted as synapomorphic. *L.havilandi* and *thai* are extremely close morphologically, as noted by Baroni Urbani (1977). In this study, I find that these two species are discriminated by areolate sculpturation of the torulus in *L.thai* (no such sculpture is observed in *L.havilandi*; Fig. 27), different mandibular dentition, and a more elevated frontoclypeal process in *L.havilandi*. Sculpturation requires scanning electron microscopy to be assessed, while elevation of the frontoclypeal process and mandibular dentition are difficult to accurately assess with light microscopy (as evidenced by the incorrect accounting of mandibular teeth in the description of *L.thai* (Baroni Urbani, 1977)), making these characters impractical for identification of leptanilline workers to genus. This impracticality, and lack of consistent morphological distinction between the worker castes across all *Yavnella* and *Leptanilla*, argues against maintaining the two as separate genera.

Therefore, the most conservative course of nomenclatural action is to synonymize *Scyphodon*, *Noonilla*, and *Yavnella* under *Leptanilla*. The diversity of *Leptanilla* is here organized in informal species groups, for which diagnoses based upon all known castes are provided below. Wherever sampling of molecular data across *Leptanilla* is sufficient for phylogeny of these species groups to be known, these are delimited to be monophyletic. Several aberrant species for which molecular data are unavailable are left unplaced to species group.

### ﻿*Leptanillathai* species group

**Worker diagnosis.**

1. Mandible with 3–4 teeth.
2. Maxillary palp 1- to 2-merous.
3. Frontoclypeal process present, apex emarginate.
4. Lateral clypeal teeth absent.
5. Meso-metapleural groove absent or present (*Leptanillakunmingensis* Xu & Zhang, 2002).
6. Mesotibia with two spurs.
7. Metatibia with 1–2 spurs.
8. Length of abdominal segment II subequal to width in dorsal view, or length much greater than width (*Leptanillalaventa*).
9. Anterior of abdominal tergite IV lateromedially constricted in dorsal view (*Leptanillalaventa*) or not lateromedially constricted.
10. Length of abdominal tergite IV greater than combined length of posterior abdominal tergites in dorsal view.

**Gyne diagnosis.** As for genus, but petiole longer than broad in dorsal view, outline rectangular (*Leptanillaescheri*) to subpyriform (*Leptanillabelantan*). Placement of these two species in the *Leptanillathai* species group is provisional (see Remarks).

**Male diagnosis.**

1. Mandalus ≥ 0.5× length of that of the mandible.
2. Mandible fused to cranium, rarely articulated.
3. Anteromedian ocellus orthogonally dorsal to compound eye in profile view.
4. LF2 > SL, rarely LF2 ≈ SL.
5. Distal transverse carina absent from procoxa.
6. Protrochanter not elongated.
7. Profemur not enlarged, sometimes proximally kurtotic.
8. Arcuate medial carina absent from profemur.
9. Apicoventral hook absent from profemur.
10. Ventromedian carina absent from protibia.
11. Protibial comb absent.
12. Antero-admedian signum present or absent.
13. Pronotum and mesoscutum not anteroposteriorly prolonged.
14. Mesoscutellum without recurved posteroventral process.
15. Adventitious spectral M+Cu absent from forewing.
16. Upper metapleuron distinct from metapectal-propodeal complex or indistinct.
17. Lower metapleuron indistinct from metapectal-propodeal complex.
18. Propodeal declivity concave in profile view.
19. Petiole without distinct dorsal node.
20. Abdominal sternite II without ventral process.
21. Abdominal tergite VIII broader than long in posterodorsal view.
22. Abdominal sternite IX posteriorly separate from gonocoxites.
23. Mulceators absent.
24. Cupula present.
25. Gonopodites inarticulate.
26. Gonocoxites with partial ventromedian fusion.
27. Gonocoxites without or rarely with dorsomedian fusion (*Leptanilla* TH03).
28. Gonocoxites partly fused to penial sclerites or unfused.
29. Gonostyli present or rarely absent (*Leptanilla* TH03).
30. Volsellae present.
31. Volsellae medially separate.
32. Volsella furcated, sometimes entire (*Leptanilla* TH03, *Leptanilla* zhg-bt03).
33. Penial sclerites usually with complete median fusion, rarely with partial median fusion.
34. Penial sclerites dorsoventrally compressed or not (*Leptanilla* TH03).
35. Phallotreme apical.
36. Phallotreme dorsal.
37. Dense phallotremal vestiture of setae absent.

**Larval diagnosis.** As for genus. Larva is known only in *Leptanillaescheri* and *Leptanillajudaica*, the placement of which in this species group has not been confirmed by molecular phylogenetic inference.

**Figure 28.**
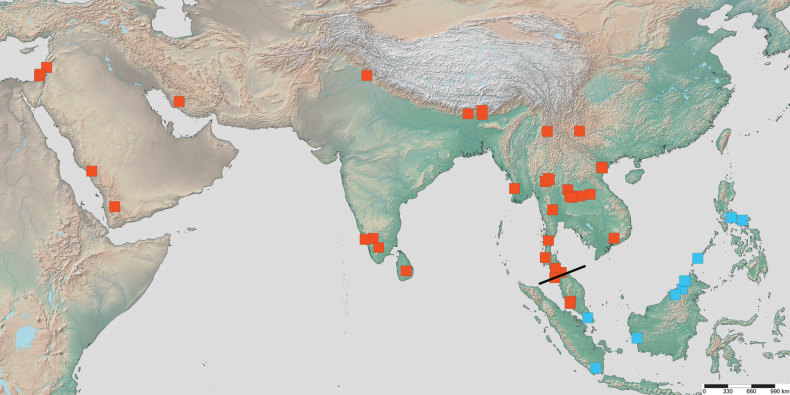
Geographical range of the *Leptanillathai* species group (orange) and the *Leptanillahavilandi* species group (blue). The Pattani-Kangar Line is indicated in black. Locality information derived from AntWeb and available literature, visualized with SimpleMappr.

**Remarks.***Leptanillaescheri*, *L.judaica*, *Leptanillakunmingensis* Xu & Zhang, 2002, *Leptanillalamellata* Bharti & Kumar, 2015, *L.ujjalai*, and *L.belantan* sp. nov. are placed in this species group with some caution, given a lack of molecular data for these species. These four species bear some resemblance to *Leptanillalaventa* comb. nov. (e.g., in the palpal formula being 2,1), which differs from them only in the elongation of the appendicular sclerites. Since worker morphology in *Leptanilla* is often indecisive when inferring phylogeny, or downright misleading (pers. obs.), these species may belong elsewhere within *Leptanilla*. With only species included in phylogenomic analysis under consideration, the *Leptanillathai* and *Leptanillahavilandi* species groups are mutually indistinguishable based upon worker morphology without examination of cranial microsculpture. However, male specimens of the *Leptanillahavilandi* species group are known only from the Sundan region, and so extralimital worker specimens that conform to the worker-based morphological diagnosis of that species group presented here are instead referred to the *Leptanillathai* species group. These two clades are only definitively known in sympatry from peninsular Malaysia (Fig. 28). Since phylogenomic inference confirms the position of *L.thai* within the former genus *Yavnella*, and this is the oldest species name assigned to that clade for which that hypothesized placement can be confirmed with molecular data, this clade is informally exemplified by that species.

As noted in Griebenow et al. (2022), the anatomical identity of the frontoclypeal process observed in the *Leptanillathai* species group, the *Leptanillahavilandi* species group, *Leptanillaclypeata* and *Leptanillahypodracos* Wong & Guénard, 2016 is unclear. Prior authors assumed a clypeal origin, which may be in part correct, but this hypothesis cannot be tested with external examination due to the absence in worker *Leptanilla* of apparent anterior tentorial pits or an unequivocal epistomal sulcus. Elision of the boundaries between the frons and clypeus also occurs in *Discothyrea* (Proceratiinae) and *Aulacoponerelicta* Arnol’di, 1930 (Ectatomminae: Heteroponerini), likewise involved in an anteromedian projection from the cranium in full-face view (Taylor 1979). Detailed micro-CT study of the shelf-like frontoclypeal process in the *Discothyreaoculata* and *Discothyreatraegordhi* species complexes was able to confirm the identity of this process as a mosaic of the frons and clypeus (Hita-Garcia et al. 2019), and only similar data can possibly be used to clarify the anatomy of the frontoclypeal process in *Leptanilla*.

The palpal formula in the worker caste of *L.thai* and *L.laventa* is 2,1 (Griebenow et al. 2022), which, among those species that have been confirmed to belong to the *Leptanillathai* species group by phylogenomic inference, are the only ones for which the worker caste is known. All known males of the *Leptanillathai* species group examined in this study possess a 1-merous palp (cf. Kugler 1987), meaning that it is probable that the *Leptanillathai* species group shows sexual dimorphism in palpal formula. This would be only confirmed by definitive association of conspecific worker and male specimens belonging to this clade. If confirmed, the *Leptanillathai* species group would constitute only the third independent origin in the Formicidae of decoupled palpal formula between the sexes (Bolton 2003). Curiously, this would run opposite to the tendency in other cases of decoupling within the Formicidae, in which the palpomere counts of the worker are reduced relative to those in the male.

The *Leptanillathai* species group is broadly distributed across southern Asia (Griebenow et al. 2022: fig. 20), with males being more diverse and abundant than any other leptanilline clade in Malaise trap residues from mainland Southeast Asia. An undescribed male morphospecies is recorded from Sana’a, Yemen (Collingwood and Agosti 1996), meaning that the *Leptanillathai* species group extends at least to the extreme northeastern corner of the Afrotropics, but within that ecozone is perhaps restricted to the southern Arabian Peninsula. No specimens are yet known from the Eastern Palaearctic, with the nearest examples being *L.kunmingensis* and an undescribed worker specimen (CASENT0064302), both from Yunnan Province, China. This absence from the Eastern Palaearctic is notable given the thorough myrmecological sampling of Japan and to a lesser extent Taiwan. Better sampling of the Sundan region is needed, but members of the *Leptanillathai* species group are conspicuously rare in collections from this area compared to mainland Southeast Asia, with only two male morphospecies being known from a single locality south of the Pattani-Kangar Line (Whitmore 1988), with *Leptanillabelantan*, which may represent the worker of either of these. It may be surmised from the distribution of the *Leptanillathai* species group that this clade originated in subtropical seasonal forests of mainland Southeast Asia or the Indian subcontinent, explosively radiating in the former region and arid habitats of the Western Palaearctic and (marginally) the Afrotropics. The *Leptanillathai* species group appears to have been mostly unsuccessful in penetrating perhumid equatorial rainforests. I propose that preoccupation of ecological niche space in the Sundan region by the *Leptanillahavilandi* species group is perhaps responsible, given the close functional similarities between the worker phenotypes in these two clades to the exclusion of confirmed worker morphology in the *Leptanillarevelierii* species group.

### ﻿*Leptanillahavilandi* species group

**Worker diagnosis.**

1. Mandible with three teeth.
2. Maxillary palpomere 2-merous.
3. Frontoclypeal process present, apex emarginate.
4. Lateral clypeal teeth absent.
5. Meso-metapleural suture absent.
6. Mesotibia with two spurs.
7. Metatibia with two spurs.
8. Length of abdominal segment II subequal to width in dorsal view.
9. Anterior of abdominal tergite IV not lateromedially constricted in dorsal view.
10. Length of abdominal tergite IV greater than combined length of posterior abdominal tergites in dorsal view.

**Gyne diagnosis.** Gyne unknown.

**Male diagnosis.**

**Figure 29.**
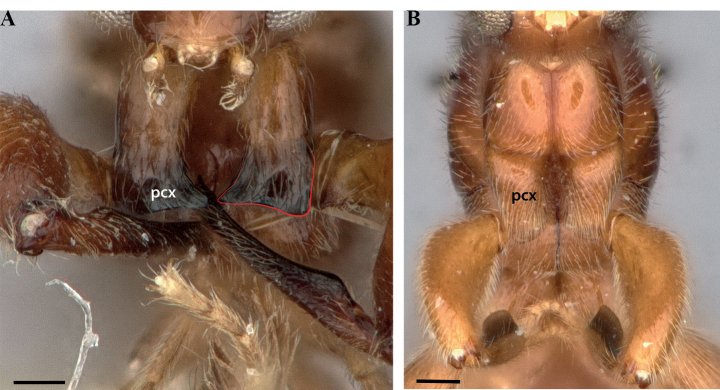
Condition of the male procoxa in *Leptanilla*, anterior view. Distal procoxal carina outlined in red **A**Leptanillacf.copiosa (CASENT0842844) **B***Leptanilla* zhg-my04 (CASENT0842567). Abbreviation: pcx = procoxa. Scale bars: 0.1 mm.

1. Mandalus ≥ 0.5× length of the mandible or < 0.5× length of mandible.
2. Mandible never fused to cranium, fully articulated.
3. Anteromedian ocellus orthogonally dorsal to compound eye in profile view or posterior to compound eye.
4. LF2 < SL, rarely LF2 ≈ SL (*Leptanillacopiosa* (Petersen, 1968)).
5. Distal transverse carina present on procoxa (Fig. 29A).
6. Protrochanter not elongated.
7. Profemur not enlarged, or moderately enlarged, sometimes proximally kurtotic.
8. Arcuate medial carina absent from profemur.
9. Apicoventral hook absent from profemur.
10. Ventromedian carina present on protibia.
11. Protibial comb absent.
12. Antero-admedian signum present or absent.
13. Pronotum and mesoscutum anteroposteriorly prolonged.
14. Mesoscutellum without recurved posteroventral process.
15. Adventitious spectral M+Cu absent from forewing.
16. Upper metapleuron indistinct from metapectal-propodeal complex.
17. Lower metapleuron usually distinct from metapectal-propodeal complex, rarely (*L.anomala* (Brues, 1925)) indistinct.
18. Propodeal declivity convex in profile view.
19. Petiole reduced, without distinct dorsal node.
20. Abdominal sternite II without ventral process.
21. Abdominal tergite VIII distinctly longer than broad in posterodorsal view.
22. Abdominal sternite IX completely fused to gonocoxites.
23. Mulceators absent.
24. Cupula absent.
25. Gonopodites articulate.
26. Gonocoxites with complete ventromedian fusion.
27. Gonocoxites with complete dorsomedian fusion.
28. Gonocoxites completely fused to penial sclerites.
29. Gonostyli present.
30. Volsellae absent.
31. ***Inapplicable* .**
32. ***Inapplicable* .**
33. Penial sclerites with complete median fusion.
34. Penial sclerites not dorsoventrally compressed.
35. Phallotreme preapical.
36. Phallotreme dorsal.
37. Dense phallotremal vestiture of setae present or absent.

**Larval diagnosis.** Larva unknown.

**Remarks.** This clade is restricted to the Sundan region and the Philippines (Fig. 28). Most known specimens are Bornean in origin. The bizarre males of the *Leptanillahavilandi* species group were first described as the genera *Scyphodon* and *Noonilla*, with *Leptanillaanomala* (Brues, 1925) being regarded as Hymenoptera incertae sedis (Brues 1925). Male morphospecies attributable to *Noonilla* in addition to the type species (*L.copiosa*) were identified and sequenced by Griebenow (2020, 2021). Griebenow et al. (in press) treats this clade as *Scyphodon* s. l., despite not yet having subjected the position of *Scyphodon* relative to *Noonilla* to phylogenetic analysis. Nonetheless, Bayesian total-evidence inference confirms the monophyly of *Scyphodon* s. l. inclusive of *L.havilandi* (pers. obs.), here formally synonymized with *Leptanilla*.

The worker of *L.havilandi* bears a striking resemblance to *L.thai*, including in the presence of an emarginate frontoclypeal process, but is distantly related, demonstrating the morphological conservatism of the worker caste in *Leptanilla*. *Leptanillaclypeata* and *L.hypodracos* are sympatric with the *Leptanillahavilandi* species group, and morphologically like *L.havilandi*, introducing the possibility that these are members of this clade. Given the lack of phylogenetic signal in leptanilline worker morphology, however, this hypothesis must be tested with molecular data.

The close affinity of *L.anomala* and *L.copiosa*, to the exclusion of other described Leptanillinae, was not suggested by previous authors who argued for the placement of *L.anomala* within the Leptanillinae (Petersen 1968; Boudinot 2015). This is in part due to the preservation in balsam of the type series of *L.anomala*, a status that conceals autapomorphies of the *Leptanillahavilandi* species group, namely phallotremal setae and the distal transverse carina on the procoxa: examination of CASENT0106168 revealed these character states. In addition, the discovery of additional undescribed male morphospecies within the *Leptanillahavilandi* species group (Griebenow 2020, 2021; Griebenow et al. 2022) revealed intermediates in morphospace, juxtaposing the dorsoventrally compressed head and mesosoma of *L.anomala* with the nub-like, non-spatulate mandibles of *L.copiosa*.

### ﻿*Leptanillabethyloides* species group

**Worker diagnosis.** Worker unknown.

**Gyne diagnosis.** Gyne unknown.

**Male diagnosis.**

1. Mandalus ≥ 0.5× length of the mandible.
2. Mandible never fused to cranium, fully articulated.
3. Anteromedian ocellus posterior to compound eye.
4. LF2 < SL.
5. Distal transverse carina absent from procoxa.
6. Protrochanter not elongated.
7. Profemur not enlarged.
8. Arcuate medial carina absent from profemur.
9. Apicoventral hook absent from profemur.
10. Ventromedian carina absent from protibia.
11. Protibial comb absent.
12. Antero-admedian signum absent.
13. Pronotum and mesoscutum anteroposteriorly prolonged.
14. Mesoscutellum with or without recurved process.
15. Adventitious spectral M+Cu absent from forewing, or present (*Leptanilla* TH01).
16. Upper metapleuron distinct from metapectal-propodeal complex or indistinct.
17. Lower metapleuron distinct from metapectal-propodeal complex or indistinct.
18. Propodeal declivity convex in profile view.
19. Petiole well-developed, with or rarely without distinct dorsal node (*Leptanilla* TH07).
20. Abdominal sternite II with or without ventral process.
21. Abdominal tergite VIII broader than long in posterodorsal view.
22. Abdominal sternite IX posteriorly separate from gonocoxites.
23. Mulceators absent.
24. Cupula present (Griebenow et al. in press).
25. Gonopodites articulate.
26. Gonocoxites without ventromedian fusion.
27. Gonocoxites without complete dorsomedian fusion.
28. Gonocoxites unfused to penial sclerites.
29. Gonostyli present.
30. Volsellae absent.
31. ***Inapplicable* .**
32. ***Inapplicable* .**
33. Penial sclerites with complete median fusion.
34. Penial sclerites dorsoventrally compressed.
35. Phallotreme apical.
36. Dense phallotremal vestiture of setae absent.

**Larval diagnosis.** Larva unknown.

**Figure 30.**
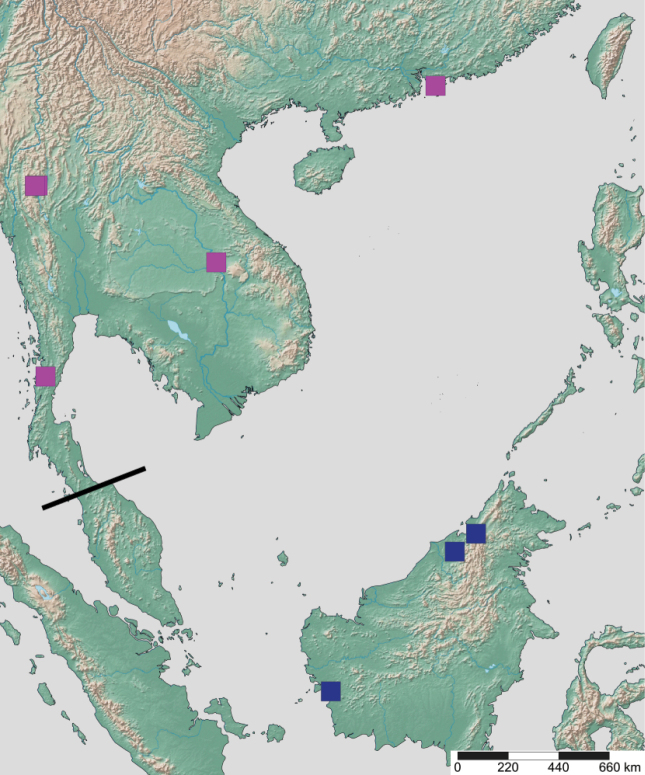
Geographical range of the *Leptanillabethyloides* species group (pink) and the *Leptanillanajaphalla* species group (blue). Pattani-Kangar Line indicated in black. Locality information derived from AntWeb and visualized with SimpleMappr.

**Remarks.** This species group is restricted to mainland Southeast Asia north of the Pattani-Kangar Line (Fig. 30), with the type locality of *L.bethyloides* being their northernmost known extent. Like the *Leptanillanajaphalla* species group, the *Leptanillabethyloides* species group is known only from male specimens. These are never abundant in known collections, with it therefore appearing that this species group exhibits genuine rather than artifactual rarity; no exemplars of this clade were described in detail by Griebenow et al. (in press), meaning that the male genital skeletomusculature of the *Leptanillabethyloides* species group is more poorly understood than that of any other major leptanilline clade.

Volsellae are completely absent in *Leptanilla* zhg-mm03 (CASENT0842829), in a homoplasy with the *Leptanillahavilandi* species group (Griebenow et al. in press). The total absence, as opposed to extreme reduction, of the volsellae cannot yet be definitively confirmed for any other representatives of the *Leptanillabethyloides* species group due to a lack of specimens for study.

The *Leptanillabethyloides* species group qualitatively possesses male morphological diversity disproportionate to the depauperation of known lineages: the condition of the metapleuron varies from completely indiscernible (*Leptanilla* TH07) to both the upper and lower metapleuron being completely visible (e.g., *L.bethyloides*). However, the lower metapleuron is never distinct from the metapectal-propodeal complex in the absence of the same distinction for the upper metapleuron, as in most of the *Leptanillahavilandi* species group. Other conditions unusual among *Leptanilla* that are sporadically observed in the *Leptanillabethyloides* species group include elongated antennomeres, a posteriorly recurved mesoscutellum (both only observed in *Leptanilla* zhg-th01), and a dorsomedian penial carina (*Leptanilla* TH01).

### ﻿*Leptanillanajaphalla* species group

**Worker diagnosis.** Worker unknown.

**Gyne diagnosis.** Gyne unknown.

**Male diagnosis.**

1. Mandalus ≥ 0.5× length of the mandible.
2. Mandible never fused to cranium, fully articulated.
3. Anteromedian ocellus posterior to compound eye.
4. LF2 < SL.
5. Distal transverse carina absent from procoxa.
6. Protrochanter not elongated.
7. Profemur enlarged, sometimes markedly constricted proximally.
8. Arcuate medial carina absent from profemur.
9. Apicoventral hook present or absent from profemur.
10. Ventromedian carina absent from protibia.
11. Protibial comb present.
12. Antero-admedian signum absent.
13. Pronotum and mesoscutum anteroposteriorly prolonged.
14. Mesoscutellum without recurved posterodorsal process.
15. Adventitious spectral M+Cu present in forewing.
16. Upper metapleuron indistinct from metapectal-propodeal complex.
17. Lower metapleuron indistinct from metapectal-propodeal complex.
18. Propodeal declivity convex in profile view, with distinct dorsal and posterior faces, dorsal face parallel to craniocaudal axis.
19. Petiole well-developed, with distinct dorsal node.
20. Abdominal sternite II with or without ventral process.
21. Abdominal tergite VIII broader than long in posterodorsal view.
22. Abdominal sternite IX with narrow posteromedian fusion to gonocoxites.
23. Mulceators present.
24. Cupula absent or present (*Leptanilla* zhg-id01), if present then fused anteriorly to abdominal sternite IX and posteriorly to gonocoxites (Griebenow et al. in press).
25. Gonopodites inarticulate.
26. Gonocoxites with complete dorsomedian fusion.
27. Gonocoxites with complete ventromedian fusion.
28. Gonocoxites fused to penial sclerites or unfused.
29. Gonostyli present or absent.
30. Volsellae present.
31. Volsellae medially fused.
32. Volsella never furcated, although paired, recurved cuticular processes may be present at apex.
33. Penial sclerites with complete median fusion.
34. Penial sclerites lateromedially compressed or subcircular in cross-section.
35. Phallotreme apical or subapical.
36. Phallotreme dorsal or ventral.
37. Dense phallotremal vestiture of setae absent.

**Larval diagnosis.** Larva unknown.

**Remarks.** This clade remains known only from males, necessitating the regrettable description of a species based solely upon male material (*L.najaphalla*) to provide the “Bornean morphospecies group” (Griebenow 2020, 2021) with an informal species group name. The males of the *Leptanillanajaphalla* species group are flagrantly bizarre, defined by such autapomorphies as a protibial comb composed of parallel-sided cuticular processes (previously misidentified as setae; Griebenow 2020, 2021), the complete median fusion of the volsellae at the base, and the presence of mulceators. It appears that the protibial comb is serially homologous with the probasitarsal comb, a structure synapomorphic for the Hymenoptera (Basibuyuk and Quicke 1995). While the protibial comb and mulceators are unparalleled in the Hymenoptera, the medial fusion of the volsellae is also observed in *Sceliphroncaementarium* (Drury, 1773) (Sphecidae: Sceliphrini) (Schulmeister 2003: fig. 11C).

Micro-CT scans reveal that all 7 morphospecies sampled in Griebenow et al. (in press) (including *L.najaphalla*, as *Leptanilla* zhg-my02) show posteromedian fusion of abdominal sternite IX to the gonocoxites, an apomorphy apparently derived independently from the anatomical condition observed in the *Leptanillahavilandi* species group (Griebenow et al. in press). This species group is robustly supported as sister to the *Leptanillahavilandi* species group (Griebenow 2020, 2021; Griebenow et al. 2022), which likewise is restricted to the Sundan region. Despite this phylogenetic position, no unequivocal male morphological synapomorphies are known for the two clades, with the fusion of S9 to the gonocoxites, and medial fusion of the gonocoxites, being perhaps homoplasious between the two according, given a lack of the Remanean homology criterion of “special quality” (Griebenow et al. in press). Further Winkler and pitfall sampling in the Sundan region, particularly Borneo, will be required to collect the unknown female castes of the *Leptanillanajaphalla* species group. It is also possible that *Leptanillabutteli* Forel, 1913 and *Leptanillakebunraya* Yamane & Ito, 2001, the worker morphology of which is aberrant among *Leptanilla*, are representatives of this clade.

### ﻿*Leptanillarevelierii* species group

**Worker diagnosis.**

1. Mandible with 3–4 teeth.
2. Maxillary palpomere 1-merous.
3. Frontoclypeal process absent or present, never emarginate.
4. Lateral clypeal teeth absent.
5. Meso-metapleural suture absent or present (*Leptanillahunanensis*).
6. Mesotibia with 0–1 spur.
7. Metatibia with two spurs.
8. Length of abdominal segment II subequal to width in dorsal view.
9. Anterior of abdominal tergite IV not lateromedially constricted in dorsal view.
10. Length of abdominal tergite IV equal or less than combined length of posterior abdominal tergites in dorsal view.

**Gyne diagnosis.** As for the genus, but petiole quadrate to distinctly broader than long in dorsal view.

**Male diagnosis.**

**Figure 31.**
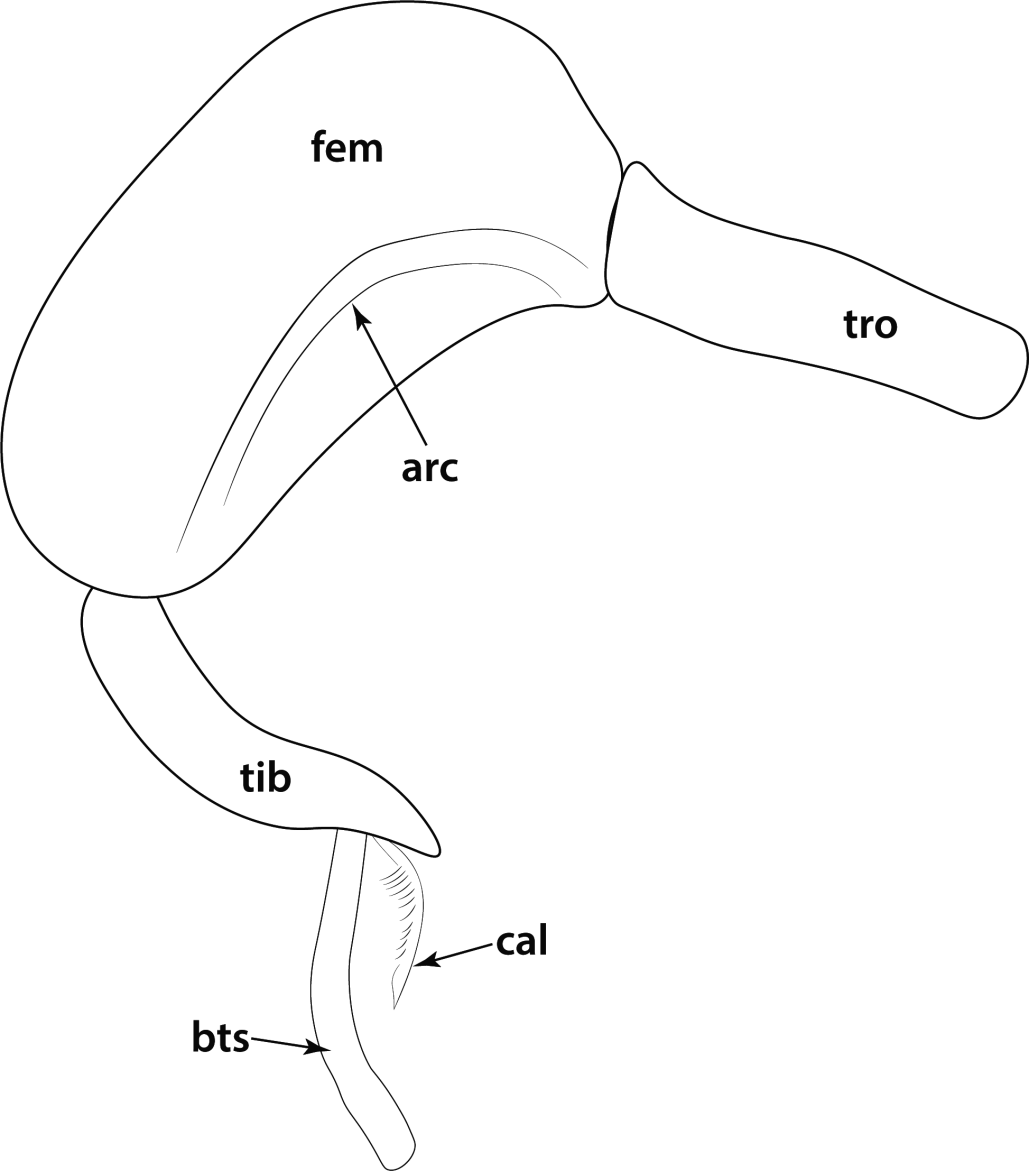
Foreleg of *Leptanilla* ci01, medial view, diagrammatic. Abbreviations: arc = arcuate medial carina; bts = probasitarsus; cal = calcar; fem = profemur; tib = protibia; tro = protrochanter.

1. Mandalus ≥ 0.5× length of the mandible.
2. Mandible never fused to cranium, fully articulated.
3. Anteromedian ocellus posterior to compound eye.
4. LF2 < SL.
5. Distal transverse carina absent from procoxa.
6. Protrochanter rarely elongated (*Leptanilla* ci01) (Fig. 31) or not elongated.
7. Profemur enlarged or not enlarged.
8. Arcuate medial carina present on profemur (*Leptanilla* ci01) (Fig. 31) or absent from profemur.
9. Apicoventral hook absent from profemur.
10. Ventromedian carina absent from protibia.
11. Protibial comb absent.
12. Antero-admedian signum absent.
13. Pronotum and mesoscutum anteroposteriorly prolonged.
14. Mesoscutellum without recurved posterodorsal process.
15. Adventitious spectral M+Cu absent from forewing.
16. Upper metapleuron indistinct from metapectal-propodeal complex.
17. Lower metapleuron indistinct from metapectal-propodeal complex.
18. Propodeal declivity convex in profile view.
19. Petiole well-developed, with or without distinct dorsal node.
20. Abdominal sternite II with or without ventral process.
21. Abdominal tergite VIII broader than long in posterodorsal view or rarely longer than broad in posterodorsal view (*Leptanilla* ci01).
22. Abdominal sternite IX posteriorly separate from gonocoxites.
23. Mulceators absent.
24. Cupula absent or present (*L.astylina*).
25. Gonopodites articulate, rarely inarticulate (*Leptanillaexigua* Santschi, 1908).
26. Gonocoxites with ventromedian fusion partial to complete (*L.astylina*).
27. Gonocoxites without complete dorsomedian fusion.
28. Gonocoxites unfused to penial sclerites.
29. Gonostyli present.
30. Volsellae present.
31. Volsellae medially separate.
32. Volsella entire.
33. Penial sclerites with complete median fusion.
34. Penial sclerites dorsoventrally compressed, rarely lateromedially compressed (*L.astylina*, *Leptanilla* zhg-na01).
35. Phallotreme apical or subapical.
36. Phallotreme dorsal.
37. Dense phallotremal vestiture of setae absent.

**Larval diagnosis.** As for genus.

**Figure 32.**
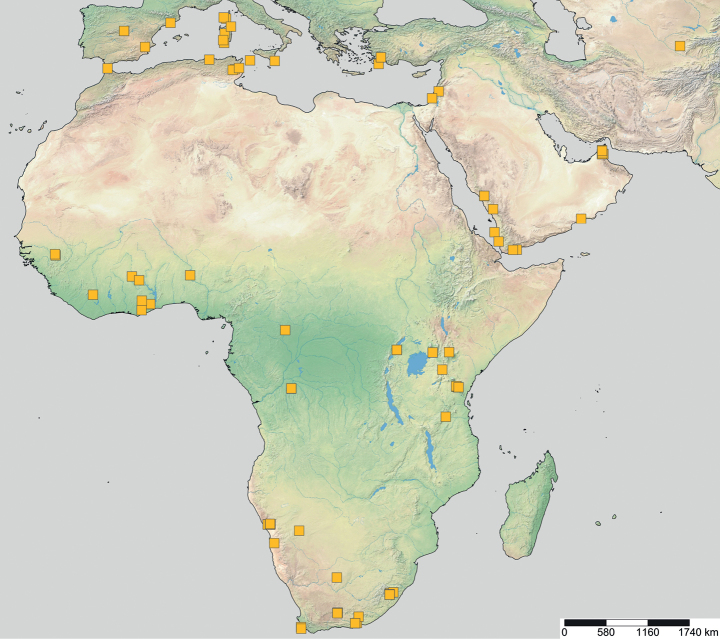
Geographical range of the *Leptanillarevelierii* species group in the Western Palaearctic and Afrotropics. Locality information derived from AntWeb and available literature, visualized with SimpleMappr.

**Figure 33.**
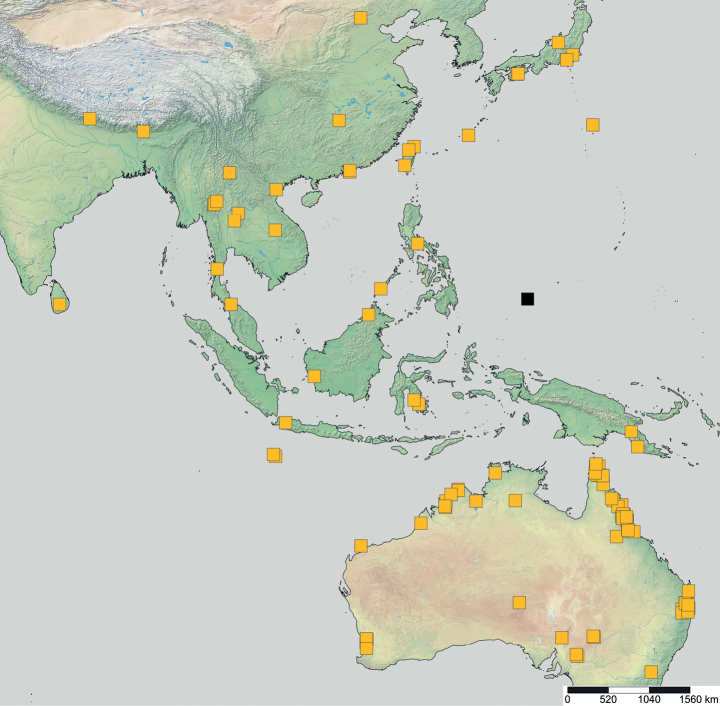
Geographical range of the *Leptanillarevelierii* species group (yellow) and *Leptanillapalauensis* (black) in the Eastern Palaearctic, Indo-Malaya, and Australasia. Locality information derived from AntWeb and available literature, visualized with SimpleMappr.

**Remarks.** The *Leptanillarevelierii* species group is by far the most geographically widespread clade within the Leptanillinae and correspondingly is the most speciose. *Leptanillarevelierii* Emery was the first species within the Leptanillinae to be scientifically described, while *Leptanillajaponica* Baroni Urbani is the leptanilline species that has been subjected to the most bionomic study. This is the only leptanilline clade to have expanded its range west of the Arabian subcontinent, radiating extensively throughout the Afrotropics and the Mediterranean Basin (Fig. 32). It does not appear that this species group extends into temperate latitudes of the Western Palaearctic, but *Leptanillaalexandri* Dlussky, 1969 is reported from Uzbekistan (Dlussky 1969). The *Leptanillarevelierii* species group, with the *Protanillarafflesi* species group, are the sole leptanilline clades confirmed to range into the Eastern Palaearctic and occupy fully temperate climates (Fig. 33). In addition, the *Leptanillarevelierii* species group is so far the only clade within the Leptanillinae known to have traversed Wallace’s Line. The apparent ease with which this clade has radiated across the Old World is striking when compared to its sister, which remains restricted to only a portion of the Indo-Malayan ecoregion.

*Leptanillaswani* Wheeler is the sole species of *Leptanilla* to be described from Australia, although the undescribed species-level diversity of *Leptanilla* from that continent is conspicuous, with richness highest in Queensland. Male specimens are known from as far south as the Australian Capital Territory. *Leptanilla* zhg-au06 is known from a single male specimen collected on Christmas Island, in what may be a human-mediated introduction. Contrary to the suggestion of Wheeler (1932) that *Leptanilla* are relict elements of the Australian ant fauna, the *Leptanillarevelierii* species group can be assumed to be recent arrivals to Australasia from the Indo-Malayan ecoregion. There is also a great undescribed diversity of the *Leptanillarevelierii* species group in the Afrotropics, with no fewer than nine male morphospecies purportedly being collected at the Brandberg Massif in Namibia (Robertson 2000). Malaise trapping in conjunction with syntopic soil sampling in the Afrotropics and Australasia will surely yield a large trove of new species belonging to the *Leptanillarevelierii* species group. Collections of the *Leptanillarevelierii* species group in the Indo-Malayan ecoregion remain scanty compared to sympatric members of other species groups of *Leptanilla*.

*Leptanilla* ci01 is here provisionally considered to belong to the *Leptanillarevelierii* species group, despite its extreme deviation from the male morphology observed in the rest of that clade, since (1) Bayesian total-evidence inference excludes this aberrant morphospecies from all other major *Leptanilla* clades with posterior probability greater than 0.95 (pers. obs.) and (2) no other clade of *Leptanilla* is known to exist in sub-Saharan Africa. Bayesian total-evidence inference likewise excludes *L.astylina* from all clades within the Leptanillinae besides the *Leptanillarevelierii* species group, with high posterior probability (pers. obs.). What were interpreted as “medially fused volsellar plates” by Petersen (1968: 581) appear in fact to be the gonocoxites, with the “large, valve-like” sclerites interpreted as the gonocoxites (Petersen 1968: 581) therefore corresponding to the gonostyli—the putative absence of gonostyli referred to by the specific epithet of *L.astylina* is therefore false. Even with this reinterpretation, the male genitalia in *L.astylina* deviate from what is observed in the rest of the *Leptanillarevelierii* species group, conspicuously in the presence of a cupula (Ogata et al. 1995), complete ventromedian fusion of the gonocoxites and the medial separation of the penial sclerites shown in Petersen (1968: figs 3, 4), which could not be confirmed by examination of the holotype. The medial concavity and ellipsoid outline of the gonostylus (Petersen 1968: fig. 3) is also aberrant among the *Leptanillarevelierii* species group, as is the lateral concealment of the gonocoxite by the gonostylus (Petersen 1968: fig. 5) and the exposure of the volsellae. *Leptanillaastylina* may be sister to the remainder of the *Leptanillarevelierii* species group.

Despite the variety and vast geographical range of the *Leptanillarevelierii* species group, male morphology within the clade is quite homogeneous relative to the other major subclades of *Leptanilla* for which males are known, particularly when compared to the species-poor *Leptanillahavilandi* and *Leptanillanajaphalla* species groups. The dramatic innovation observed across the male phenotype of *Leptanilla* ci01 is striking when considered in this context.

### ﻿Incertae sedis

Molecular data are unavailable for these species of *Leptanilla*; even with the contextualization of leptanilline morphology onto a well-resolved phylogeny inferred from molecular data or jointly from those data and discretized male morphology (Griebenow 2021), these species cannot be confidently placed to the species groups delimited here, due to morphological evidence that is equivocal in phylogenetic signal or too aberrant to be of comparative use. *Leptanillaclypeata* Yamane & Ito, 2001 is known from both the worker and gyne; *Leptanillapalauensis* (M.R. Smith, 1953) from the male alone; and the remaining species only from the worker caste. Most of these morphospecies are known only from the Indo-Malayan ecoregion.

*Leptanillaclypeata* and *L.hypodracos* are very similar to one another, and closely conform to the worker-based diagnosis of the sympatric *Leptanillahavilandi* species group and the parapatric *Leptanillathai* species group. The palpal formulae of these species would provide further evidence as to their phylogenetic position, but have not been described, and I was not able to obtain specimens for study. These species differ from the *Leptanillahavilandi* and *thai* species groups only in the emargination of the anterior petiolar margin in dorsal view. Worker morphology is quite invariable across *Leptanilla*, and so the phylogenetic significance of this character state cannot be extrapolated; given the relative morphological conformity of the worker caste between the phylogenetically distant *L.havilandi* and *L.thai*, even the phylogenetic affinity of *L.clypeata* and *L.hypodracos* with one another cannot be assumed without corroboration.

*Leptanillabutteli* resembles the *Leptanillarevelierii* species group overall but differs from the members of that clade in having two mandibular teeth rather than three or four, and abdominal sternite II projecting distinctly below the level of abdominal sternite III along the dorsoventral axis (Baroni Urbani 1977: fig. 25). *Leptanillakebunraya* joins *L.butteli* in being one of the only two *Leptanilla* species in which the worker mandible has two teeth, but otherwise bears little apparent resemblance to *L.butteli* to the exclusion of other *Leptanilla*. *L.kebunraya* is unique among known *Leptanilla* in having anterolateral frontoclypeal projections, which invite comparison with the lateral clypeal teeth of *Feroponeraferox* Bolton & Fisher, 2008 (Ponerinae: Ponerini). This is of no help in inferring the function of these structures in *L.kebunraya* since the biology of *F.ferox* is largely unknown (Bolton and Fisher 2008).

*Leptanillapalauensis* was described as the first known male of *Probolomyrmex* Mayr (Proceratiinae: Probolomyrmecini), without associated workers or gynes (Smith 1953), and is still known only from the holotype. Taylor (1965) tentatively transferred the species to *Leptanilla*, with Petersen (1968) following this classification with some reservation, noting that William Brown and Edward O. Wilson doubted it was even an ant. Griebenow (2021) briefly mentioned *L.palauensis*, noting that examination of the holotype confirmed its placement within *Leptanilla* s. l. (Griebenow 2021: 628). This phylogenetic position is confirmed by Bayesian total-evidence inference (pers. obs.); however, the exact phylogenetic position of this morphospecies within *Leptanilla* remains poorly resolved, and the combination of character states observed in in *L.palauensis* excludes the species from all species groups of *Leptanilla* here delimited. The lateromedial compression of the penial sclerites, in conjunction with well-developed volsellae, perhaps implies a phylogenetic relation with the *Leptanillanajaphalla* species group, or with *Leptanilla* zhg-my08 (for which molecular data are unavailable), also incertae sedis; both these lineages are known only from Borneo. *L.palauensis* is a striking biogeographical outlier among the Leptanillinae, being known only from the volcanic island of Babeldaob in Palau, and therefore the only known leptanilline from Oceania (Fig. 33). All known *Leptanilla* gynes are flightless, limiting their dispersal capabilities, but the remote location of *L.palauensis* is paralleled by the presence of *Leptanillaoceanica* Baroni Urbani in the Ogasawara Islands (Baroni Urbani 1977).

Almost nothing is known of the biology of *Leptanillabutteli*, *L.kebunraya*, and *L.hypodracos*. Among *Leptanilla*, our biological knowledge of *L.clypeata* is second in comprehensiveness only to that available for *L.japonica*, with Ito and Yamane (2020) providing observations of live colonies, including feeding and egg-laying behavior. Billen et al. (2022) and Billen and Ito (2022) thoroughly described the exocrine glands of worker *L.clypeata*, with the dorsoproximal intramandibular gland discovered in this species being novel for the Formicidae.

### ﻿Unplaced to species group

Molecular data are unavailable for *Leptanillasantschii* Wheeler & Wheeler, 1930, which is known only from the male holotype. The club-like volsellae and absent gonostyli of *Leptanillasantschii* (Wheeler and Wheeler 1930: fig. 2D; Petersen 1968) would exclude this species from the *Leptanillarevelierii* species group, if the description of Wheeler and Wheeler (1930) is accurate, but with the holotype missing (Stefan Cover, pers. comm. 2020), morphological data are too limited to permit Bayesian total-evidence inference to test this hypothesis.

### ﻿Worker-based keys to the Leptanillinae

Most subclades of the Leptanillinae show strong morphological conservatism in the worker caste. It is consequently difficult to assess the scope of intraspecific phenotypic variation in workers, and the sparseness of collected specimens prevents algorithmic species delimitation using molecular data. Therefore, morphospecies known only from a single specimen are excluded from the following keys, even if phylogenomic data are available therefrom and no new species are described in this study based upon worker singletons. Any such species hypothesis would be weak due to lack of comparative context, and be falsifiable simply by the discovery of additional specimens (Bond et al. 2022).

**Table:** 

1	Abdominal segment III not petiolate (Fig. 34A); occiput visible in full-face view (Opamyrmini)	***Opamyrmahungvuong*Yamane et al., 2008 (VIETNAM: Ha Tinh, Son La; CHINA: Hainan, Guangxi)**
–	Abdominal segment III petiolate (Fig. 34B, C); occiput not visible in full-face view (Leptanillini)	**2**
2	Clypeus extending posteriorly between antennal toruli (Fig. 22A); epistomal sulcus present medially (*Protanilla*)	**3**
–	Clypeus not extending posteriorly between antennal toruli (Fig. 22B); epistomal sulcus indistinct medially (*Leptanilla*)	**6**
3	Abdominal tergite II without distinct posterior face (Fig. 34C); clypeus oblate-trapezoidal in full-face view; peg-like chaetae absent from mandible	***Protanillataylori* species group**
–	Abdominal tergite II with distinct posterior face (Fig. 34B); clypeus campaniform in full-face view; peg-like chaetae present on mandible	**4**
4	Clypeus oblate-trapezoidal in outline, elevated above frons posteriorly (Fig. 35A); mandible bowed along anteroposterior axis of cranium	***Protanillaizanagi* Terayama, 2013 (JAPAN: Honshu)**
–	Clypeus campaniform in outline (Fig. 1B), not elevated above frons posteriorly (Fig. 35B); mandible straight	**5**
5	Mesotibia with one spur; mandible without laterodorsal longitudinal groove; anterior margin of clypeus concave	***Protanillabicolor* species group**
–	Mesotibia without spurs; mandible with laterodorsal longitudinal groove; anterior margin of clypeus planar	***Protanillarafflesi* species group**
6	Anterior margin of cranium with median process	**7**
–	Anterior margin of cranium without median process	**10**
7	Frontoclypeal process entire; length of abdominal tergite IV usually less than combined length of abdominal tergites V–VII in dorsal view, sometimes subequal	***Leptanillarevelierii* species group (in part)**
–	Frontoclypeal process emarginate; length of abdominal tergite IV usually greater than combined length of abdominal tergites V–VII in dorsal view, sometimes subequal	**8**
8	Anterior margin of petiolar node entire in dorsal view (Leong et al. 2018: fig. 13A, D)	***Leptanillathai* species group, *Leptanillahavilandi* species group**
–	Anterior margin of petiolar node emarginate in dorsal view (Leong et al. 2018: fig. 13E, F)	**9**
9	In full-face view, mandible with most proximal tooth long and well-defined; petiolar node almost twice as long as wide in dorsal view; postpetiolar node longer than wide in dorsal view	***Leptanillahypodracos* Wong & Guénard, 2016 (SINGAPORE)**
–	In full-face view, mandible without most proximal tooth long and well-defined; length and width of petiolar node subequal in dorsal view; postpetiolar node distinctly wider than long in dorsal view	***Leptanillaclypeata* Yamane & Ito, 2001 (INDONESIA: Java)**
10	Mandible with 3–4 teeth	***Leptanillarevelierii* species group (in part)**
–	Mandible with 2 teeth	**11**
11	Anterior margin of cranium with anterolateral frontoclypeal projections; abdominal sternites II-III projecting a subequal distance ventrad craniocaudal axis	***Leptanillakebunraya* Yamane & Ito, 2001 (INDONESIA: Java)**
–	Anterior margin of cranium entire; abdominal sternite II projecting distinctly lower than abdominal sternite III	***Leptanillabutteli* Forel, 1913 (MALAYSIA: Selangor)**

### ﻿Worker-based key to the *Protanillataylori* species group

*Protanillataylori* comb. nov. and the undescribed *Protanilla* id01 are known only from the gyne, and thus excluded from this key. It does not appear that either *P.taylori* or *Protanilla* id01, which are known only from Borneo, represent the gyne of *P.boltoni* or *P.helenae* (Borowiec et al. 2011).

**Table:** 

1	Cranium, pronotum and mesopleuron puncticulate to roughly sculptured; subpetiolar process lacking fenestra in profile view	***Protanillaboltoni* (Borowiec et al., 2011), comb. nov. (MALAYSIA: Perak)**
–	Cranium, pronotum and mesopleuron glabrous; subpetiolar process with fenestra in profile view	***Protanillahelenae* (Borowiec et al., 2011), comb. nov. (PHILIPPINES: Palawan)**

### ﻿Worker-based key to the *Protanillabicolor* species group

**Table:** 

1	Cranium black-brown; anterior face of petiolar node sloping in profile view	***Protanillagengma* Xu, 2012 (CHINA: Yunnan; INDIA: Mizoram; VIETNAM: Dong Nai, Bac Giang, Ninh Binh)**
–	Cranium yellowish; anterior face of petiolar node subvertical in profile view	***Protanillabicolor* Xu, 2002 (CHINA: Yunnan)**

### ﻿Worker-based key to the *Protanillarafflesi* species group

*Protanillaschoedli* Baroni Urbani & de Andrade, 2006 is known only from the gyne (Baroni Urbani and de Andrade 2006) and is excluded from the key. Dias et al. (2019) described the putative worker; however, given known morphological variation in the worker caste among described species of *Protanilla*, I here consider this as representing an undescribed species, related to *Protanillaflamma* Baidya & Bagchi, 2020.

**Table:** 

1	Abdominal sternite III linear to slightly concave in profile view; abdominal segments III–IV broadly conjoined, with abdominal tergite III lacking a distinct posterior face	**2**
–	Abdominal sternite III convex in profile view; abdominal segments III–IV not broadly conjoined, with abdominal tergite III having a distinct posterior face	**3**
2	Anterior margin of abdominal tergite IV emarginate in dorsal view; two ventrolateral teeth present on mandible	***Protanillafurcomandibula* Xu & Zhang, 2002 (CHINA: Yunnan)**
–	Anterior margin of abdominal tergite IV entire in dorsal view; one ventrolateral tooth present on mandible	***Protanillajongi*Hsu et al., 2017 (TAIWAN)**
3	Anterior face of petiolar node concave in profile view	**4**
–	Anterior face of petiolar node linear in profile view	**5**
4	In profile view anterodorsal corner of petiolar node projecting anteriorly; larger species (WL > 0.8 mm)	***Protanillarafflesi* Taylor in Bolton, 1990 (SINGAPORE; MALAYSIA: Sabah, Sarawak)**
–	In profile view anterodorsal corner of petiolar node not projecting anteriorly; smaller species (WL 0.70–0.80 mm) (*n* = 2)	***Protanillawardi* Bharti & Akbar, 2015 (INDIA: Kerala)**
5	In dorsal view petiolar node breadth and length subequal; postpetiolar node not inclined anteriorly in profile view	**6**
–	In dorsal view petiolar node distinctly broader than long; postpetiolar node inclined anteriorly in profile view	**9**
6	Coloration castaneous (Fig. 22A); larger species (HL = 0.63–0.70 mm; WL = 0.99 mm) (*n* = 1)	***Protanillabeijingensis*Man et al., 2017 (CHINA: Beijing; PAKISTAN: Khyber Pakhtunkhwa)**
–	Coloration coppery or yellowish; smaller species (HL = 0.42–0.59 mm; WL = 0.64–0.94 mm) (*n* = 16)	**7**
7	Scape not extending beyond occipital vertex of cranium in full-face view (SI ≤ 90); coloration coppery	***Protanillaflamma* Baidya & Bagchi, 2020 (INDIA: Goa)**
–	Scape extending beyond occipital vertex of cranium in full-face view (SI > 90); coloration yellowish (Fig. 4A–C)	**8**
8	Larger species (WL ≥ 0.75 mm) (*n* = 2); postpetiolar node prominent in profile view, with anterior and posterior declivities equally rounded (Fig. 6A)	***Protanillalini* Terayama, 2009 (TAIWAN; JAPAN: Okinawa, Ryukyu Islands; Senkaku Islands)**
–	Smaller species (WL < 0.75 mm) (*n* = 14); postpetiolar node shallow in profile view, with posterior declivity more gradual than anterior declivity (Fig. 5B)	***Protanillawallacei* sp. nov. (MALAYSIA: Sabah, Selangor)**
9	Lateral margin of head with acute dorsal mandibular articulation in full-face view; anteroventral corner of sub-post-petiolar process obliquely truncated	***Protanillatibeta* Xu, 2012 (CHINA: Xizang)**
–	Lateral margin of head without dorsal mandibular articulation apparent in full-face view (Fig. 24A); anteroventral corner of sub-post-petiolar process rounded	**10**
10	Meso-metapleural furrow deeply excavated in profile view; very large species (HW = 0.82–0.84 mm) (*n* = 3) (Satria et al. 2023)	***Protanillaeguchii*Satria et al., 2023 (INDONESIA: Sumatra)**
–	Meso-metapleural furrow shallowly excavated in profile view; smaller species (HW = 0.48 mm) (*n* = 1)	***Protanillaconcolor* Xu, 2002 (CHINA: Yunnan)**

### ﻿Worker-based key to the *Leptanillathai* species group and *Leptanillahavilandi* species group

**Table:** 

1	SI > 100; length of petiole > 3× greater than maximum breadth in dorsal view (Griebenow et al. 2022: fig. 6B)	***Leptanillalaventa* (Griebenow et al., 2022), comb. nov. (IRAN: Fārs)**
–	SI ≤ 100; length of petiole ≤ 3× greater than maximum breadth in dorsal view (Fig. 6A)	**2**
2	Length of metasomal setae bimodal	**3**
−	Length of metasomal setae unimodal	**5**
3	Mandible with four teeth, with most proximal tooth truncate (Saroj et al. 2022: fig. 1E); ventromedian lamella of abdominal sternite II denticulate	***Leptanillaujjalai*Saroj et al., 2022 (INDIA: West Bengal)**
–	Mandible with three teeth, with most proximal tooth not truncate; ventromedian lamella of abdominal sternite II not denticulate	**4**
4	Lateral pronotal margins weakly convex in dorsal view; PPTI = 73.68–76.47 (*n* = 11)	***Leptanillalamellata* Bharti & Kumar, 2012 (INDIA: Himachal Pradesh)**
−	Lateral pronotal margins strongly convex in dorsal view; PPTI = 84.62–85.71 (*n* = 6)	***Leptanillaescheri* (Kutter, 1948) (INDIA: Tamil Nadu)**
5	Petiolar length ≥ 2× width	**6**
–	Petiolar length ≤ 1.5× width	**8**
6	Meso-metapleural furrow absent; mandible with four teeth, most proximal tooth distally recurved, apex expanded	***Leptanillabelantan* sp. nov. / (MALAYSIA: Selangor)**
–	Meso-metapleural furrow present; mandible with three teeth, most proximal tooth acute	**7**
7	Abdominal sternite III no more anteroposteriorly compressed than abdominal tergite III	***Leptanillakunmingensis* Xu & Zhang, 2002 (CHINA: Yunnan)**
–	Abdominal sternite III more anteroposteriorly compressed than abdominal tergite III	***Leptanillajudaica* Kugler, 1987 (WEST BANK)**
8	Subpetiolar process present, angular; torulus without areolate sculpture (Fig. 27B)	***Leptanillahavilandi* Forel, 1901 (SINGAPORE; MALAYSIA: Sabah)**
–	Subpetiolar process absent; torulus with medial and anterior areolate sculpture (Fig. 27A)	***Leptanillathai* Baroni Urbani, 1977 (THAILAND: Khao Chong)**

### ﻿Worker-based key to the *Leptanillarevelierii* species group

**Table:** 

1	Anterior margin of cranium with median process	**2**
–	Anterior margin of cranium without median process	**4**
2	Mandible with four teeth	***Leptanillaboltoni* Baroni Urbani, 1977 (GHANA)**
–	Mandible with three teeth	**3**
3	Posteriorly recurved subpetiolar process present; PPI = 122–138 (*n* = 5)	***Leptanillamacauensis*Leong et al., 2018 (CHINA: Macau)**
–	Posteriorly recurved subpetiolar process absent; PPI = 80–86 (*n* = 2)	***Leptanillabuddhista* Baroni Urbani, 1977 (NEPAL)**
4	Meso-metapleural groove present, impressed on dorsum of mesosoma	***Leptanillahunanensis* Tang et al., 1992 (CHINA: Hubei, Hunan, Yunnan)**
–	Meso-metapleural groove absent from dorsum of mesosoma, sometimes faintly impressed on sides	**5**
5	Anterior margin of cranium with median emargination	**6**
–	Anterior margin of cranium entire, linear to convex	**9**
6	Four mandibular teeth; greatest width of petiolar node in dorsal view distinctly posterior to midlength	***Leptanillavaucheri* Emery, 1899 (MOROCCO)**
–	Three mandibular teeth; greatest width of petiolar node in dorsal view not distinctly posterior to midlength	**7**
7	Length of abdominal segment II subequal to that of abdominal segment III in dorsal view; abdominal tergite IV narrowed anteriorly in dorsal view (Fig. 36A)	***Leptanillataiwanensis*Ogata et al., 1995 (TAIWAN; CHINA: Beijing)**
–	Abdominal segment II longer than abdominal segment III in dorsal view; abdominal tergite IV not narrowed anteriorly in dorsal view (Fig. 36B)	**8**
8	Outline of abdominal segment III campaniform in dorsal view; frontoclypeal margin convex	***Leptanillaoceanica* Baroni Urbani, 1977 (JAPAN: Ogasawara Islands)**
–	Outline of abdominal segment III subrectangular in dorsal view; frontoclypeal margin linear	***Leptanillaswani* Wheeler, 1932 (AUSTRALIA: Western Australia)**
9	Mandible with four teeth (subapical tooth sometimes difficult to distinguish)	**10**
–	Mandible with three teeth	**18**
10	Propodeum angular in profile view, with distinct posterior and dorsal faces	***Leptanillaortunoi*López et al., 1994 (SPAIN: Ceuta)**
–	Propodeum rounded in profile view, without distinct posterior and dorsal faces	**11**
11	Abdominal sternite II emarginate in profile view, with narrow trough-like indentation (Fig. 37A)	***Leptanillapoggii* Mei, 1995 (ITALY: Pantellaria)**
–	Abdominal sternite II linear in profile view (Fig. 37B)	**12**
12	Frontal margin of cranium convex in full-face view; scape strongly constricted at base	***Leptanillanana* Santschi, 1915 (TUNISIA)**
–	Frontal margin of cranium linear in full-face view; scape moderately constricted at base	**13**
13	Abdominal sternite II with planar face in profile view	**14**
–	Abdominal sternite II with rounded face in profile view	**15**
14	Most proximal mandibular tooth large and distinct; abdominal tergite IV distinctly narrowed anteriorly in dorsal view	***Leptanillatanakai* Baroni Urbani, 1977 (JAPAN: Yakushima)**
–	Most proximal mandibular tooth small and indistinct; abdominal tergite IV not distinctly narrowed anteriorly in dorsal view	***Leptanillajaponica* Baroni Urbani, 1977 (JAPAN: Honshu, CHINA: Hong Kong)**
15	Height of metafemur in anterior view 0.5× metafemoral length in anterior view; coloration beige	***Leptanillacharonea*Barandica et al., 1994 (SPAIN)**
–	Height of metafemur in anterior view < 0.5× of metafemoral length in anterior view; coloration yellowish	**16**
16	Larger species (HL = 0.32–0.36 mm)	**17**
–	Smaller species (HL = 0.22–0.28 mm) (López et al. 1994)	***Leptanillazaballosi*Barandica et al., 1994 (SPAIN)**
17	PI = 66–77 (Pérez-González et al. 2020)	***Leptanillaplutonia*López et al., 1994 (SPAIN)**
–	PI = 84.6–100 (Pérez-González et al. 2020)	***Leptanillatheryi* Forel, 1903 (ALGERIA; TUNISIA; SPAIN)**
18	Abdominal sternite II sinuate in profile view	***Leptanilladoderoi* Emery, 1915 (ITALY: Sardinia)**
–	Abdominal sternite II linear to convex in profile view, never sinuate	**19**
19	Petiole distinctly wider than long	***Leptanillayunnanensis* Xu, 2002 (CHINA: Yunnan)**
–	Petiole not distinctly wider than long	**20**
20	Frontal margin convex in full-face view	**21**
–	Frontal margin linear in full-face view	**22**
21	Mesothorax anteriorly constricted in dorsal view	***Leptanillabesucheti* Baroni Urbani, 1977 (SRI LANKA)**
–	Mesothorax not anteriorly constricted in dorsal view	***Leptanillamorimotoi* Yasumatsu, 1960 (JAPAN: Kyushu)**
22	Length of abdominal tergite V > 0.5× length of abdominal tergite IV	***Leptanillarevelierii* Emery, 1870 (FRANCE: Corsica; ITALY: Sardinia; SPAIN; PORTUGAL; MOROCCO)**
–	Length of abdominal tergite V ≤ 0.5× length of abdominal tergite IV	**23**
23	Pedicel distinctly longer than wide; abdominal sternite II linear in profile view	***Leptanillakubotai* Baroni Urbani, 1977 (JAPAN: Shikoku)**
–	Pedicel length and width subequal; abdominal sternite II convex in profile view	**24**
24	Smaller species (WL < 0.3 mm)	***Leptanillaokinawensis* Terayama, 2013 (JAPAN: Okinawa)**
–	Larger species (WL ≥ 0.3 mm)	***Leptanillaacherontia* sp. nov. (KENYA; UGANDA)**

### ﻿Male-based key to the major subclades of the Leptanillinae

The following keys are corrected and extended from Griebenow (2020), with updated generic assignments for undescribed morphospecies; concordances of these morphospecies identifiers with previous publications are provided in Table 1. Respective male-based keys to each of the major subclades are subsequently provided.

These include all described species for which males are known, and all undescribed male morphospecies for which molecular data are or soon will be available, except for *Leptanilla* ZA01 (for which only genital morphology is known), *Leptanilla* TH07 and *Leptanilla* zhg-mm14 (for which genital morphology is unknown). Based on phylogenetic inference from both molecular and morphological data (Griebenow 2021; pers. obs.), these three morphospecies belong to the *Leptanillarevelierii* species group, the *Leptanillabethyloides* species group, and the *Leptanillathai* species group, respectively. *Leptanilla* zhg-au04 and zhg-au06, of the *Leptanillarevelierii* species group, are also excluded due to lacking observations of the gonopodital apex, making it impracticable to include these morphospecies in the male-based key to that clade.

**Table:** 

1	Rs+M and *1m-cu* present (Fig. 38A); parossiculus (=cuspis in part) and lateropenite (=digitus) distinct, articulated (Opamyrmini)	***Opamyrmahungvuong*Yamane et al., 2008 (VIETNAM: Ha Tinh, Son La; CHINA: Hainan, Guangxi)**
–	Rs+M and *1m-cu* absent (Fig. 38B–D); if volsella discernible, parossiculus and lateropenite distinct or indistinct, if distinct then inarticulate (Leptanillini)	**2**
2	Pterostigma present (Fig. 39B); ocelli present, with ocellar tubercle absent (Fig. 40A); parossiculus and lateropenite distinct (*Protanilla*)	**3**
–	Pterostigma absent (Fig. 39A, C); ocelli present or absent, if present then set on ocellar tubercle (Fig. 40B, C), tubercle rarely absent (e.g., *Leptanillanajaphalla* sp. nov.); parossiculus and lateropenite not distinct (*Leptanilla*)	**5**
3	MaL < 0.5× ML; apex of mandible acuminate	***Protanilla* zhg-th02 (THAILAND: Chaiyaphum)**
–	ML ≥ 0.5× ML; apex of mandible rounded	**4**
4	Abdominal segment III petiolate; abdominal segment IV equal in length to combined length of abdominal segments V–VIII (*Protanillabicolor* species group)	***Protanilla* TH03 (THAILAND: Chiang Mai)**
–	Abdominal segment III not petiolate; length of abdominal segment IV subequal to, or less than, respective lengths of abdominal segments V–VII	***Protanillarafflesi* species group**
5	Propodeum concave in profile view (Fig. 41A); anteromedian ocellus directly dorsal to compound eye in profile view; pronotum and mesoscutum not posteriorly prolonged	***Leptanillathai* species group**
–	Propodeum not concave in profile view (Fig. 41B, C); anteromedian ocellus posterad compound eye in profile view, rarely directly dorsal (*Leptanillacopiosa* (Petersen, 1968), comb. nov.); pronotum and mesoscutum posteriorly prolonged	**6**
6	Propodeum with lateral longitudinal carinae on dorsum; penial sclerites lateromedially compressed	***Leptanillapalauensis* (M.R. Smith, 1953) (PALAU)**
–	Propodeum without lateral longitudinal carinae on dorsum; penial sclerites sometimes lateromedially compressed, more often not	**7**
7	Dorsal propodeal face long, parallel to craniocaudal axis (Fig. 41B); mulceators present; protibial comb present (Fig. 42A)	***Leptanillanajaphalla* species group**
–	Dorsal propodeal face short, with propodeal outline in profile view convex, if long and parallel to craniocaudal axis then upper metapleuron distinct from metapectal-propodeal complex; mulceators absent; protibial comb absent (Fig. 42B)	**8**
8	Procoxa with distal transverse carina (Fig. 29A); phallotreme surrounded with decumbent setae, rarely bare (*Leptanilla* zhg-ph01); if lower metapleuron distinct from metapectal-propodeal complex then upper metapleuron not distinct	***Leptanillahavilandi* species group**
–	Procoxa without distal transverse carina (Fig. 29B); phallotreme bare; lower metapleuron usually indistinct from metapectal-propodeal complex, if distinct then upper metapleuron distinct	**9**
9	Metapleuron at least partly distinct; vestiture dense and pubescent; volsellae apparently absent	***Leptanillabethyloides* species group**
–	Metapleuron never distinct; vestiture rarely dense, never pubescent; volsellae present	**10**
10	Gonostylus absent; volsella distally expanded; Sc+R+Rs and Rf1 nebulous, *2s-rs*+Rsf4-6 absent	***Leptanillasantschii* Wheeler & Wheeler, 1930 (INDONESIA: Java)**
–	Gonostylus present, articulated to gonocoxite, rarely inarticulate (*Leptanillaexigua* Santschi, 1908); volsella never distally expanded; Sc+R+Rs and Rf1 present or rarely absent, *2s-rs*+Rsf4-6 present or absent	***Leptanillarevelierii* species group**

### ﻿Male-based species-level key to the *Protanillarafflesi* species group

**Table:** 

1	Antero-admedian signum present	***Protanilla* TH02 (THAILAND: Chaiyaphum)**
–	Antero-admedian signum absent	**2**
2	Gonostylar apex pointed (Fig. 43A)	***Protanilla* TH01 (THAILAND: Khon Kaen)**
–	Gonostylar apex rounded (Fig. 43B)	**7**
3	Anterior face of subpetiolar process nearly perpendicular to craniocaudal axis in profile view; abdominal tergite III slightly narrower than IV in dorsal view (TI1 62–92) (*n* = 13) (Fig. 44A)	***Protanilla* zhg-vn01 (VIETNAM: Vinh Phuc)** ***Protanilla* zhg-my01 (MALAYSIA: Sarawak) ^1^**
–	Anterior face of subpetiolar process gently sloping relative to craniocaudal axis; abdominal tergite III much narrower than IV in dorsal view (TI1 50–55) (*n* = 4) (Fig. 44B)	***Protanillalini* Terayama, 2009 (TAIWAN; JAPAN: Ryukyu Islands, Senkaku Islands)**

### ﻿Male-based species-level key to the *Leptanillathai* species group

**Table:** 

1	Gonocoxites entirely fused medially, without suture; hypopygium with posteromedian filiform process	***Leptanilla* TH03 (THAILAND: Chiang Mai)**
–	Gonocoxites partly to fully separate medially; hypopygium without posteromedian filiform process	**2**
2	Ocelli absent (Fig. 45B); mandible articulated to gena (Fig. 46A)	***Leptanilla* zhg-bt03 (BHUTAN)**
–	Ocelli present (Fig. 45A); mandible fused to gena (Fig. 46B), rarely articulate (*Leptanilla* TH04)	**3**
3	Gonopodite shorter than (Fig. 47A), or subequal in length to, penial sclerites	**4**
–	Gonopodite distinctly longer than penial sclerites (Fig. 47B)	**6**
4	Internal margins of apical penial cleft distinctly separated; posteroventral gonocoxital margin entire (Fig. 48B)	***Leptanillaargamani* (Kugler, 1987), comb. nov. (ISRAEL, LEBANON)**
–	Internal margins of apical cleft of penial sclerites subparallel; posteroventral gonocoxital margin sinuate (Fig. 48A)	**5**
5	Color castaneous; posterior margin of compound eye linear in profile view	***Leptanillaindica* (Kugler, 1987), comb. nov. (INDIA: Kerala)**
–	Color yellowish to pallid; posterior margin of compound eye convex in profile view	***Leptanillaindica* (SRI LANKA)**
6	Dorsoventral margins of profemur not parallel (Fig. 49A)	**7**
–	Dorsoventral margins of profemur parallel (Fig. 49B)	**10**
7	Volsella bifid, ventral process bifurcated (Fig. 50A)	***Leptanilla* zhg-th02 (THAILAND: Phetchabun)**
–	Volsella usually bifid, rarely not (*Leptanilla* zhg-mm11), if bifid then ventral process entire (Fig. 50B)	**8**
8	Dorsal and ventral parossicular processes forming 90° angle; lengths of processes subequal	***Leptanilla* TH02 (THAILAND: Khon Kaen)**
–	Dorsal and ventral parossicular processes forming acute angle; ventral parossicular process 3× longer than length of dorsal process	**9**
9	Diameter of compound eye > 4× span of ocellar tubercle; gonopodital apices not recurved towards medial axis	***Leptanilla* zhg-th04 (THAILAND: Chaiyaphum)**
–	Diameter of compound eye only slightly greater than span of ocellar tubercle; gonopodital apices sharply recurved towards medial axis	***Leptanilla* zhg-th05 (THAILAND: Chaiyaphum)**
10	Gonostylar apex subtriangular, entire	**11**
–	Gonostylar apex tapering, entire or bifid (Fig. 47B)	**14**
11	Ventral margin of gonocoxites produced into two pairs of lobes (Fig. 51A); volsellae apparently not furcate (Fig. 52A)	***Leptanilla* zhg-mm11 (BURMA: Taninthayi)**
–	Ventral margin of gonocoxites not so produced (Fig. 51B); volsellae furcate (Fig. 52B)	**12**
12	Bifid processes of volsella oriented along lateromedial axis relative to genital capsule, lateral process shorter than medial process	***Leptanilla* MM01 (BURMA: Rakhine)**
–	Bifid processes of volsella oriented along dorsoventral axis relative to genital capsule, lengths of processes subequal	**13**
13	Larger species (WL > 0.5 mm); gonopodital suture absent	***Leptanilla* zhg-mm13 (BURMA: Taninthayi)**
–	Smaller species (WL ≤ 0.5 mm); gonopodital suture present, complete	***Leptanilla* cf. zhg-mm10 (BURMA: Taninthayi)**
14	Head not broader than long in full-face view, including compound eyes; gonostylar apex bifurcated (Fig. 47B)	***Leptanilla* TH08 (THAILAND: Surat Thani)**
–	Head broader than long in full-face view, including compound eyes; gonostylar apex entire	**15**
15	Penial sclerites distinctly longer than broad; volsella entire	**16**
–	Penial sclerites not distinctly longer than broad; volsella bifid	**17**
16	Gonocoxite with distodorsal carina; dorsal process of volsella recurved dorsally	***Leptanilla* TH04 (THAILAND: Chiang Mai)**
–	Gonocoxite without distodorsal carina; dorsal process of volsella recurved laterally	***Leptanilla* zhg-th05 (THAILAND: Chiang Mai)**
17	Gonostylar apex lobate in outline, covered with dense vestiture; coloration castaneous	***Leptanilla* TH06 (THAILAND: Chiang Mai)**
–	Gonostylar apex acuminate, glabrous; coloration beige	***Leptanilla* zhg-my16 (MALAYSIA: Selangor)**

### ﻿Male-based species-level key to the *Leptanillanajaphalla* species group

**Table:** 

1	Phallotreme at penial apex	**2**
–	Phallotreme proximad penial apex, anatomically ventral	**3**
2	Penial sclerites dorsoventrally compressed at apex, without dorsomedian lamina (Fig. 53A)	***Leptanilla* zhg-my03 (MALAYSIA: Sabah, Sarawak)**
–	Penial sclerites lateromedially compressed at apex, with dorsomedian lamina (Fig. 53B)	***Leptanilla* zhg-my04 (MALAYSIA: Sabah)**
3	Gonostylus present, articulated, tusk-like and lacking setae (Fig. 54); penial sclerites with recurved apical hook (Fig. 55A)	***Leptanilla* zhg-id01 (INDONESIA: Kalimantan Barat)**
–	Gonostylus absent; penial sclerites without recurved apical hook (Fig. 55B)	**4**
4	Apicolateral gonocoxital lamina subulate (Fig. 56A)	***Leptanillanajaphalla* sp. nov. (MALAYSIA: Sabah)**
–	Apicolateral gonocoxital lamina lanceolate (Fig. 56B)	***Leptanilla* zhg-my05 (MALAYSIA: Sabah)**

### ﻿Male-based species-level key to the *Leptanillahavilandi* species group

**Table:** 

1	ML > SL, with mandible flattened and paddle-like; lower metapleuron indistinct	***Leptanillaanomala* (Brues, 1925), comb. nov. (INDONESIA: Sumatra, Kalimantan Barat)**
–	ML ≤ SL, with mandible nub-like; lower metapleuron distinct	**2**
2	Mandalus not extending to mandibular apex; anteromedian ocellus orthogonally dorsal to compound eye in profile view (Fig. 57A)	***Leptanillacopiosa* (Petersen, 1968), comb. nov. (PHILIPPINES: Palawan)**
–	Mandalus extending to mandibular apex; anteromedian ocellus positioned posterodorsal to compound eye in profile view (Fig. 57B)	**3**
3	Gonostylus longer than gonocoxite (Fig. 58A)	***Leptanilla* zhg-my10 (MALAYSIA: Sabah)**
–	Gonostylus shorter than, or subequal in length to gonocoxite (Fig. 58B)	**4**
4	Penial apex produced into two ranks of aculeate processes; phallotremal rim glabrous	***Leptanilla* zhg-ph01 (PHILIPPINES: Camarines Sur; Quezon)**
–	Penial apex produced into robust ventral carina, without process dorsad to carina; phallotremal rim with vestiture	**5**
5	Penial apex entire	***Leptanilla* zhg-my14 (MALAYSIA: Sabah)**
–	Penial apex cleft	***Leptanilla* zhg-my11 (MALAYSIA: Sabah)**

### ﻿Male-based species-level key to the *Leptanillabethyloides* species group

**Table:** 

1	Mesoscutellum produced into recurved posterior process (Griebenow 2021: fig. 16B); LF2 > SL	***Leptanilla* zhg-th01 (THAILAND: Chiang Mai)**
–	Mesoscutellum not produced into recurved posterior process; LF2 ≤ SL	**2**
2	Penial sclerites lateromedially compressed, with dorsomedian carina	***Leptanilla* TH01 (THAILAND: Chiang Mai)**
–	Penial sclerites dorsoventrally compressed, without dorsomedian carina; gonopodital apex bifid	**3**
3	Smaller species; abdominal postsclerites V–VII anteroposteriorly compressed relative to those of III–IV	***Leptanilla* zhg-mm05 (BURMA: Taninthayi)**
–	Larger species; abdominal postsclerites V–VII with anteroposterior lengths subequal to those of III–IV	***Leptanillabethyloides* sp. nov. (CHINA: Hong Kong)**

### ﻿Male-based species-level key to the *Leptanillarevelierii* species group

**Table:** 

1	Gonostylus ellipsoid in outline (Griebenow 2020: fig. 11E); gonocoxites with complete ventromedian fusion	***Leptanillaastylina* (PHILIPPINES: Palawan)**
–	Gonostylus not ellipsoid; gonocoxites without ventromedian fusion	**2**
2	Protibial length 0.5× profemoral length	**3**
–	Protibial length > 0.5× profemoral length	**4**
3	Length of probasitarsal seta less than that of calcar	***Leptanillaafricana* Baroni Urbani, 1977 (NIGERIA)**
–	Length of probasitarsal seta subequal to that of calcar	***Leptanilla* TH09 (THAILAND: Phetchabun)**
4	Gonostylus bifurcated or emarginate	**5**
–	Gonostylus entire, apex tapering or truncate	**14**
5	Abdominal segment II broadly joined to abdominal segment III (Santschi 1907: fig. 3)	***Leptanillaminuscula* Santschi, 1907 (TUNISIA)**
–	Abdominal segment III narrowly joined to abdominal segment III	**6**
6	Ventromedial gonocoxital margin with sinuate process	***Leptanillatanit* Santschi, 1907 (TUNISIA)**
−	Ventromedial gonocoxital margin entire	**7**
7	Gonostylar apex with obtuse tooth subtending dorsal process	***Leptanilla* GR02 (GREECE: Rhodes)**
–	Gonostylar apex lacking obtuse tooth subtending dorsal process	**8**
8	Ventromedian margin of gonostylus excavated proximad apical furca	***Leptanilla* zhg-au02 (AUSTRALIA: New South Wales)**
–	Ventromedian margin of gonostylus entire proximad apical furca	**9**
9	Dorsal process of gonostylar apex acuminate	**10**
–	Dorsal process of gonostylar apex rounded	**11**
10	Processes of gonostylar apex large, with apex appearing deeply bifurcated	***Leptanillatenuis* Santschi, 1907 (TUNISIA)**
–	Processes of gonostylar apex small, with apex appearing nearly truncate	***Leptanilla* zhg-mm02 (BURMA: Taninthayi)**
11	Penial apex entire	**12**
–	Penial apex emarginate	**13**
12	PTL ≈ PTH	***Leptanilla* GR01 (GREECE: Rhodes)**
–	PTL > PTH	***Leptanilla* zhg-id02 (INDONESIA: Sulawesi Tenggara)**
13	Internal margins of apical penial cleft distinctly separated, ventral gonostylar process narrower than dorsal process	***Leptanillabifurcata* Kugler, 1987 (ISRAEL)**
−	Internal margins of apical penial cleft adjacent, gonostylar processes subequal in breadth	***Leptanillaisraelis* Kugler, 1987 (ISRAEL)**
14	Gonostylar apex not tapering	**15**
–	Gonostylar apex tapering	**17**
15	Gonostylus with expanded, rounded apex (Fig. 59A)	***Leptanillaislamica* Baroni Urbani, 1977 (YEMEN; OMAN)**
–	Gonostylus with apex not expanded (Fig. 59B)	**16**
16	Outline of penial sclerites attenuate in posterodorsal view (Fig. 60A)	***Leptanillaalexandri* Dlussky, 1969 (UZBEKISTAN)**
–	Outline of penial sclerites elliptical in posterodorsal view (Fig. 60B)	***Leptanillajaponica* Baroni Urbani, 1977 (JAPAN: Honshu; CHINA: Hong Kong)**
17	Gonostylar apex acuminate	**18**
–	Gonostylar apex digitate	**25**
18	Oblique mesopleural sulcus traversing posterior > 0.5× of mesopleuron	**19**
–	Oblique mesopleural sulcus traversing posterior ≤ 0.5× of mesopleuron	**20**
19	Penial sclerites broad in posterodorsal view, apex entire; Rsf1+Mf1 present	***Leptanillajavana* (Wheeler & Wheeler, 1930) (INDONESIA: Java)**
–	Penial sclerites narrow in posterodorsal view, apex emarginate; Rsf1+Mf1 absent	***Leptanilla* zhg-ke01 (KENYA: Laikipia)**
20	Abdominal sternite II without distinct subpetiolar process (Fig. 61A)	***Leptanilla* zhg-bt02 (BHUTAN)**
–	Abdominal sternite II with distinct subpetiolar process (Fig. 61B)	**21**
21	*2s-rs*+R+4-6 absent from forewing (Fig. 62A)	**22**
–	*2s-rs*+R+4-6 present in forewing (Fig. 62B)	**24**
22	Posterior face of petiolar node shallower than anterior face; genital capsule subequal in overall dimensions to abdominal segment II	***Leptanilla* zhg-bt01 (BHUTAN)**
–	Posterior face of petiolar node not shallower than anterior face; dimensions of genital capsule conspicuously greater than those of abdominal segment II	***23***
23	Oblique mesopleural sulcus adjoining metapectal-propodeal complex	>**Leptanilla zhg-au03 (AUSTRALIA: Queensland)**
–	Oblique mesopleural sulcus not adjoining metapectal-propodeal complex	***Leptanilla* zhg-ke02 (KENYA: Kakamega)**
24	Apicolateral margins of penial sclerites emarginate; smaller species (WL = 0.37–0.44 mm) (*n* = 6)	***Leptanillacharonea*Barandica et al., 1994 (SPAIN)**
–	Apicolateral margins of penial sclerites entire; larger species (WL = 0.46–0.50 mm) (*n* = 3)	**Leptanillacf.zaballosiLópez et al., 1994 (SPAIN)**
25	Penial sclerites broader than long (Fig. 63A)	***Leptanilla* GR03 (GREECE: Rhodes; TURKEY: Muğla)** ***Leptanilla* zhg-tr01 (TURKEY: Muğla)**
–	Penial sclerites longer than broad (Fig. 63B)	**26**
26	Gonostylus not articulated to gonocoxite	***Leptanillaexigua* Santschi, 1908 (TUNISIA)**
–	Gonostylus articulated to gonocoxite	**27**
27	Abdominal sternite II produced ventrally, forming curve in profile view	**28**
–	Abdominal sternite II not produced ventrally, linear in profile view	**29**
28	Gonocoxites with apicoventral laminae	***Leptanilla* zhg-au05 (AUSTRALIA: Queensland)**
–	Gonocoxites without apicoventral laminae	***Leptanilla* zhg-au01 (AUSTRALIA: Queensland)**
29	Oblique mesopleural sulcus present; Sc+R+Rs tubular	***Leptanilla* zhg-au07 (AUSTRALIA: Queensland)**
–	Oblique mesopleural sulcus absent; Sc+R+Rs absent	***Leptanillaaustralis* Baroni Urbani, 1977 (SOUTH AFRICA: Cape Province)**

**Figure 34.**
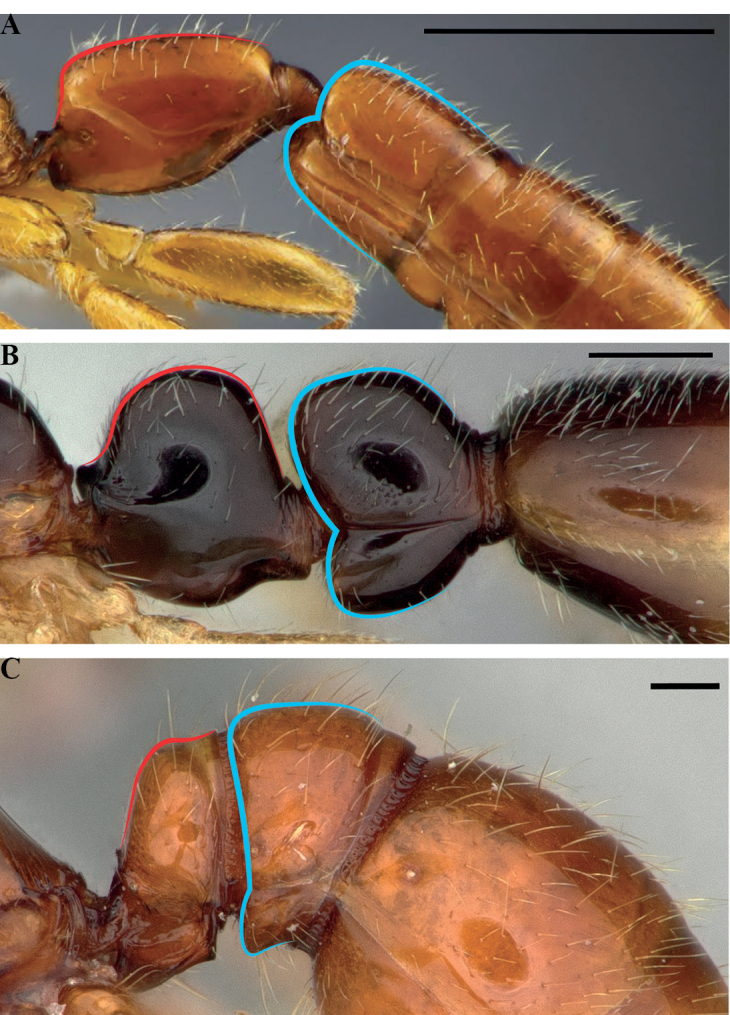
Abdominal segments II–III of female Leptanillinae, profile view. Abdominal tergite II outlined in red; anterior of abdominal segment III outlined in blue **A***Opamyrmahungvuong* (AKY05vii17-06) (Yamada et al. 2020: fig. 1C), worker **B***Protanillagengma* (CASENT0179564), worker **C***Protanilla* id01 (MCZENT00728282), gyne. Scale bars: 0.5 mm (**A**); 0.2 mm (**B, C**).

**Figure 35.**
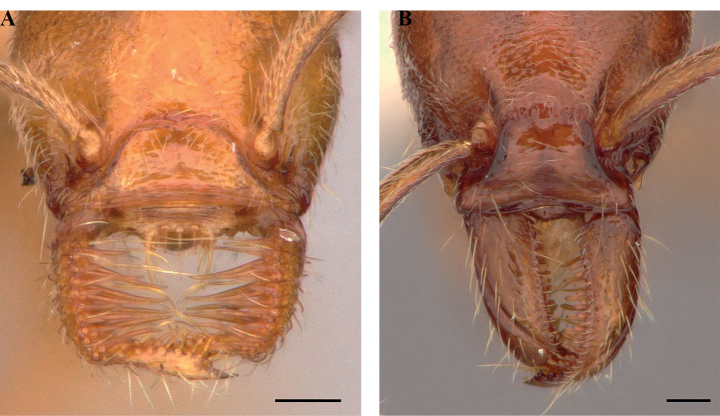
Anterior of the worker head in *Protanilla*, full-face view **A***Protanillaizanagi* (CASENT0842850) **B***Protanillajongi* (CASENT0842693). Scale bars: 0.1 mm.

**Figure 36.**
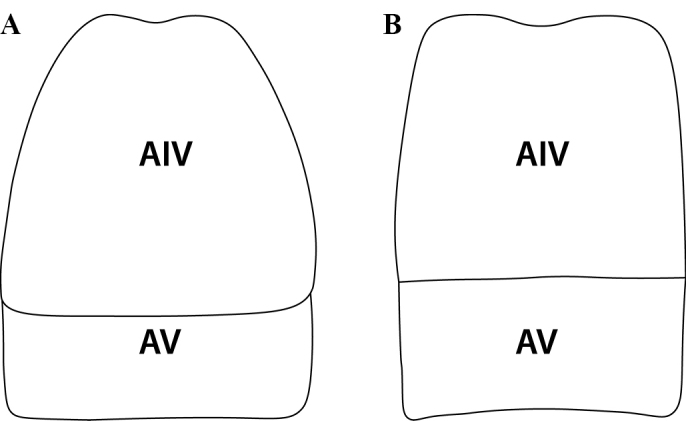
Worker abdominal segments IV-V in *Leptanillataiwanensis* (**A**) and *Leptanillaoceanica* (**B**), diagrammatic dorsal view. Fig. 36B redrawn from Baroni Urbani (1977: fig. 19).

**Figure 37.**
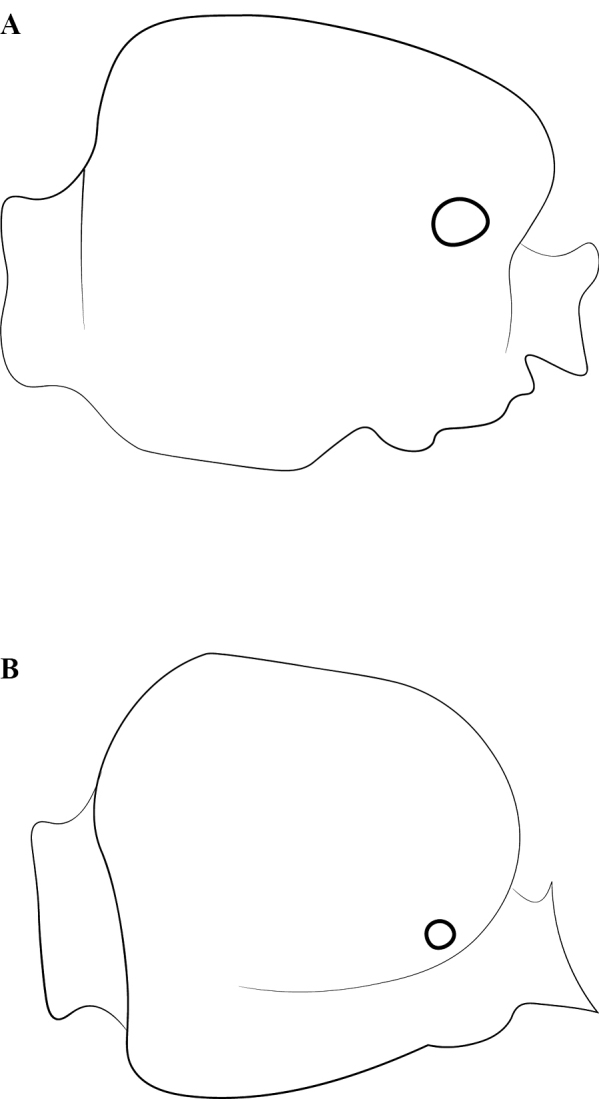
Profile condition of the petiole in the *Leptanillarevelierii* species group **A***Leptanillapoggii* (after Mei 1995: fig. 4) **B***Leptanillatheryi* (after Mei 1995: fig. 6).

**Figure 38.**
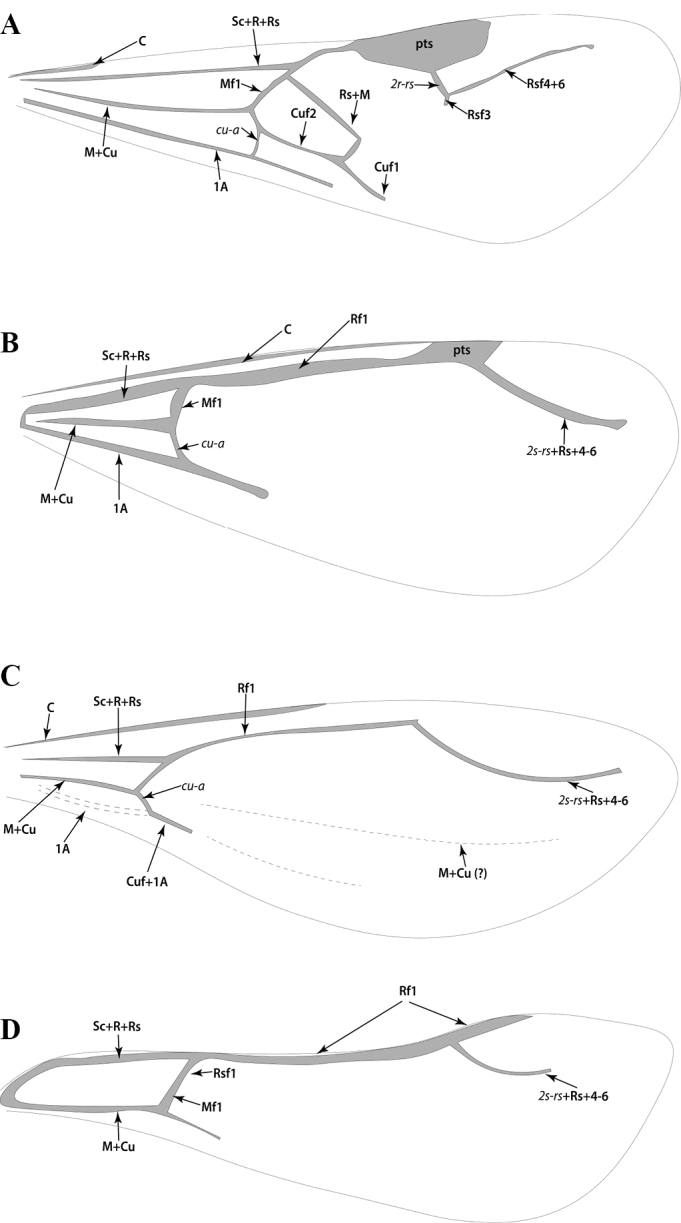
Exemplars of male wing venation across the Leptanillinae, diagrammatic **B, C** are typological generalizations of male wing venation in the clades that they represent **A***Opamyrmahungvuong***B***Protanilla***C***Leptanillanajaphalla* species group **D***Leptanillajavana*. Abbreviation: pts = pterostigma.

**Figure 39.**
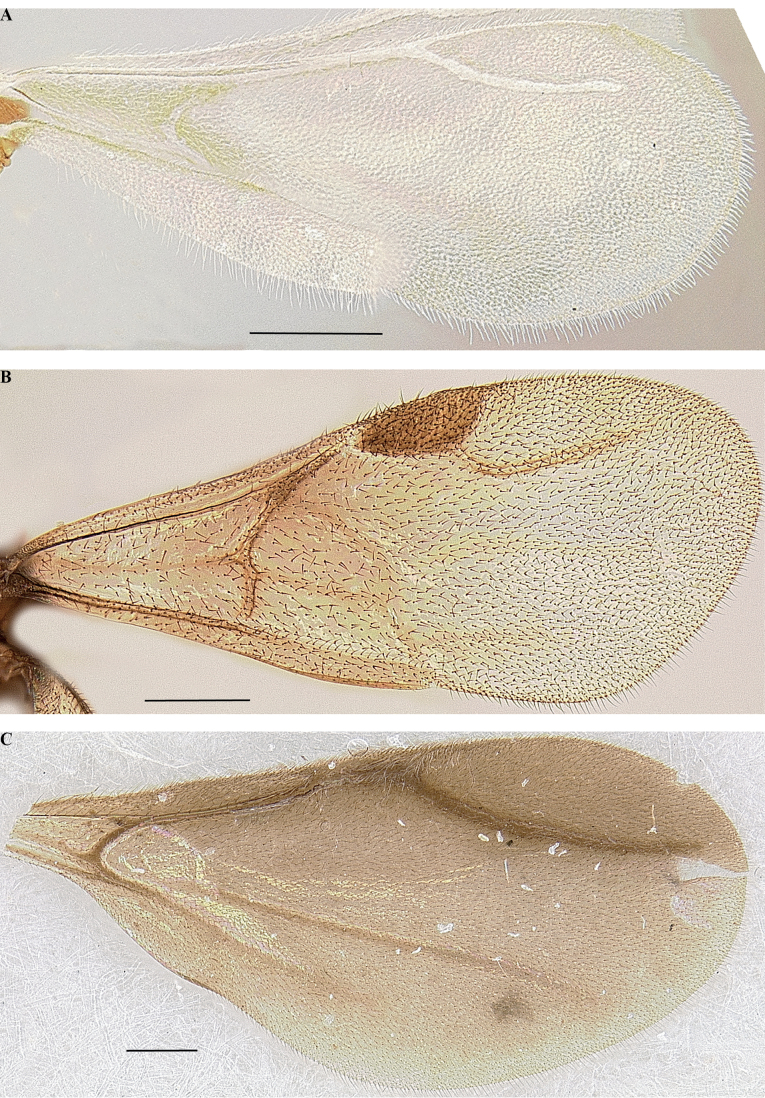
Condition of the pterostigma across the Leptanillini, mal **A***Leptanillaindica* (CASENT0106380) **B***Protanilla* zhg-vn01 (CASENT0842613) **C***Leptanilla* zhg-my05 (CASENT0842571). Scale bars: 0.25 mm (**A, B**); 0.2 mm (**C**).

**Figure 40.**
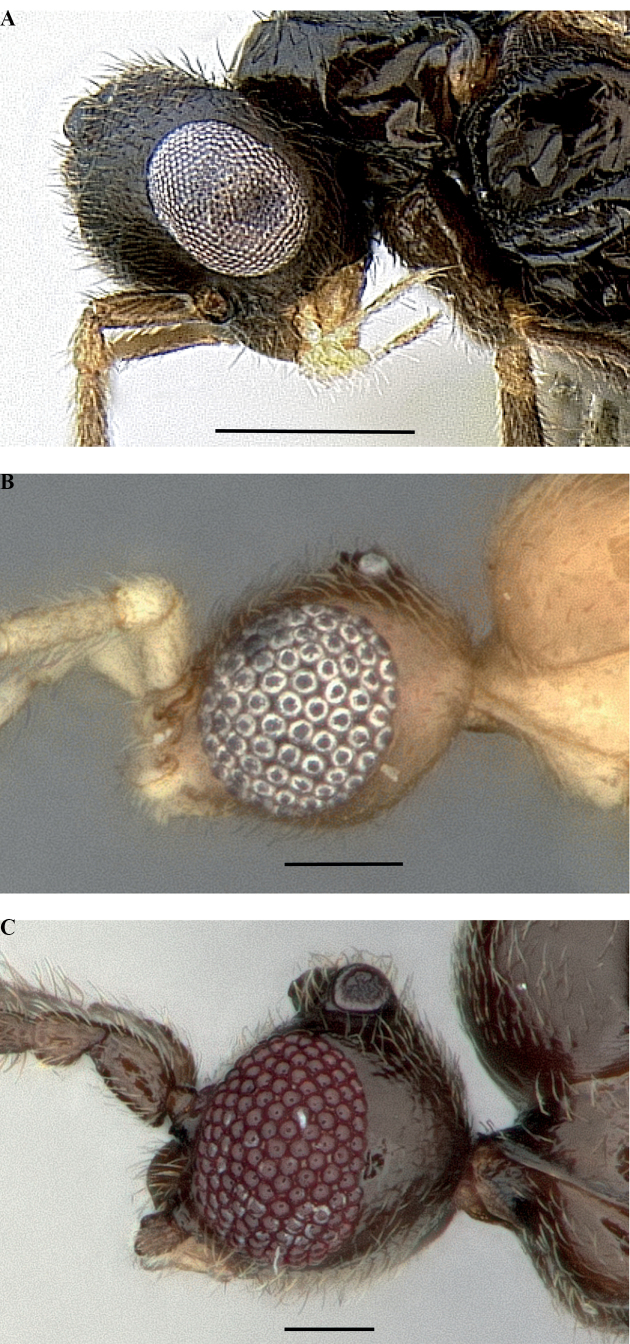
Condition of the male ocelli in the Leptanillini, profile view **A***Protanillalini* (OKENT0011097) **B***Leptanillaindica* (CASENT0106366) **C***Leptanillaargamani* (CASENT0235253). Scale bars: 0.25 mm (**A**); 0.1 mm (**B, C**).

**Figure 41.**
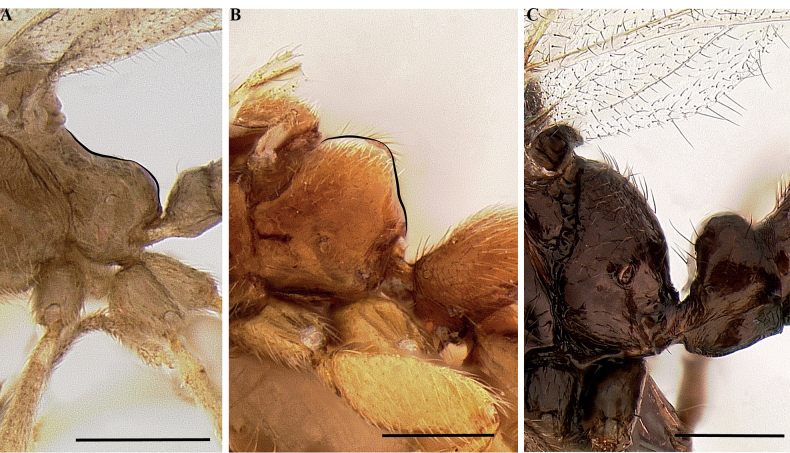
Propodeal outline in profile view across male Leptanillini, after Griebenow (2021: fig. 17). Propodeum outlined in black in Fig. 16A–C**A***Leptanilla* zhg-bt03 (CASENT0106384) **B***Leptanilla* zhg-my02 (CASENT0106456) **C***Protanillalini* (OKENT0011097). Scale bars: 0.15 mm (**A, C**); 0.2 mm (**B**).

**Figure 42.**
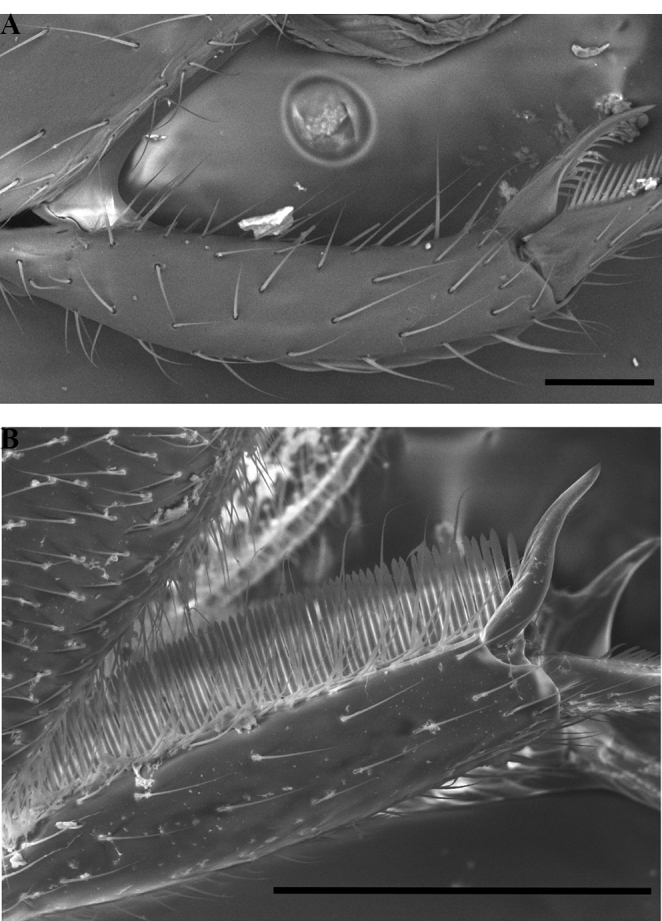
Protibia in male *Leptanilla*, posterior view **A***Leptanilla* zhg-my11 (CASENT0842593) **B***Leptanilla* zhg-my04 (CASENT0842555). Scale bars: 0.05 mm (**A**); 0.2 mm (**B**).

**Figure 43.**
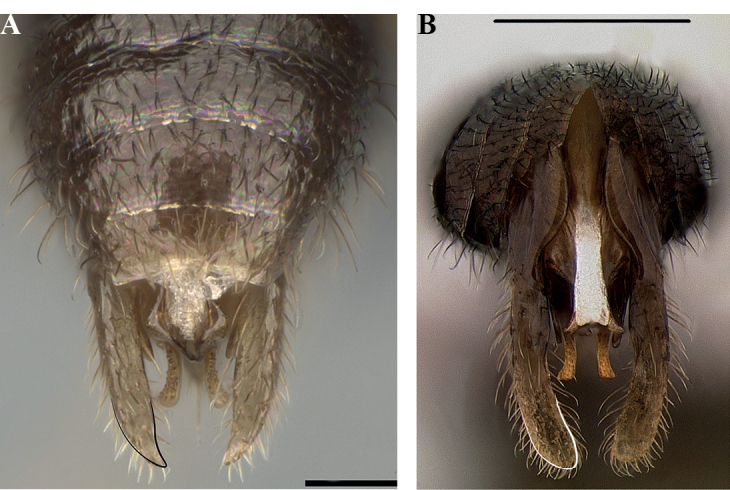
Gonostyli in *Protanilla*, posterodorsal view. After Griebenow (2020: fig. 9C) **A***Protanilla* TH01 (CASENT0119776; Michele Esposito) **B***Protanillalini* (OKENT0011097). Scale bars: 0.1 mm (**A**); 0.5 mm (**B**).

**Figure 44.**
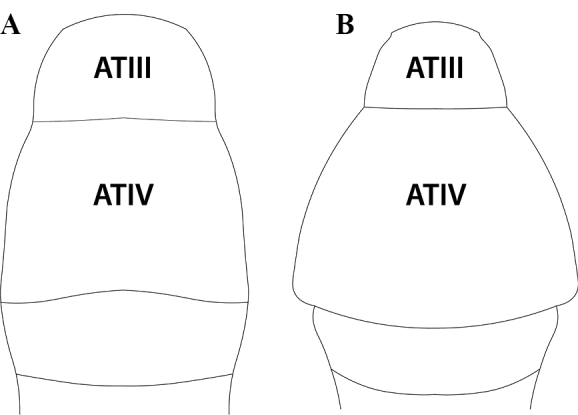
Proportions of male abdominal tergites III-IV in *Protanilla* zhg-vn01 (**A**) versus *Protanillalini* (**B**), diagrammatic. Abbreviation: AT = abdominal tergite.

**Figure 45.**
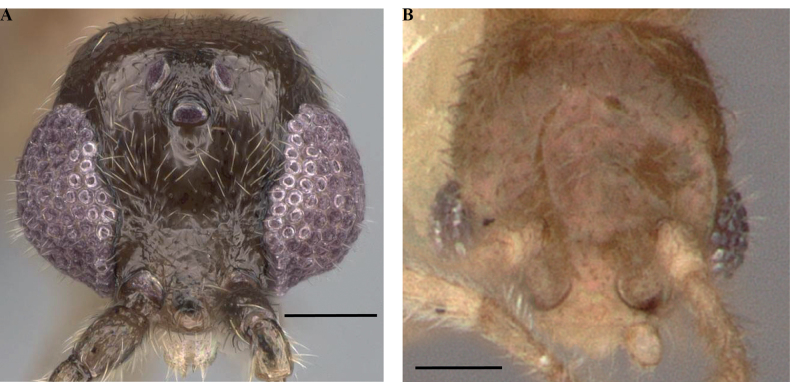
Condition of male ocelli in the Leptanillini, full-face view **A***Leptanilla* TH02 (CASENT0119531; Shannon Hartman) **B***Leptanilla* zhg-bt03 (CASENT0106384). Scale bars: 0.1 mm.

**Figure 46.**
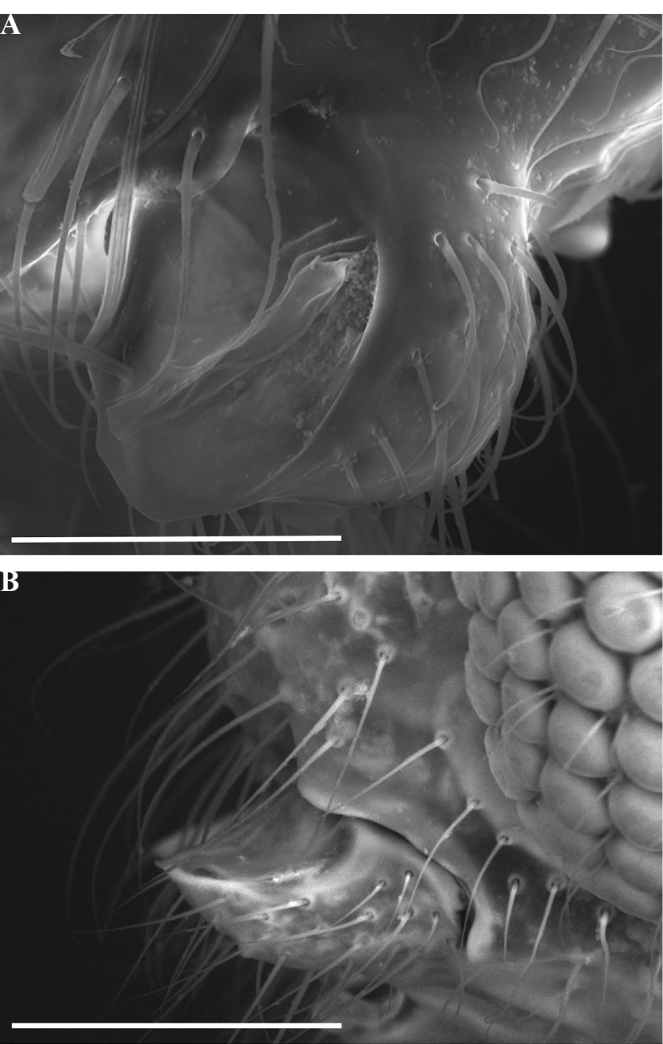
Articulation of the male mandible in the *Leptanillathai* species group **A***LeptanillaIndica* (CASENT0106377) **B***Leptanilla* zhg-bt03 (CASENT0106384). Scale bars: 0.03 mm (**A**); 0.04 mm (**B**).

**Figure 47.**
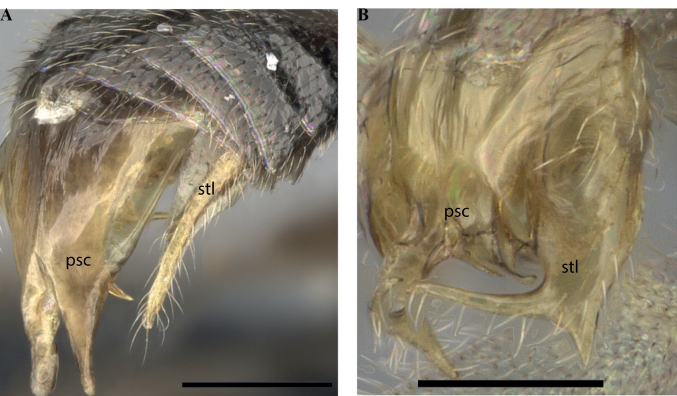
Proportions of the penial sclerites to the gonopodites in the *Leptanillathai* species group **A***Leptanillaargamani***B***Leptanilla* TH08. Abbreviations: stl = gonostyli; psc = penial sclerites. Scale bars: 0.2 mm (**A**); 0.1 mm (**B**).

**Figure 48.**
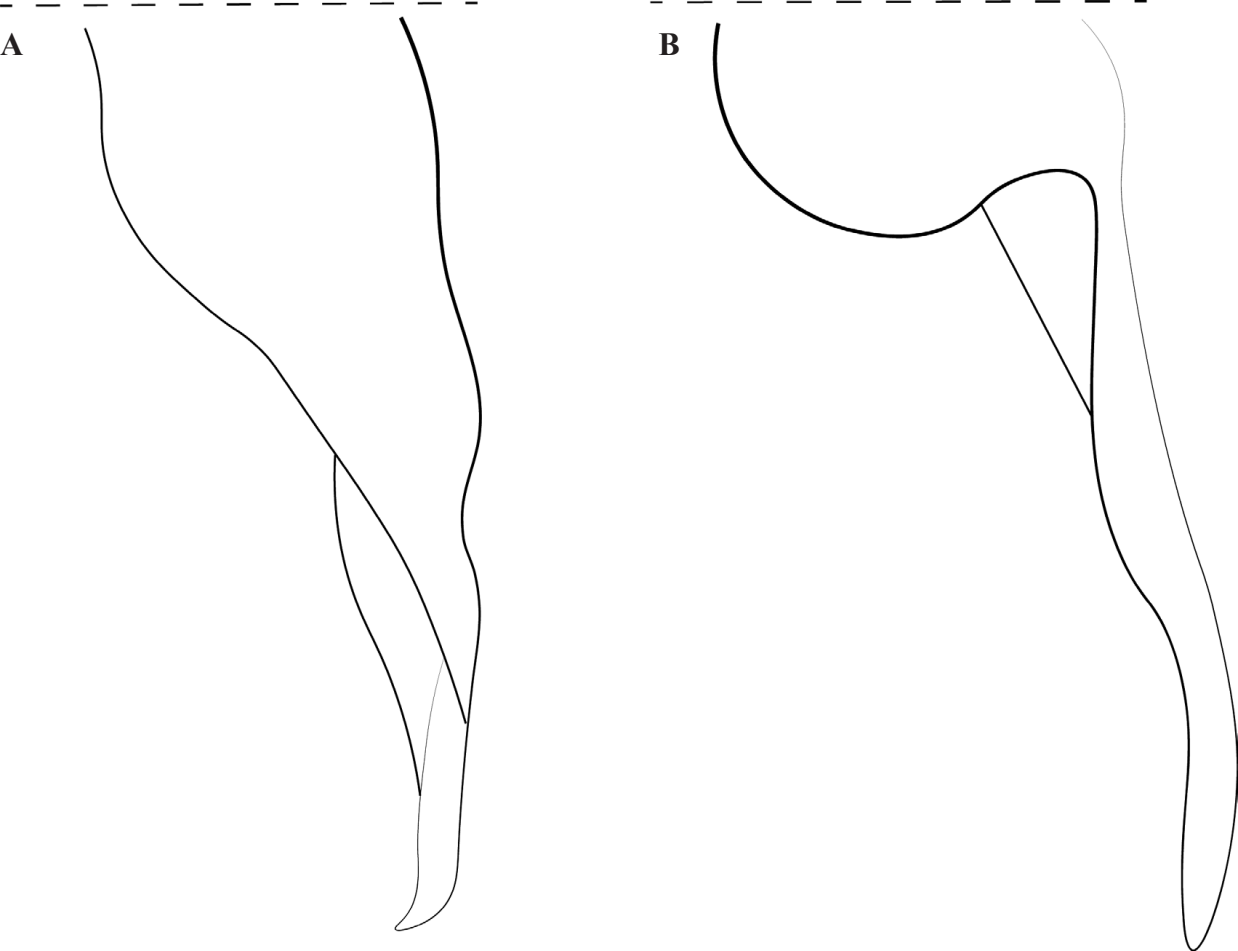
Outline of the gonopodites in *Leptanillaindica* (**A**) and *Leptanillaargamani* (**B**), ventral view, diagrammatic. Redrawn from Kugler (1987: figs 18, 22).

**Figure 49.**
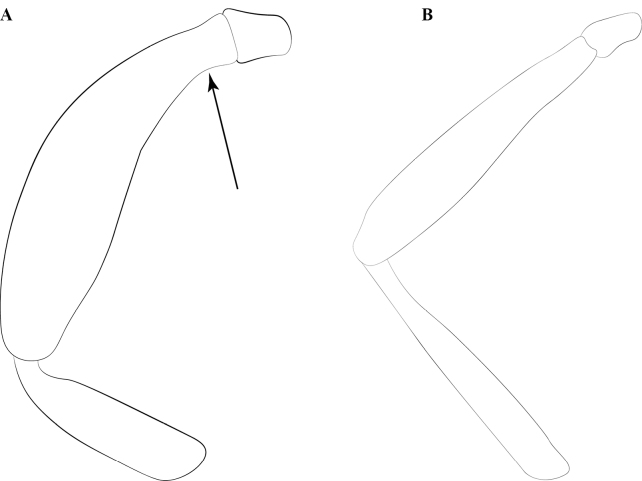
Male protrochanter, profemur, and protibia in the *Leptanillathai* species group, diagrammatic. After Griebenow (2020: fig. 11B) **A***Leptanilla* zhg-th02 **B***Leptanilla* TH04.

**Figure 50.**
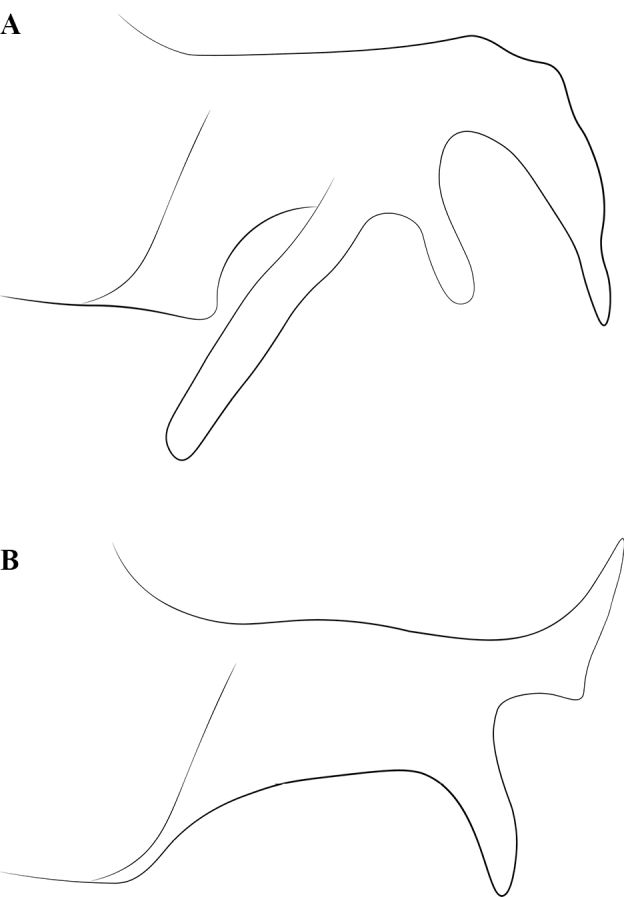
Volsella of the *Leptanillathai* species group, medial view, diagrammatic, after Griebenow (2020: fig. 11C). Not to scale **A***Leptanilla* zhg-th02 **B***Leptanilla* TH02.

**Figure 51.**
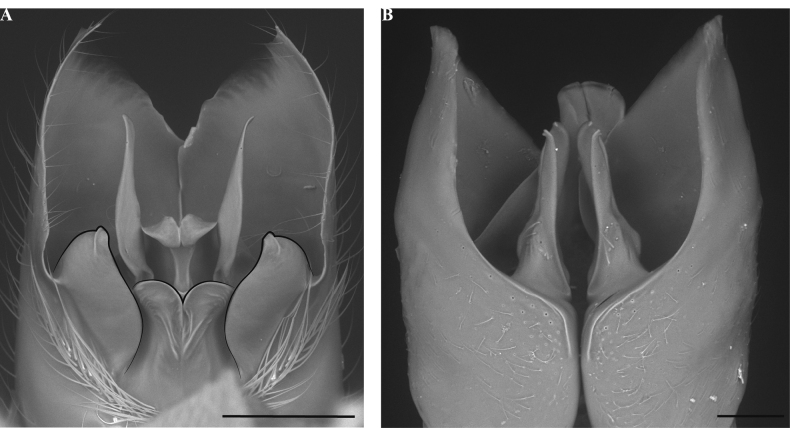
Gonopodital margins in the *Leptanillathai* species group, ventral view. Gonocoxital lobes outlined in black **A***Leptanilla* zhg-mm11 (CASENT0842848) **B***Leptanilla* zhg-mm13 (CASENT0842670). Scale bars: 0.15 mm (**A**); 0.06 mm (**B**).

**Figure 52.**
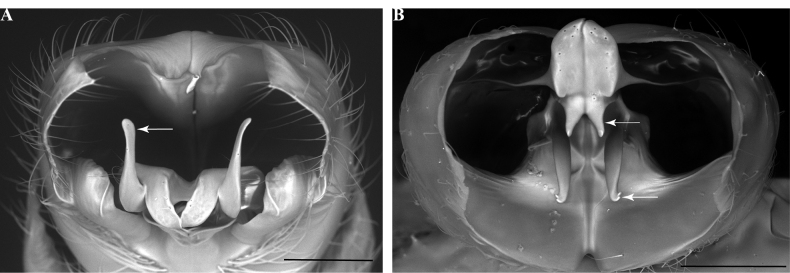
Volsellae in the *Leptanillathai* species group, posterior view. Volsellar processes marked with arrows **A***Leptanilla* zhg-mm11 (CASENT0842848) **B***Leptanilla* zhg-mm13 (CASENT0842670). Scale bar: 0.1 mm.

**Figure 53.**
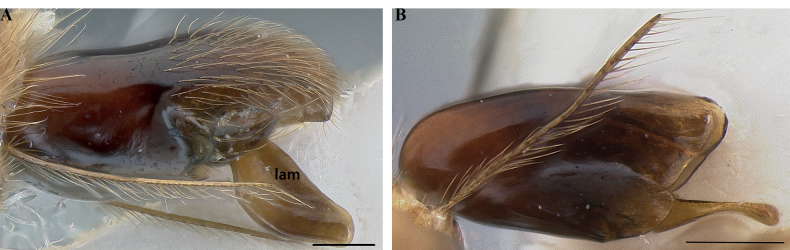
Male genitalia in the *Leptanillanajaphalla* species group, profile view. Abbreviation: lam = dorsomedian lamella of penial sclerites **A***Leptanilla* zhg-my04 (CASENT0842558) **B***Leptanilla* zhg-my03 (CASENT0842545). Scale bar: 0.2 mm.

**Figure 54.**
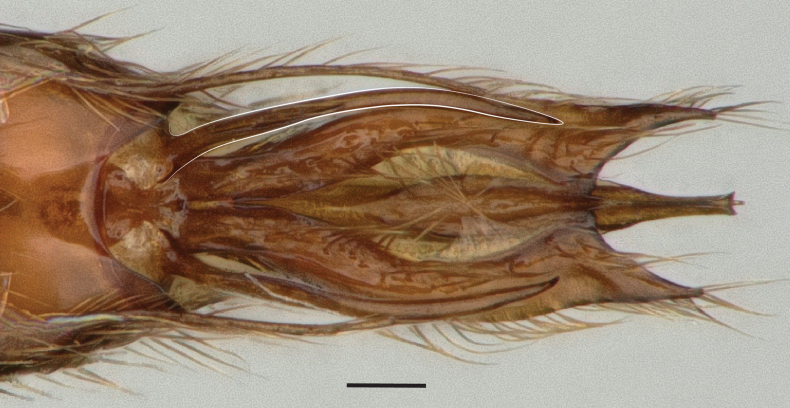
Male genitalia of *Leptanilla* zhg-id01 (CASENT0842625), ventral view. Gonostylus outlined in white. Scale bar: 0.1 mm.

**Figure 55.**
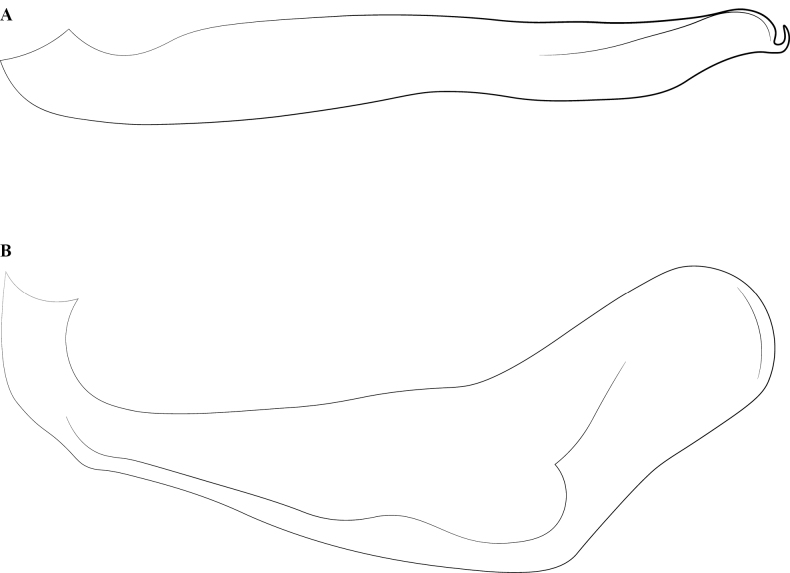
Penial sclerites of the *Leptanillanajaphalla* species group, profile view, diagrammatic. Base (left) partly concealed by gonocoxites in situ **A***Leptanilla* zhg-id01 **B***Leptanilla* zhg-my05.

**Figure 56.**
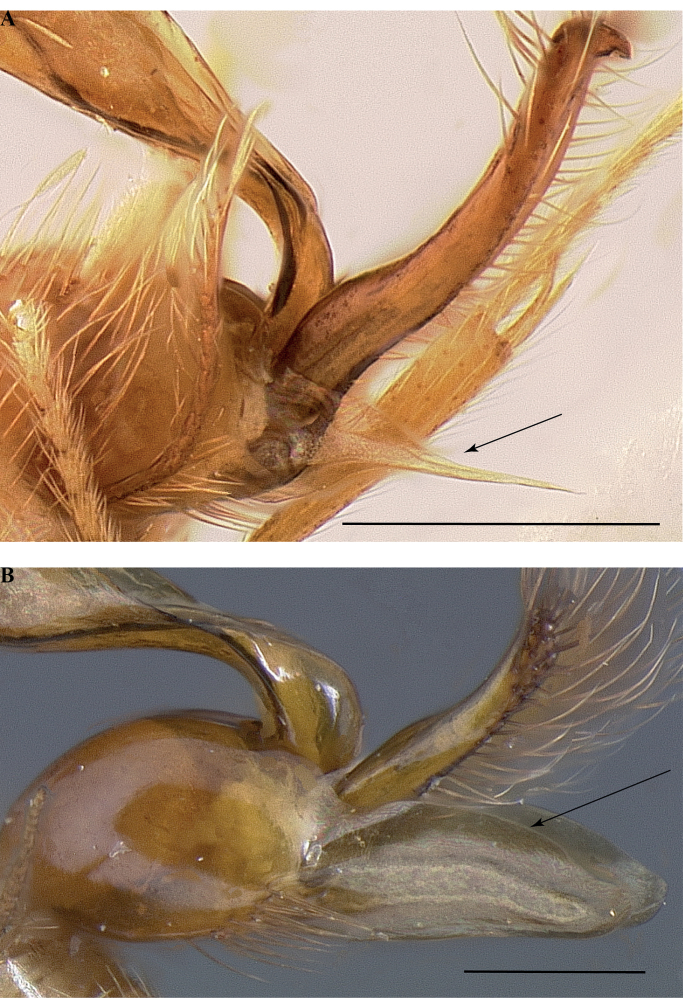
Apicolateral gonocoxital laminae in the *Leptanillanajaphalla* species group, profile view **A***Leptanilla* zhg-my02 (CASENT0106427) **B***Leptanilla* zhg-my05 (CASENT0842571). Scale bars: 0.3 mm (**A**); 0.5 mm (**B**).

**Figure 57.**
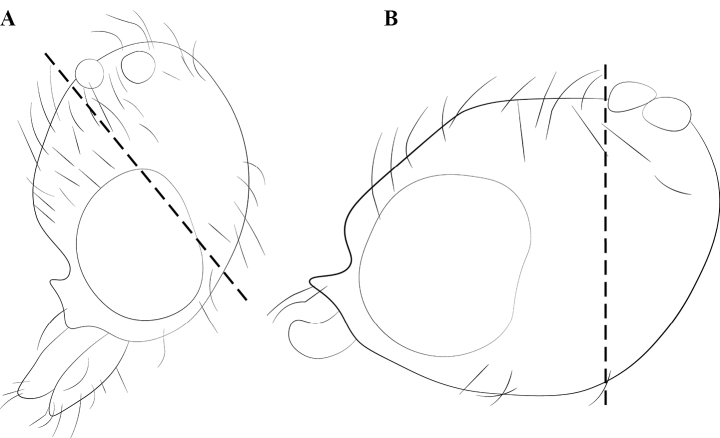
Position of the male anteromedian ocellus relative to the compound eye in *Leptanilla*, diagrammatic, after Griebenow (2020: fig. 12B) **A***Leptanillacopiosa***B***Leptanilla* zhg-my10.

**Figure 58.**
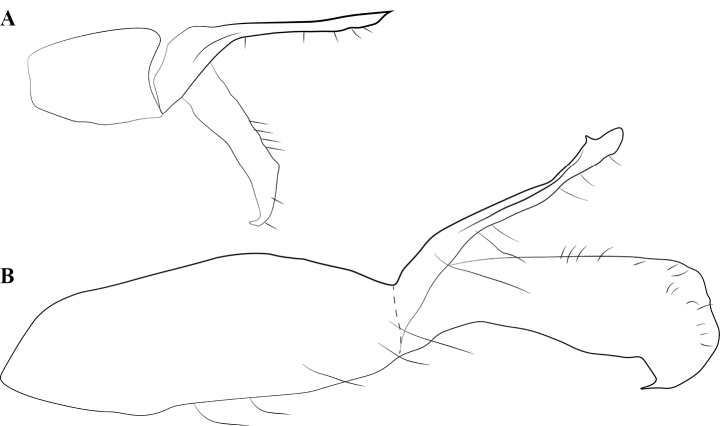
Male genitalia in the *Leptanillahavilandi* species group, profile view, diagrammatic, after Griebenow (2020: fig. 13A). Figures to scale **A***Leptanilla* zhg-my10 **B***Leptanilla* zhg-my11.

**Figure 59.**
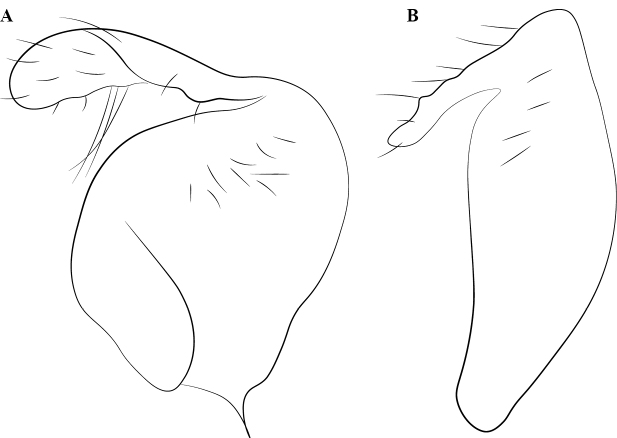
Gonostylar shape in the *Leptanillarevelierii* species group, after Griebenow (2020: fig. 13F) **A***Leptanillaislamica***B***Leptanillaaustralis*.

**Figure 60.**
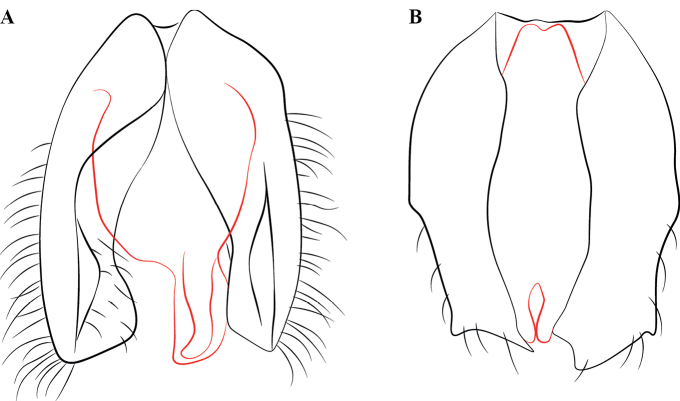
Dorsal outline of the penial sclerites (red) in the *Leptanillarevelierii* species group, diagrammatic, after Griebenow (2020: fig. 13G) **A***Leptanillaalexandri***B***Leptanillajaponica*.

**Figure 61.**
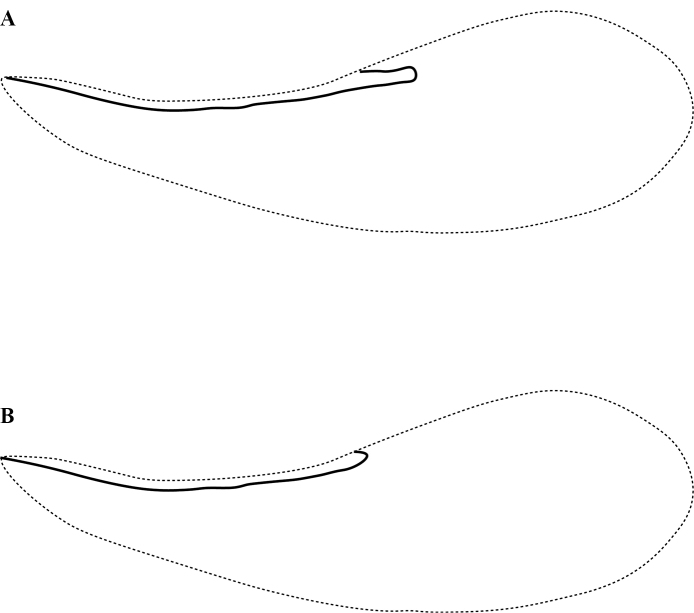
Presence (**A**) versus absence (**B**) of forewing *2s-rs*+R+4-6 in males of the *Leptanillarevelierii* species group, diagrammatic.

**Figure 62.**
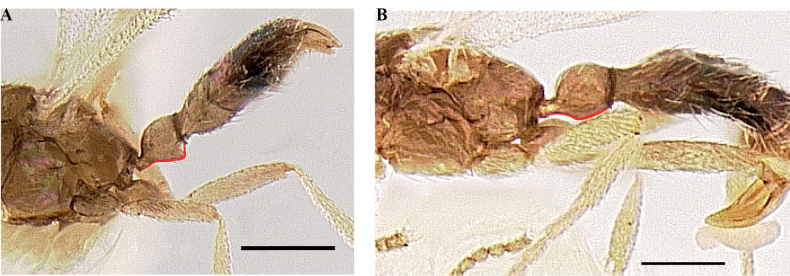
Abdominal segment II in males of the *Leptanillarevelierii* species group, profile view. Abdominal sternite II outlined in red **A***Leptanilla* zhg-bt01 (CASENT0842617) **B***Leptanilla* zhg-bt02 (CASENT0842612). Scale bars: 0.125 mm (**A**); 0.100 mm (**B**).

**Figure 63.**
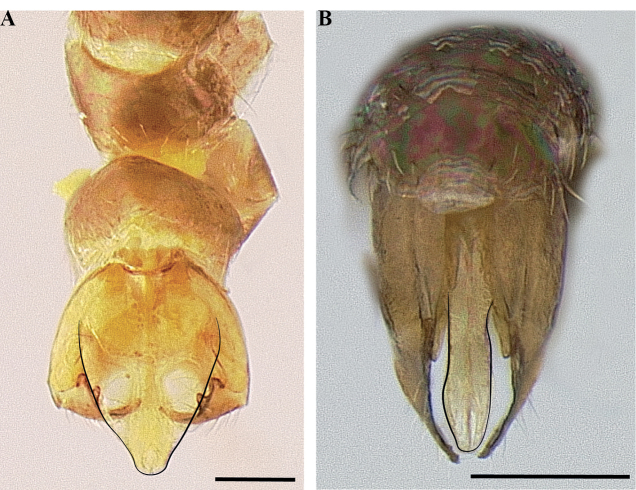
Penial sclerites in the *Leptanillarevelierii* species group, outlined in black, posterodorsal view **A***Leptanilla* GR02 (CASENT0106068) **B***Leptanilla* zhg-au01 (CASENT0758873). Scale bar: 0.1 mm.

## ﻿Discussion

### ﻿Taxonomic history

Writing of the subfamily Leptanillinae, Brown (1954: 28) opined that “ … it is doubtful that we shall ever be certain of its true affinities.” Concomitantly, the classification of the Leptanillinae relative to other Formicidae has a convoluted history. Extreme morphological derivation (in males, larvae, and both female castes), varying markedly across the few lineages of the clade, is responsible for this.

For most of its taxonomic history, the subfamily Leptanillinae was subsumed within (Emery 1910), or affiliated with, the army ants (Dorylinae*sensu* Ashmead) (Baroni Urbani 1989; Hölldobler and Wilson 1990), with *Leptanilla* having been described within the Dorylinae (Emery 1870). Despite ill-interrogated placement in the Myrmicinae by many early authors (Emery and Forel 1879; Dalla Torre 1893; Ashmead 1905; Emery 1910), the description of dichthadiiform gynes in *Leptanilla* was interpreted as supporting its placement within the Dorylinae (Emery 1904), while Santschi (1907) asserted the similarity of putative male *Leptanilla* to male army ants. Wheeler (1923) was the first to elevate the then-monobasic Leptanillini to subfamily rank, an action also argued for by Wheeler (1928) and Wheeler and Wheeler (1965) due to the dissimilarity of the larval habitus between the Dorylinae and Leptanillinae. *Leptanilloides* (Dorylinae) was placed as Formicidae incertae sedis and likened to the Leptanillinae by Borgmeier (1955) due to that genus exhibiting “a mixture of characters of the Ecitonini (i.e., New World army ants) and Leptanillinae” (Borgmeier 1955: 652), but Brown (1975: 34) classified *Leptanilloides* within the “doryline section” (Bolton 1990a) due to its close resemblance to *Sphinctomyrmexsensu lato*, a classification followed by all subsequent authors and confirmed by phylogenetic inference from molecular data (e.g., Brady et al. 2014).

With the description of the tribe Anomalomyrmini within the Leptanillinae, Bolton (1990b: 267) “dispute(d) the indisputability” of leptanilline kinship with army ants, since *Protanilla* gynes are not dichthadiiform (Baroni Urbani and de Andrade 2006; Billen et al. 2013; Hsu et al. 2017), and dichthadiigynes are unequivocally homoplasious in their other occurrences across the Formicidae (Bolton 1990a). Bolton (1990b) transferred *Apomyrma* to the Leptanillinae from the Ponerinae*sensu*Bolton (1990b) and proposed that the resemblance of doryline to leptanilline gynes was homoplasious. Based on the theorized kinship of *Apomyrma* to the Leptanillinae (Apomyrminae and Leptanillinae constituting the “leptanillomorph subfamilies” *sensu*Bolton (2003)), these lineages were hypothesized to have affinity with the Amblyoponinae, or more generally the “poneroid” clade (Ward 2007).

The advent of molecular sequencing supported none of the above hypotheses: instead, Leptanillinae was consistently supported as an early-diverging lineage of the Formicidae not akin to *Apomyrma*, which was recovered as a poneroid, sister to the Amblyoponinae. In addition, Ward and Fisher (2016) robustly recovered the monotypic genus *Opamyrma*, which had been described within the Amblyoponinae on account of character states closely resembling those of *Apomyrma* (e.g., abdominal sternite II reduced), as sister to the remaining Leptanillinae (Ward and Fisher 2016). This inference is corroborated by male morphology.

The Leptanillinae have been afflicted by a dual taxonomy since the description of the first putative males by Santschi (1907, 1908). The first males of *Leptanilla* were described without association with workers, justified by purported similarity in head morphology, and “only with some doubt (n’est qu’avec doute)” (Santschi 1907: 312). The genus *Phaulomyrma* was erected for *Leptanillajavana* (Wheeler & Wheeler, 1930) and *Leptanillatanit* Santschi, 1907, both known only from males (Wheeler and Wheeler 1930), whereas the bizarre monotypic genus *Scyphodon*, described by Brues (1925) as Hymenoptera incertae sedis, was found to represent a male leptanilline (Petersen 1968; Boudinot 2015), although Ogata et al. (1995) argued against the placement of *Scyphodon* in the Formicidae. The genera *Noonilla* and *Yavnella* were also described in the Leptanillinae based solely upon male specimens (Petersen 1968; Kugler 1987). Ogata et al. (1995) was the first to associate male and worker leptanilline specimens, describing the male of *Leptanillajaponica*, which was previously known from workers (Baroni Urbani 1977), and confirming the hypothesis of Santschi (1907). The two genera for which the tribe Anomalomyrmini was established were each initially known only from workers (*Protanilla*) or gynes (*Anomalomyrma*) (Bolton 1990b). Consideration of morphology illuminated by phylogenetic inference (Borowiec et al. 2019; Griebenow 2020, 2021; Griebenow et al. 2022) demonstrates a lack of reciprocal monophyly, and the two are here synonymized. Males were only subsequently associated with *Protanilla* (namely the *Protanillarafflesi* species group) by means of phylogenomic inference (Griebenow 2020). The Opamyrmini have avoided comparable taxonomic problems, with the collection of the male of *O.hungvuong* in association with females (Yamada et al. 2020).

### ﻿Biogeography and ecology

The Leptanillinae are, as per the 95% credibility interval inferred for the crown age of this clade by Borowiec et al. (2019), no older than the beginning of the Cenozoic Era (66 mya). The crown age of the Leptanillinae is no older than the estimated origins of several ant clades that have a circumtropical or cosmopolitan distribution, including *Odontomachus* (Ponerinae: Ponerini) (Schmidt 2013) and *Camponotus* (Formicinae: Camponotini) (Blaimer et al. 2015). Yet, curiously, the Leptanillinae are restricted to the Old World. The bulk of leptanilline diversity resides in the humid tropics, with the few temperate lineages (e.g., *Leptanillataiwanensis*; Man et al. 2017) being close kin of tropical ones. This implies that the origin of the Leptanillinae occurred in tropical climates, conforming to the overall tendency observed in the Formicidae (Economo et al. 2018). In the absence of other data to explain the absence of this clade from the New World, I predict that leptanilline ants originated after the closure of the Thulean and Beringian land bridges to tropical biota, but this prediction remains to be tested.

The notable absence of the Leptanillinae from the Neotropics elicits inquiry into which ants occupy a similar ecological niche in this ecoregion. In terms of functional morphology and behavior, *Leptanilloides* differs from leptanilline ants in the presence of cincti on abdominal segments IV–VII and in being an obligate predator of ant brood, rather than hunting geophilomorph centipedes; despite their name, these minute dorylines are not a Neotropical analog to the Leptanillinae. Rather, it is probable that centipede predators such as *Prionopelta* and *Fulakora* (Amblyoponinae), which often display LHF (Ito and Billen 1998), are ecological counterparts to the Leptanillinae in the New World. This hypothesis is further supported by remarkable homoplasy between the Amblyoponinae and Leptanillinae, which resulted in the erroneous hypothesis that these clades were akin (Bolton 1990b, 2003).

*Typhlomyrmex* (Ectatomminae: Ectatommini), which are minute hypogaeic ants precinctive to the Neotropics, are also worth noting here on account of the leptanilloid gestalt of the worker. Coarse but pronounced resemblance in habitus implies functional parallels in *Typhlomyrmex* with the Leptanillinae, with the articulated meso-metapleural suture that is unique to *Typhlomyrmex* among the Ectatomminae (Bolton 2003) recalling that feature in *Protanilla* and certain *Leptanilla* species, while the tergosternal fusion of abdominal segment II constitutes convergence with the Leptanillini. Miniaturized and flexible relative to the robust, epigaeic members of their sister clade, *Gnamptogenyssensu stricto* (Camacho et al. 2022), *Typhlomyrmex* represent Ectatomminae that occupy a morphospace occupied outside the New World by the Leptanillinae.

## Supplementary Material

XML Treatment for Protanilla wallacei

XML Treatment for Leptanilla belantan

XML Treatment for Leptanilla acherontia

XML Treatment for Leptanilla bethyloides

XML Treatment for Leptanilla najaphalla

XML Treatment for Leptanillinae

XML Treatment for Opamyrmini

XML Treatment for Opamyrma

XML Treatment for Leptanillini

XML Treatment for Protanilla

XML Treatment for Leptanilla

## Footnotes

These morphospecies are recovered distantly within the Protanilla rafflesi species group by phylogenomic inference (pers. obs.), and so are presumed to be allospecific in the absence of further evidence.

## ﻿Additional information

### Author ORCIDs

Zachary Griebenow https://orcid.org/0000-0003-3385-8479

### Data availability

All of the data that support the findings of this study are available in the main text or Supplementary Information.

## Supplementary materials

Supplementary material 1 Relevant collection data for specimens included in this study, if not previously reported in Griebenow (2020, 2021) or Griebenow et al. (2022) Data type xlsx https://binary.pensoft.net/file/968363This dataset is made available under the Open Database License (http://opendatacommons.org/licenses/odbl/1.0/). The Open Database License (ODbL) is a license agreement intended to allow users to freely share, modify, and use this Dataset while maintaining this same freedom for others, provided that the original source and author(s) are credited.Author: Zachary Griebenow
